# ARHGAP24 represses β-catenin transactivation-induced invasiveness in hepatocellular carcinoma mainly by acting as a GTPase-independent scaffold

**DOI:** 10.7150/thno.72134

**Published:** 2022-08-21

**Authors:** Wenjing Yang, Beili Wang, Qian Yu, Te Liu, Tong Li, Tongtong Tian, Anli Jin, Lin Ding, Wei Chen, Hao Wang, Jingrong Xian, Baishen Pan, Jian Zhou, Jia Fan, Xinrong Yang, Wei Guo

**Affiliations:** 1 Department of Laboratory Medicine, Zhongshan Hospital, Fudan University, Shanghai 200032, P. R. China.; 2 Department of Liver Surgery & Transplantation, Liver Cancer Institute, Zhongshan Hospital, Fudan University; Key Laboratory of Carcinogenesis and Cancer Invasion, Ministry of Education, Shanghai 200032, P. R. China.; 3 Cancer Center, Zhongshan Hospital, Fudan University, Shanghai 200032, P. R. China.; 4 Department of Laboratory Medicine, Xiamen Branch, Zhongshan Hospital, Fudan University, Xiamen 361015, P. R. China.; 5 Department of Laboratory Medicine, Wusong Branch, Zhongshan Hospital, Fudan University, Shanghai 200940, P. R. China.; 6 Shanghai Geriatric Institute of Chinese Medicine, Shanghai University of Traditional Chinese Medicine, Shanghai 200031, P. R. China.

**Keywords:** HCC, invasion, ARHGAP24, PKM2 degradation, β-catenin transactivation

## Abstract

**Rationale:** Accumulating evidence shows that Rho-GTPase-activating proteins (RhoGAPs) exert suppressive roles in cancer cell proliferation and metastasis. However, no study has systematically investigated the clinical significance of RhoGAPs and analyzed the functions of ARHGAP24 in hepatocellular carcinoma (HCC).

**Methods:** The relationship between RhoGAP expression and HCC prognosis was investigated via using The Cancer Genome Atlas and Gene Expression Omnibus databases. ARHGAP24 expression was detected by reverse transcription-polymerase chain reaction, western blot and immunohistochemistry staining assays. Moreover, *in vitro* assays including cell counting kit-8, colony formation, wound healing and Transwell assays, and *in vivo* tumor growth and pulmonary metastases evaluations were conducted to evaluate the biological function of ARHGAP24 in HCC. Liquid chromatography-tandem mass spectrometry, co-immunoprecipitation, GTPase activation, ubiquitination, and luciferase reporter assays and bioinformatics analysis were carried out to gain insights into the mechanisms underlying the tumor-suppressive function of ARHGAP24.

**Results:** ARHGAP24 expression was dramatically decreased in HCC tissues, and low ARHGAP24 expression was an independent poor prognostic indicator for progression-free survival in HCC patients. *ARHGAP24* overexpression significantly inhibited cell proliferation, migration and invasion, while knockdown of *ARHGAP24* exerted the opposite effects. Through Gene Set Enrichment Analysis (GSEA), we found ARHGAP24 mainly suppressed HCC cell proliferation and invasion by attenuating β-catenin transactivation and blocking β-catenin signaling could effectively abolish the promotional effects of ARHGAP24 knockdown in HCC cells. Notably, GAP-deficient mutant of ARHGAP24 exerted similar inhibitory effects as the wild-type did, indicating suppressive function of ARHGAP24 was independent of its RhoGAP activity. Moreover, we identified pyruvate kinase M2 (PKM2) as a new binding partner of ARHGAP24, which recruited a novel E3 ligase (WWP1) and subsequently promoted PKM2 degradation. *WWP1* knockdown significantly reduced the inhibitory function of ARHGAP24, and the C-terminal fragments of ARHGAP24 (amino acids 329 - 430 and 631 - 748) bound directly to WWP1 and PKM2 (amino acids 388 - 531), respectively.

**Conclusions:** Our data indicate that ARHGAP24 may be an independent prognostic indicator for HCC. It is a critical suppressor of HCC that recruits WWP1 for PKM2 degradation. Targeting the ARHGAP24/WWP1/PKM2/β-catenin axis may provide new insights into HCC prevention and treatment.

## Introduction

Hepatocellular carcinoma (HCC) is the main type of liver cancer, accounting for approximately 85% - 90% of all cases. It is the fifth most common malignant tumor in China with the third highest mortality among all cancer types 1-2, and thus presents a serious threat to health and quality of life. At present, radical resection is the only cure for liver cancer, but patients with early-stage HCC have the opportunity to undergo surgery. Although the clinical application of molecular targeted drugs and immune drugs has greatly improved the survival of patients with HCC 3-4, the 5-year metastasis and recurrence rates of HCC remain high, reaching 50% - 70% 5. These important factors therefore restrict the prognosis of HCC patients. Further in-depth exploration of the molecular basis of HCC progression is thus needed to improve patient outcomes.

The identification of new tumor suppressor genes is of great significance for understanding the molecular mechanism of liver cancer progression and for exploring new intervention strategies. The Rho-GTPase-activating protein (RhoGAP) family is a class of emerging tumor suppressors with more than 60 members 6-8. RhoGAPs have been reported to be involved in regulating Rho GTPase (i.e., RhoA, Rac1 and CDC42) activity. Rho GTPases activate a diverse array of downstream effectors, while the GDP-bound states have the opposite effects. RhoGAPs suppress the formation of the active GTP-bound state of Rho GTPases by catalyzing the exchange of GTP for GDP. The aberrant activation or overexpression of RhoGAPs may thus inhibit tumor growth 6-8. However, less than half of all RhoGAPs currently have clear biological functions and the roles of most members of this family are still unclear, especially in highly heterogeneous tumors such as HCC. Further systematic research is therefore urgently needed.

RhoGAP 24 (ARHGAP24) is a member of the RhoGAP protein family 9-10 with strong tumor suppressor potential. Zhang et al. reported that it induced G0/G1 phase arrest of colorectal cancer cells by regulating the expression of p53 and p21 and promoted tumor cell apoptosis 11. ARHGAP24 also inhibited the activation of signal transducer and activator of transcription 6 signaling in lung cancer cells and induced tumor cell apoptosis and inhibited cell proliferation through the WWP2/p27 pathway 12. ARHGAP24 also inhibited the growth of kidney cancer, breast cancer, and astrocytoma, and its low expression can be used as a predictor of poor prognosis in patients with these tumors 13-15. Previous studies found that the single nucleotide polymorphism locus rs346473 of *ARHGAP24* was closely related to susceptibility to hepatitis B virus and the progression of related diseases in the Chinese population 16. However, the biological role of ARHGAP24 in HCC has not yet been explored. In-depth analysis of its specific molecular mechanisms will provide a new theoretical basis for preventing metastasis and recurrence of HCC and for exploring therapeutic targets.

In this study, we investigated the relationship between RhoGAP expression and the prognosis of HCC using data from The Cancer Genome Atlas (TCGA) and Gene Expression Omnibus (GEO) databases. We found that ARHGAP24 expression was reduced in HCC tissues and its expression was significantly related to a poor prognosis compared with other RhoGAPs. Our clinical data further confirmed that ARHGAP24 was an independent indicator predicting time to tumor recurrence (TTR). In addition, *in vivo* and *in vitro* experiments showed that high ARHGAP24 levels could inhibit cancer cell proliferation, migration and invasion. Co-immunoprecipitation combined with mass spectrometry showed that ARHGAP24 could serve as a scaffolding protein to promote the binding of the E3 ubiquitin ligase WWP1 to pyruvate kinase M2 (PKM2), and then degrade PKM2 through the ubiquitin-proteasome pathway to inhibit liver cancer invasion and metastasis.

## Materials and Methods

### HCC patients and follow-up

HCC tissues and adjacent tissues were obtained from 131 adult patients who underwent surgery at Zhongshan Hospital, Fudan University (Shanghai, China) between April 2018 and July 2019. All tumors were histologically confirmed according to the American Association for the Study of Liver Diseases guidelines. None of the patients received preoperative chemotherapy or radiotherapy. In addition, 20 pairs of frozen HCC and non-tumor tissues, eight recurrent tumors and seven non-recurrent tumors were collected after surgical resection. This study was approved by the ethics committee of Zhongshan Hospital, Fudan University. Written informed consent was obtained from all subjects. PFS was set as the endpoint of follow-up in our study. PFS was defined as the interval between resection and intrahepatic recurrence or extrahepatic metastasis. Follow-up ended on 31^st^ July 2021.

### Liquid chromatography-tandem mass spectrometry (LC-MS/MS)

HCCLM3 cells transfected with FLAG-ARHGAP24 were immunoprecipitated with anti-FLAG antibody. Proteins interacting with ARHGAP24 were identified as the experimental group and proteins interacting with IgG were identified as the non-specific binding. All MS experiments were performed on a Thermo Fusion Lumos mass spectrometer connected to an Easy-nLC 1200 via an Easy Spray (Thermo Fisher Scientific, MA, USA). The resulting sequences were searched against the UniProt Human Proteome database (downloaded 5 May 2018). The candidate proteins are listed in **Table S1**.

### RhoGTPase activity detection

Levels of GTP-bound Rac1, GTP-bound CDC42 and GTP-bound RhoA were detected using an Active Rho Detection Kit (Active Rac1 Detection Kit; Active CDC42 Detection Kit; Cell Signaling Technology) according to the manufacturer's protocol. Briefly, GST-Rhotekin-RBD fusion protein or GST-PAK-PBD was used to bind activated forms of GTP-bound Rho and GTP-bound Rac1/CDC42, which were then immunoprecipitated with glutathione resin. The level of Rho activation or Rac1/CDC42 activation was then determined by western blotting using Rho/Rac1/CDC42 rabbit antibody, respectively.

### Statistical analyses

Statistical analyses were performed using SPSS 20.0 software. Continuous variables were presented as mean ± standard deviation. Differences between groups were analyzed by two-tailed unpaired Student's *t*-test, Pearson's χ^2^ test, Mann-Whitney *U* test, two-way ANOVA, or log-rank test. Differences were considered significant at P < 0.05.

Further details of the methods are presented in the Supplementary Material.

## Results

### Identification of ARHGAP24 as a novel prognostic biomarker for HCC

**Figure 1:**
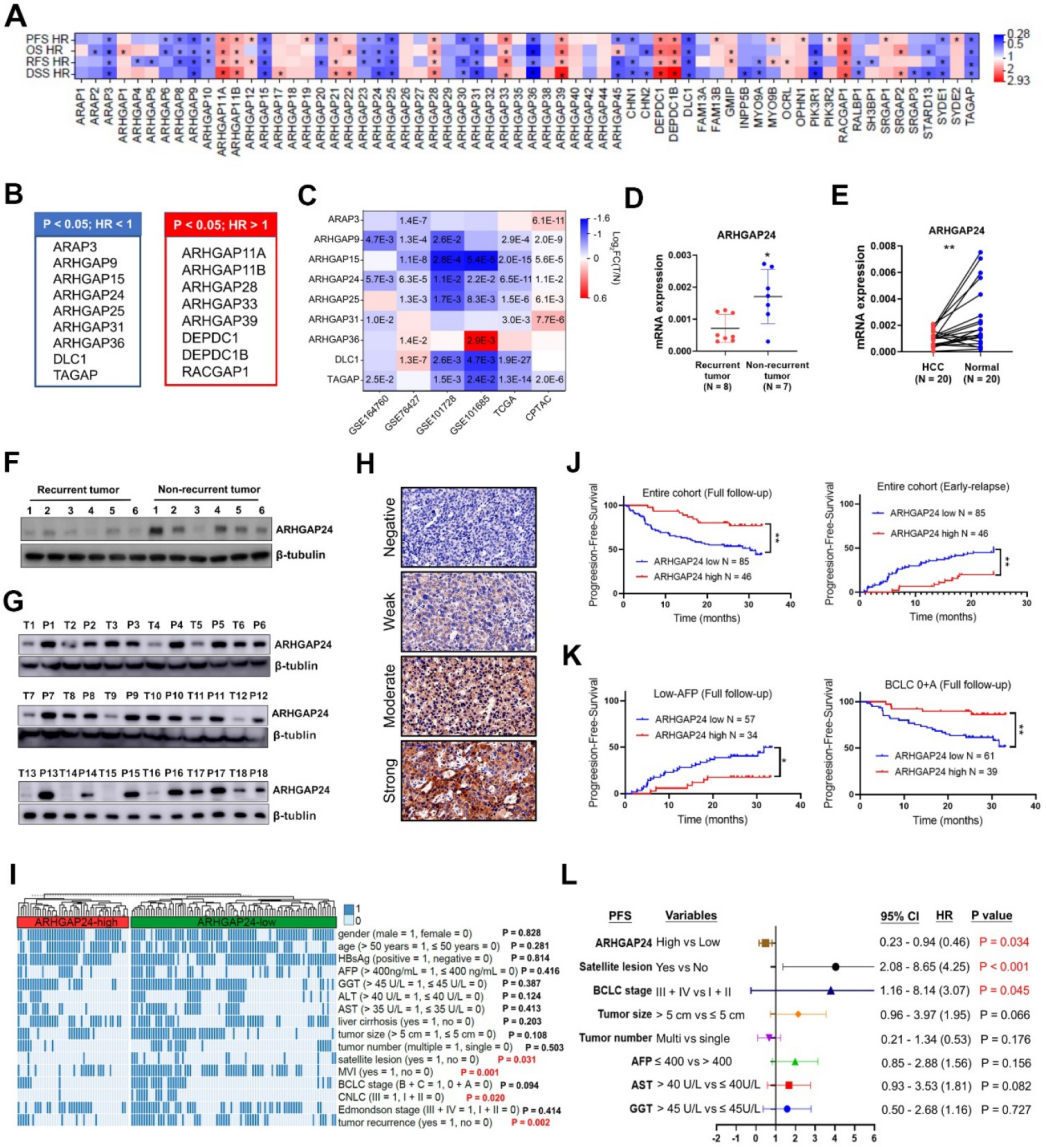
** ARHGAP24 is downregulated in Hepatocellular carcinoma (HCC) and predicts poor prognosis. A.** Prognostic significance of RhoGAP family members in predicting progression-free survival (PFS), overall survival (OS), relapse-free survival (RFS) and disease-specific survival (DSS) according to K-M plotter database. **B.** Lists of RhoGAPs as significant hazard factor (all HR > 1, left) or protective factor (all HR < 1, right) for prognosis in HCC according to K-M plotter database. **C.** Comparisons of indicated RhoGAP family members expressions between tumoral and normal liver tissues according to GEO databases, TCGA and CPTAC datasets. **D.** Detection of ARHGAP24 mRNA expression in primary tumoral tissues from patients with recurrent and non-recurrent HCC by qRT-PCR. **E.** The mRNA expression of ARHGAP24 in paired HCC tissues and adjacent non-tumor tissues. **F.** Detection of protein expression of ARHGAP24 in tumoral tissues from patients with recurrent and non-recurrent HCC by immunoblotting assays. **G.** The protein expression of ARHGAP24 in paired HCC tissues (T) and adjacent non-tumor tissues (P). **H.** Representative immunohistochemistry staining of ARHGAP24 with different intensity. **I.** Heatmap demonstrating the association between ARHGAP24 expression and clinicopathologic features. Chi-square test. **J.** Kaplan-Meier analysis of the progression-free survival (PFS, left) and early-relapse (right) in HCC patients stratified by ARHGAP24 expression. **K.** Kaplan-Meier analysis of the progression-free survival (PFS) in HCC patients with tumor early stage (BCLC: 0 + A, right) and with low AFP levels (≤ 400, left), respectively. **L.** Multivariate Cox analysis of prognostic factors associated with tumor progression in HCC.

The prognostic values of the 64 RhoGAP members were investigated systematically by K-M plotter analysis. Seventeen members were significantly associated with all four clinical outcomes, including overall survival (OS), progression-free survival (PFS), relapse-free survival (RFS) and disease-specific survival (DSS) (all P < 0.05; **Figure 1A**). Among above 17 RhoGAP members, 8 members were classified as hazard indicator for HCC prognosis (hazard ratio (HR) > 1), while 9 members including ARHGAP24 protein were considered as protective factors (HR < 1, **Figure 1B, Figure S1A-D**). Because RhoGAPs were conventionally considered to have capacities on inhibiting tumor growth, we selected members with HR < 1 for further analysis. Only *ARHGAP24* expression was significantly reduced in HCC tissues compared with normal liver tissues in all GEO (GSE164760, GSE76427, GSE101728 and GSE101685), TCGA and CPTAC databases enrolled (all P < 0.05, **Figure 1C**). We further confirmed experimentally that *ARHGAP24* mRNA levels were significantly decreased in patients with recurrent tumors compared with patients with non-recurrent tumors (**Figure 1D**). Moreover, *ARHGAP24* mRNA levels were also dramatically reduced in HCC tissues when compared to paired non-cancerous tissues (**Figure 1E**). Western blotting assays consistently showed that ARHGAP24 protein levels were downregulated in recurrent tumors and HCC tissues (**Figure 1F-G**). We further validated the prognostic value of ARHGAP24 in HCC by immunohistochemistry staining in 131 HCC patients. Representative images were shown in **Figure 1H**. We compared different clinicopathological features of HCC patients and found that ARHGAP24 downregulation was significantly correlated with satellite lesions (P = 0.031), CNLC stage (P = 0.020), microvascular invasion (P = 0.001) and tumor recurrence (P = 0.002) (**Figure 1I, Table S2**). Besides, patients with low ARHGAP24 expression had significantly shorter PFS compared with those who with high-ARHGAP24 expression (P < 0.01; **Figure 1J, left**). Additionally, patients with low ARHGAP24 expression also had significantly higher early-relapse rate (within 2 years; P < 0.01,** Figure 1J, right**). Notably, patients with low ARHGAP24 in early tumor stage (BCLC: 0 + A; P < 0.01) or low alpha-fetoprotein subgroups (≤ 400 ng/mL; P < 0.05) also had higher probabilities of tumor progression when compared to the patients with high ARHGAP24 (**Figure 1K**). Similar results were observed in patients with small tumors (< 5 cm), single tumors, early tumor differentiation and stage, without satellite lesions and without microvascular invasion (**Figure S2A-B**). Univariate Cox regression analysis showed that ARHGAP24, tumor size, BCLC stage and other clinical parameters were associated with tumor progression in HCC patients (**Figure S2C, Table S3**). These factors were further analyzed by multivariate analysis, which revealed that high ARHGAP24 expression in HCC cells was an independent predictive indicator for tumor progression (HR 0.46 (0.23 - 0.94), P = 0.034; **Figure 1L**).

Interestingly, low *ARHGAP24* was also associated with shorter OS in patients with other tumors, such as renal cell carcinoma (P < 0.001), lung cancer (P < 0.05) and pancreatic ductal adenocarcinoma (P = 0.045) (**Figure S3A-E**), suggesting its common inhibitory role in tumors.

### ARHGAP24 inhibited cell proliferation and induced G0/G1 arrest in HCC

**Figure 2:**
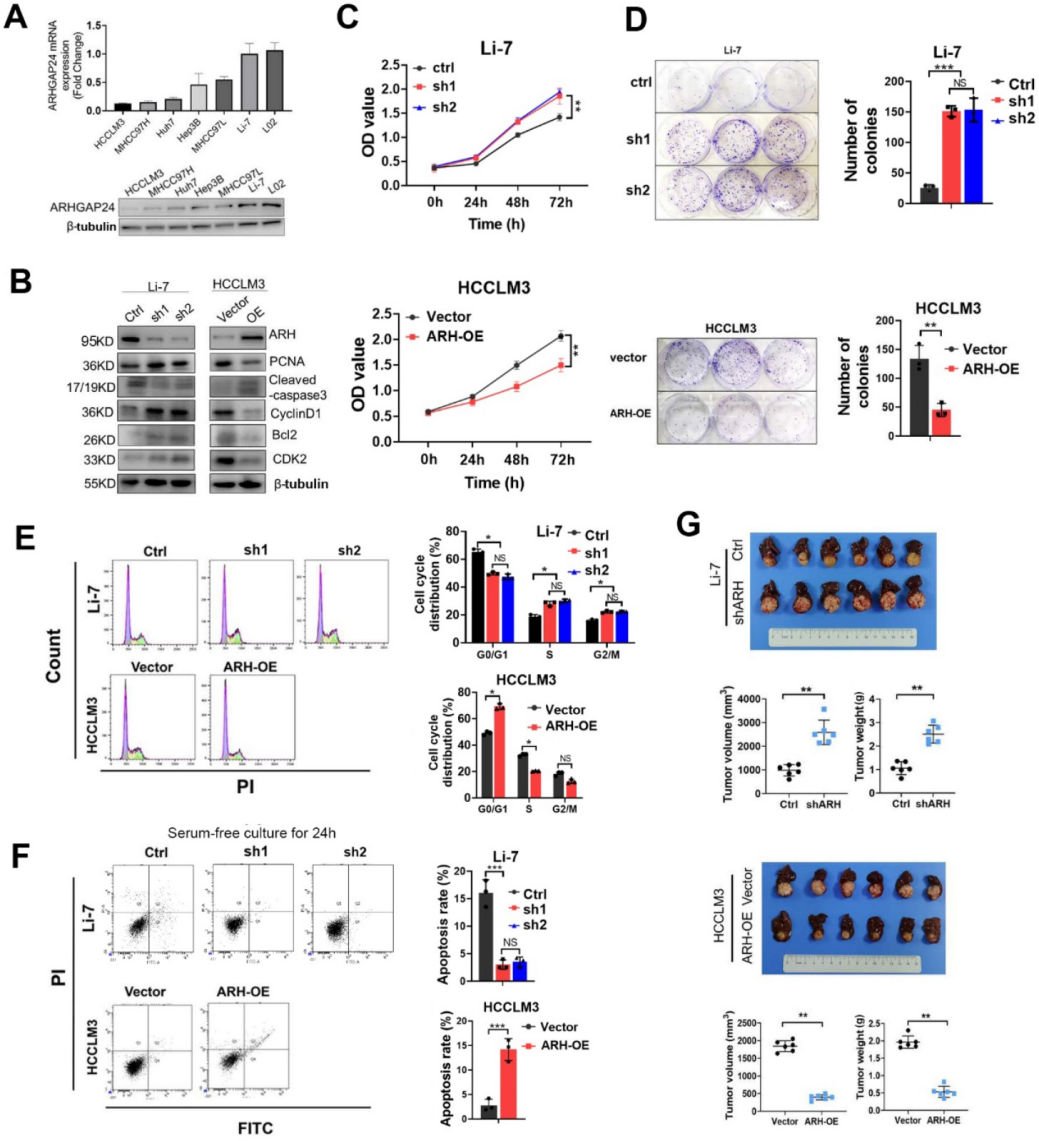
** ARHGAP24 inhibits proliferation potentials in HCC. A.** mRNA (upper panel) and protein (lower panel) expression of ARHGAP24 in indicated cell lines were detected by qRT-PCR and WB assays, respectively. **B.** Alterations of proliferation related molecules after ARHGAP24 expression modulations were detected by WB assays. **C.** Effects of ARHGAP24 overexpression or downregulation on the short-term proliferation potentials were assessed by CCK-8 assays. **D.** Effects of ARHGAP24 overexpression or downregulation on the long-term proliferation potentials were assessed by colony-formation assays. **E.** Effects of ARHGAP24 overexpression or downregulation on cell cycle were determined via flow cytometry assays. **F.** Effects of ARHGAP24 overexpression or downregulation on cell apoptosis were evaluated by flow cytometry assays.** G.** *In vivo* inhibitory efficiency of ARHGAP24 on HCC growth was evaluated by constructing orthotopic xenograft models, and representative images, tumor volumes and tumor weights were illustrated.

ARHGAP24 expression was determined in six HCC cell lines and one normal liver cells. Quantitative reverse transcription-polymerase chain reaction (qRT-PCR) and western blotting (WB) assays showed that ARHGAP24 was downregulated in HCC cell lines with strong metastatic ability (HCCLM3 and MHCC97H), but was highly expressed in cells with weak metastatic potential (MHCC97L and Li-7) (**Figure 2A**). To investigate the functional role of ARHGAP24 in HCC proliferation, we induced stable overexpression of *ARHGAP24* in HCCLM3 cells and Huh7 cells (ARH-OE) and stable silencing of *ARHGAP24* in Li-7 cells (sh1 and sh2). Western blot assays showed successful overexpression and knockdown of *ARHGAP24* expression, respectively (**Figure 2B, Figure S4A**). Notably, protein levels of cell proliferation and anti-apoptosis markers (PCNA, Bcl2, cyclin D1 and CDK2) were significantly reduced in *ARHGAP24*-overexpressing cells while increased in *ARHGAP24*-silenced cells, while the pro-apoptosis marker, caspase-3, exhibited the opposite results (**Figure 2B, Figure S4A**). Cell Counting Kit-8 and colony-formation assays showed that knockdown of *ARHGAP24* in Li-7 cells significantly increased proliferation, while overexpression of *ARHGAP24* in HCCLM3 cells had the opposite effect (**Figure 2C-D**). Similarly, proliferation was reduced in *ARHGAP24*-overexpressing Huh7 cells (**Figure S4B-C**). Additionally, cell cycle assays demonstrated that *ARHGAP24* knockdown accelerated the cell cycle in Li-7 cells, while *ARHGAP24* overexpression in HCCLM3 and Huh7 cells resulted in G0/G1 arrest (**Figure 2E, Figure S4D-F**). Apoptosis assays further showed that silencing *ARHGAP24* prevented apoptosis in HCC cells under serum-free culture for 24 h, while* ARHGAP24* overexpression induced apoptosis in HCCLM3 and Huh7 cells (**Figure 2F, Figure S4G**). Moreover, analysis of liver orthotopic xenograft tumors further confirmed that *ARHGAP24* knockdown promoted tumor growth *in vivo*, as evidenced by increased tumor volume and weight (P < 0.01). Conversely, the volumes of tumor xenografts derived from HCCLM3 vector control and HCCLM3* ARHGAP24* overexpressing cells were 1845.22 ± 152.17 and 398.25 ± 73.89 mm^3^, respectively (P < 0.01; **Figure 2G),** suggesting that* ARHGAP24* overexpression markedly inhibited tumor growth.

### ARHGAP24 attenuated cell invasion and tumor metastasis in HCC

**Figure 3:**
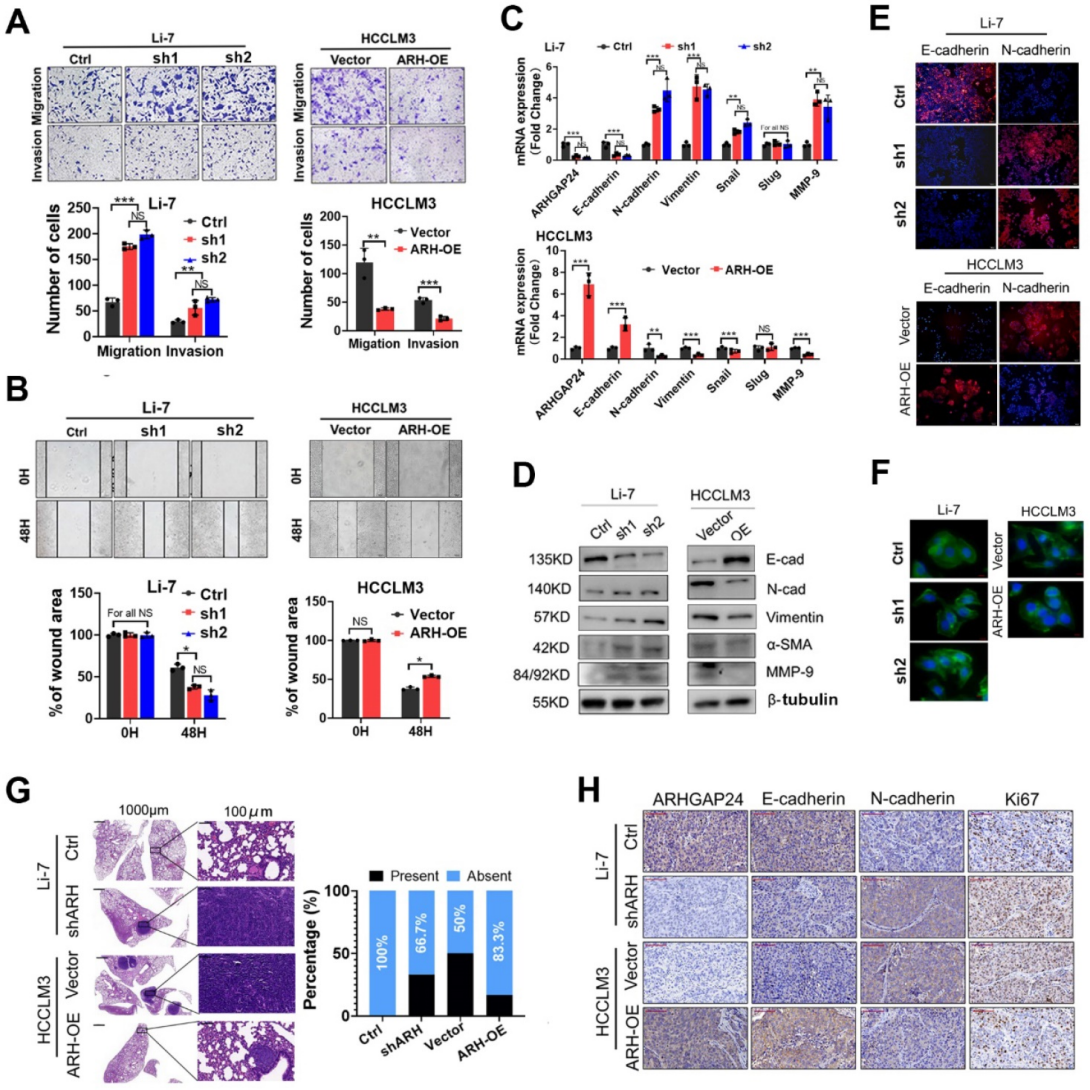
** ARHGAP24 restrains invasiveness and metastasis in HCC. A.** Effects of ARHGAP24 on migrative and invasive capacities of HCC cells were measured by Transwell assays.** B.** Effects of ARHGAP24 on cell migration were evaluated by scratch wound healing assays. **C.** The mRNA expressions of indicated EMT-related markers were detected by qRT-PCR assays. **D.** The protein expressions of EMT-related markers were determined by WB assays. **E.** Expressions of E-Cadherin and N-Cadherin in HCC cell lines after ARHGAP24 expression modulation were evaluated by immunofluorescence staining. **F.** Cell morphology alterations after ARHGAP24 expression modulations were assessed by FITC-phalloidin staining. **G.** Representative images of H&E-stained lung tissues (left), and incidence of lung metastasis in indicated groups (right). **H.** Representative IHC images of ARHGAP24 and indicated markers in tumor tissues derived from orthotopic xenograft models.

Given the correlation between *ARHGAP24* expression and microvascular invasion, we hypothesized that *ARHGAP24* might play an important role in HCC metastasis. To verify this, we investigated HCC cell migration and invasion *in vitro* using transwell and wound-healing assays, respectively. The results revealed that knockdown of *ARHGAP24* increased the numbers of migrating and invading cancer cells, while *ARHGAP24* overexpression decreased these cells (**Figure 3A-B, Figure S5A-B**). In addition, we detected epithelial-mesenchymal transition (EMT)-related mRNA and protein expression in HCC cell lines. *ARHGAP24* knockdown resulted in increased mesenchymal expressions (N-cadherin, vimentin, and MMP-9), while overexpression of *ARHGAP24* resulted in an epithelial-like molecular phenotype (**Figure 3C-D**). Consistently, overexpression of *ARHGAP24* in Huh7 cells also inhibited mesenchymal-like molecular phenotype (**Figure S5C-D**). Immunofluorescence analysis confirmed that downregulation of *ARHGAP24* decreased E-cadherin expression but increased N-cadherin expression in Li-7 cells, while overexpression of* ARHGAP24* in HCCLM3 cells produced the opposite effects (**Figure 3E**). Moreover, phalloidin staining showed that HCCLM3 cells evolved from a mesenchymal to an epithelial morphology following *ARHGAP24* overexpression, while Li-7 cells with *ARHGAP24* knockdown changed from their original round shape to a shuttle-like shape (**Figure 3F**).

To verify the* in vitro* experimental results, mice were injected with 5 × 10^6^ HCCLM3 cells via the tail vein to observe the typical lung metastasis sites. The incidence of lung metastasis was reduced (50.00% vs. 16.67%) after *ARHGAP24* overexpression, while the incidence of lung metastasis of Li-7 cells was increased (0.00% vs. 33.33%) after *ARHGAP24* knockdown (**Figure 3G**). In addition, we detected the expression levels of E-cadherin (epithelial marker), N-cadherin (mesenchymal marker) and Ki67 (cell proliferation marker) in an orthotopic liver xenograft mouse model by immunohistochemistry. E-cadherin expression was downregulated and N-cadherin and Ki67 expression levels were upregulated in liver tumor tissues with *ARHGAP24* knockdown compared with the control group. However, we observed the opposite result when *ARHGAP24* was overexpressed (**Figure 3H**). These results indicated that ARHGAP24 inhibited the migration and invasion of liver cancer cells.

### ARHGAP24 regulated HCC progression mainly via inhibiting the Wnt/β-catenin signaling pathway

**Figure 4:**
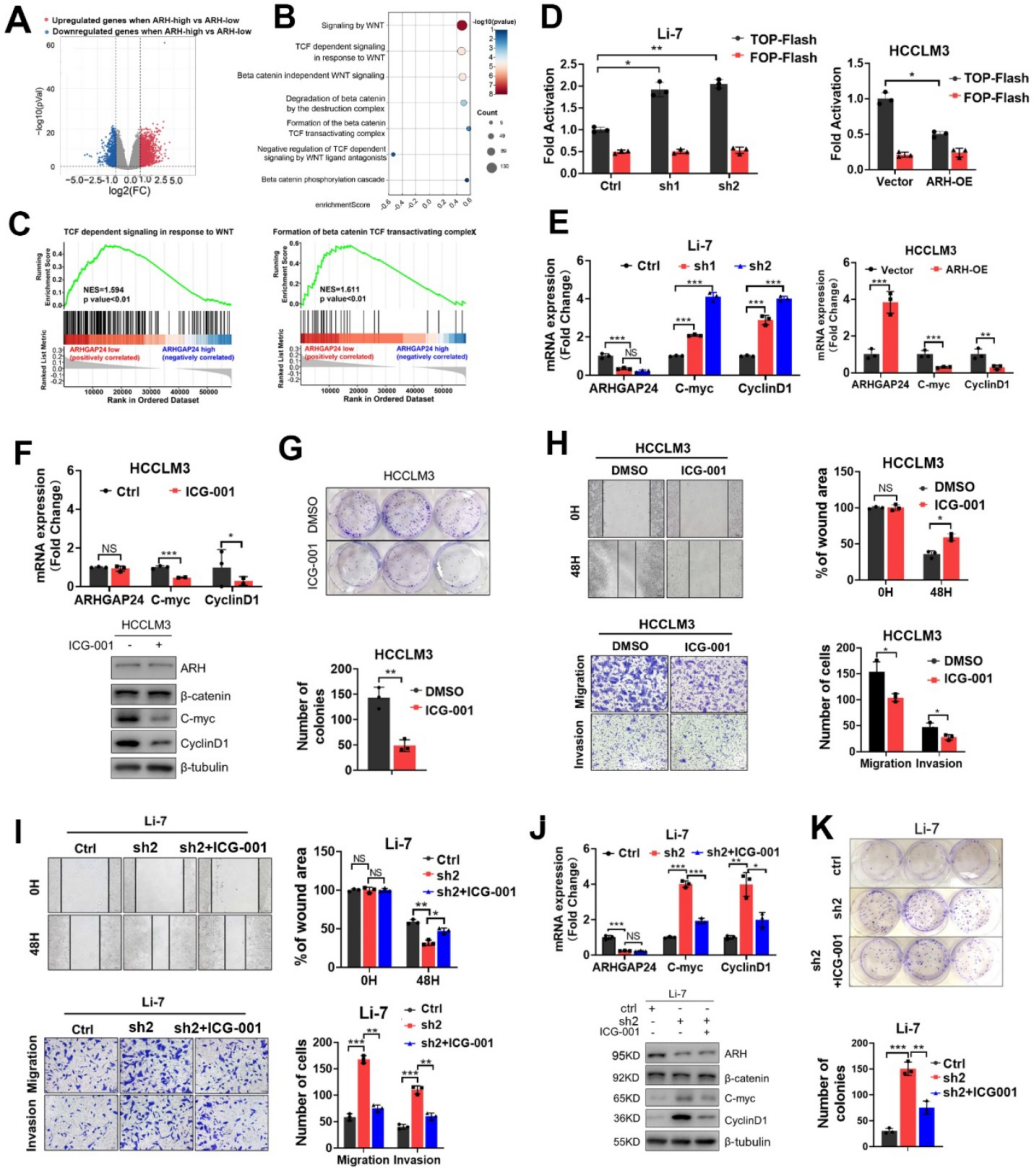
** ARHGAP24 represses the transcriptional activity of β-catenin. A.** Differential genes (DEGs) were identified when ARHGAP24 high group (upper quarter) was compared to ARHGAP24 low group (lower quarter) in TCGA dataset according to follow criteria: p value < 0.05 and |log2 fold change (FC)| > 1. **B.** Bubble plot revealed significantly enriched Wnt/β-catenin-related signaling according to Reactome pathway analysis.** C.** GSEA analysis demonstrated highly activated Wnt/β-catenin signaling especially the transcriptional activity were enriched in ARHGAP24-low subgroup. **D.** The transcriptional activity of β-catenin in indicated cells received distinct treatment was analyzed by TOP/FOP Flash assays.** E.** The mRNA expressions of downstream target genes of β-catenin were detected by qRT-PCR assay. **F.** The mRNA (upper panel) and protein (lower panel) expression of downstream target genes of β-catenin were determined in HCCLM3 cells in the presence of ICG-001, a specific antagonist of Wnt/β-catenin pathway. **G.** Effects of ICG-001 on the long-term proliferation potentials of HCCLM3 cells assessed by colony formation assays. **H.** Effects of ICG-001 treatment on the migration and invasion capacities of HCCLM3 cells were determined by Transwell and scratch wound healing assays. **I.** Effects of ICG-001 on the migration capacities of ARHGAP24-knockdown Li-7 cells were assessed by Transwell and scratch wound healing assays. **J.** mRNA (upper panel) and protein (lower panel) expressions of downstream target of β-catenin in Li-7 cells received indicated treatment were detected by qRT-PCR and WB assays.** K.** Effects of ICG-001 on the proliferation of ARHGAP24-knockdown Li-7 cells were evaluated using colony formation assays.

To further identify the underlying signaling of ARHGAP24 in HCC, HCC patients from TCGA dataset were divided into high- and low-*ARHGAP24* expression groups, according to the upper and lower quartiles of ARHGAP24 expression. Differentially expressed genes (DEGs) were identified as follows: |log2 fold change (FC)| > 1 (ARHGAP24 high verse ARHGAP24 low) and P < 0.05 (**Figure 4A**). Reactome pathway analysis revealed that Wnt/β-catenin signaling pathways were conformably enriched in the low-ARHGAP24 expression group (**Figure 4B**). As Wnt/β-catenin signaling pathway played vital roles in HCC physiological processes, including cell proliferation, migration and invasion, we selected this pathway as the potential candidate for the following investigation. Gene set enrichment analysis (GSEA) showed that enriched pathways related to Wnt/β-catenin pathways were highly activated in the low-ARHGAP24 expression group (**Figure 4C**). Furthermore, *ARHGAP24* knockdown significantly augmented β-catenin transcriptional activity as demonstrated by TOP/FOP Flash reporter assay in Li-7 cells, while its overexpression suppressed β-catenin transcription in HCCLM3 cells (**Figure 4D**). qRT-PCR and western blot assays revealed that silencing *ARHGAP24* enhanced the expression of the downstream target genes, such as *MYC* and *CCND1,* of the Wnt/β-catenin pathway, while* ARHGAP24* overexpression inhibited their expressions (**Figure 4E, Figure S5E**). To validate the critical role of β-catenin transactivation in ARHGAP24-regulated process, we further treated HCCLM3 cells (low-ARHGAP24 expression) and ARHGAP24-knockdown Li-7 cells with ICG-001, a high specific inhibitor of β-catenin transcriptional activity. Expression levels of downstream target genes of β-catenin were reduced in HCCLM3 cells (**Figure 4F**), as shown by qRT-PCR and western blotting assays. Proliferation, migration and invasion capacities were significantly restrained after treatment with ICG-001 in HCCLM3 cells (**Figure 4G-H, Figure S5F**). Notably, the addition of ICG-001 to Li-7 cells alleviated the pro-HCC effects, including the increases in cell proliferation, migration, invasion and β-catenin activity caused by *ARHGAP24* knockdown (**Figure 4I-K, Figure S5G**). Collectively, these data suggested that ARHGAP24 suppressed HCC cell proliferation and invasion mainly by inhibiting the transcriptional activity of β-catenin.

### ARHGAP24 inhibited the transcriptional activity of β-catenin mainly by an enzyme-independent manner

**Figure 5:**
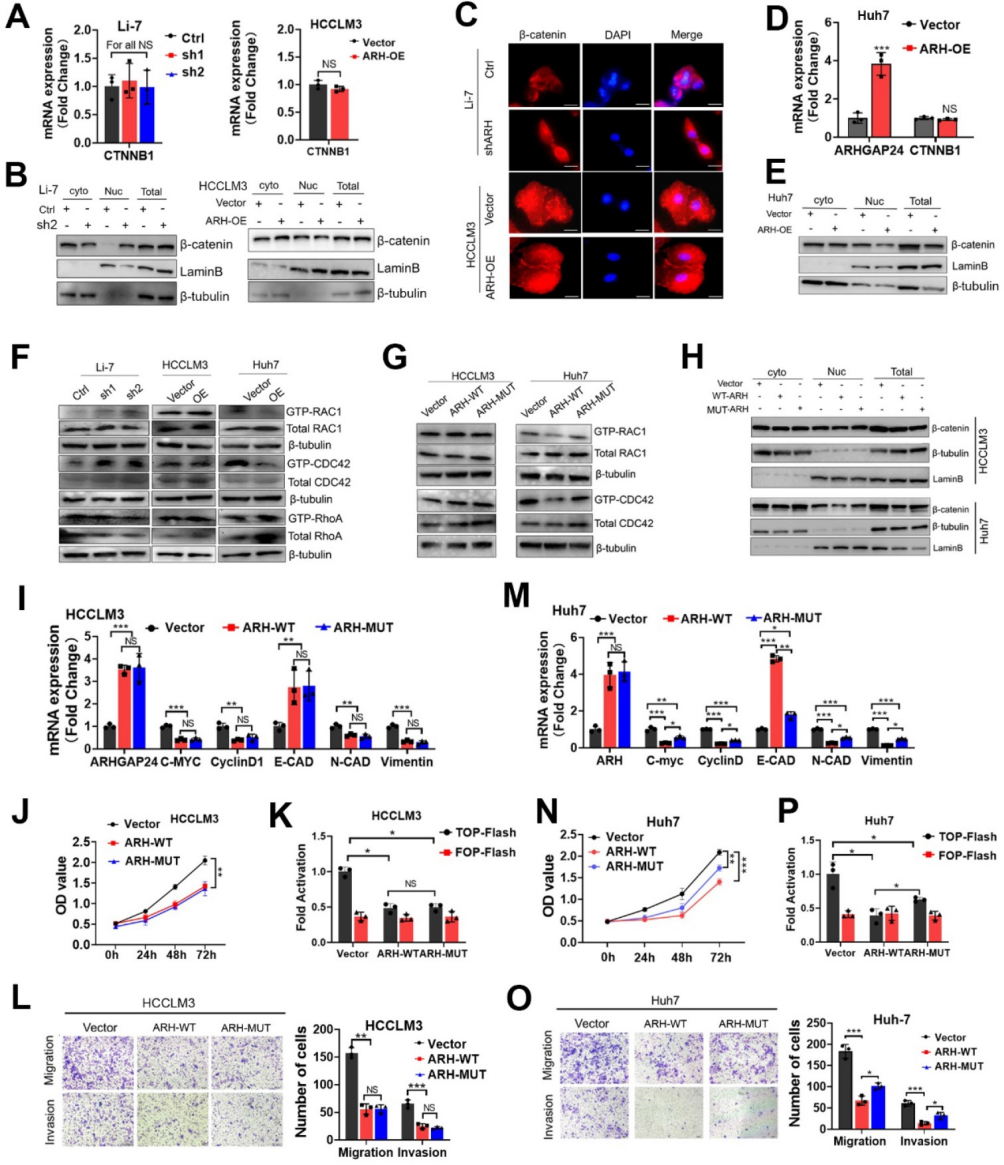
** ARHGAP24 inhibits cell invasiveness mainly through an RhoGAP activity independent manner. A.** The mRNA expression of β-catenin was detected in HCC cells received indicated treatments. **B.** The distribution of β-catenin in nucleus and cytoplasm was detected nucleocytoplasmic separation experiment followed by WB assays. **C.** The distribution of intracellular β-catenin detected by immunofluorescence assay. **D.** The mRNA expressions of ARHGAP24 and β-catenin were detected in ARHGAP24-overexpressed Huh7 cells received indicated treatments. **E.** The intracellular distribution of β-catenin in Huh7 cells was detected by WB assays. **F.** The levels of GTP-bound RAC1, CDC42 and RhoA were determined by PKA-GST pull-down followed by WB assays. **G.** Levels of GTP-bound RAC1 and GTP-bound CDC42 were detected in the ARH-WT overexpressed-cells and ARH-MUT overexpressed cells. **H.** The subcellular distributions of β-catenin in the indicated cells were determined by WB assays.** I.** mRNA expression of downstream target genes of β-catenin was detected by qRT-PCR assay in HCCLM3 cells. **J.** Effects of wild-type ARHGAP24 (ARH-WT) and Q158R mutant ARHGAPP24 (ARH-MUT) overexpression on proliferation potentials of HCCLM3 were determined by CCK8 assays. **K.** The transcriptional activity of β-catenin in ARH-WT and ARH-MUT overexpressed HCCLM3 cells was analyzed by TOP/FOP Flash assay. **L.** Effects of ARH-WT and ARH-MUT overexpression on migration and invasion capacities of HCCLM3 cells were validated by Transwell assays. **M.** mRNA expression of downstream target genes of β-catenin was detected by qRT-PCR assay in Huh7 cells. **N.** Effects of wild-type ARHGAP24 (ARH-WT) and Q158R mutant ARHGAPP24 (ARH-MUT) overexpression on proliferation potentials of Huh7 cells were determined by CCK8 assays. **O.** Effects of ARH-WT and ARH-MUT overexpression on migration and invasion capacities of Huh7 cells were validated by Transwell assays. **P.** The transcriptional activity of β-catenin in ARH-WT and ARH-MUT overexpressed Huh7 cells was analyzed by TOP/FOP Flash assay.

β-catenin expression and localization were critical for the activation of Wnt/β-catenin signaling 17. We further investigated how ARHGAP24 regulated β-catenin signaling by detecting the expression and subcellular distribution of β-catenin. qRT-PCR and western blot assays revealed that knockdown of *ARHGAP24* in Li-7 cells and overexpression of* ARHGAP24* in HCCLM3 cells did not affect β-catenin expression (**Figure 5A-B**). Overexpression of* ARHGAP24* also had no effect on the intracellular distribution of β-catenin in HCCLM3 cells, while knockdown of *ARHGAP24* in Li-7 cells promoted the nuclear accumulation of β-catenin (**Figure 5A-B**). Immunofluorescence assay showed similar results (**Figure 5C**). Moreover, overexpression of *ARHGAP24* in Huh7 cells slightly reduced β-catenin protein expression but not mRNA expression, and also reduced the nuclear distribution of β-catenin (**Figure 5D-E**). Previous studies reported that ARHGAP24 was a Rac-specific RhoGAP, inactivating Rac1 and thereby inhibiting tumor progression 10. We therefore examined the Rho GTPase activity of ARHGAP24 and observed the effects of Rac1 activation on β-catenin transcriptional activity. We pulled-down GTP-Rac1, GTP-RhoA and GTP-CDC42 using GST-tagged fusion protein beads, as a well-recognized approach for evaluating Rho GTPase activity, followed by immunoblotting assays to determine the levels of the GTP-bound fractions of Rac1, RHOA and CDC42 after modulation of *ARHGAP24* expression. The results showed that GTP-RAC1 and GTP-CDC42 levels were decreased in *ARHGAP24*-overexpressing Huh7 cells, whereas knockdown of *ARHGAP24* in Li-7 cells resulted in slight increases in GTP-Rac1 and GTP-CDC42 levels. However, *ARHGAP24* overexpression in HCCLM3 cells had no effects on levels of GTP-RAC1, GTP-CDC42 and GTP-RHOA (**Figure 5F**). Strangely, we observed a phenomenon that ARHGAP24 exhibited common inhibitory effects in HCC which was unparalleled with its inhibitory effects on RAC1. We therefore raised a hypothesis that there might be an unknown but enzyme-independent mechanism in addition to inhibition of canonical RAC1 pathway.

To further verify our hypothesis, we constructed a GAP-deficient mutant (Q158R) of the *ARHGAP24* gene, encoding a protein lacking RhoGAP activity (ARH-MUT, **Figure S6A**), which failed to inhibit RAC1 activation (**Figure 5G**). Biological function assays showed that ARH-MUT protein could also effectively restrain the growth and migration of HCCLM3 cells, and the inhibitory effects were consistent with wild-type ARHGAP24 (ARH-WT) protein (**Figure 5H-L, Figure S6B**). Importantly, despite the loss of function in terms of suppressing Rac1 activity and β-catenin nuclear accumulation (**Figure 5H**), ARH-MUT also exerted inhibitory effects on the proliferation, mesenchymal-like phenotype and invasiveness potential in Huh7 cells as the ARH-WT did (**Figure 5M-O**). Similarly, ARH-MUT expression still successfully restrained β-catenin transactivation and transcriptional activity (**Figure 5P, Figure S6C**). Overall, above findings indicated that, despite the cell-specific function of ARHGAP24 in inhibiting Rho activity in HCC cell lines, ARHGAP24 mainly inhibited the transcriptional activity of β-catenin in a Rho-GTPase-independent manner.

### ARHGAP24 interacted and reduced PKM2 to retrain the transcriptional activity of β-catenin

**Figure 6:**
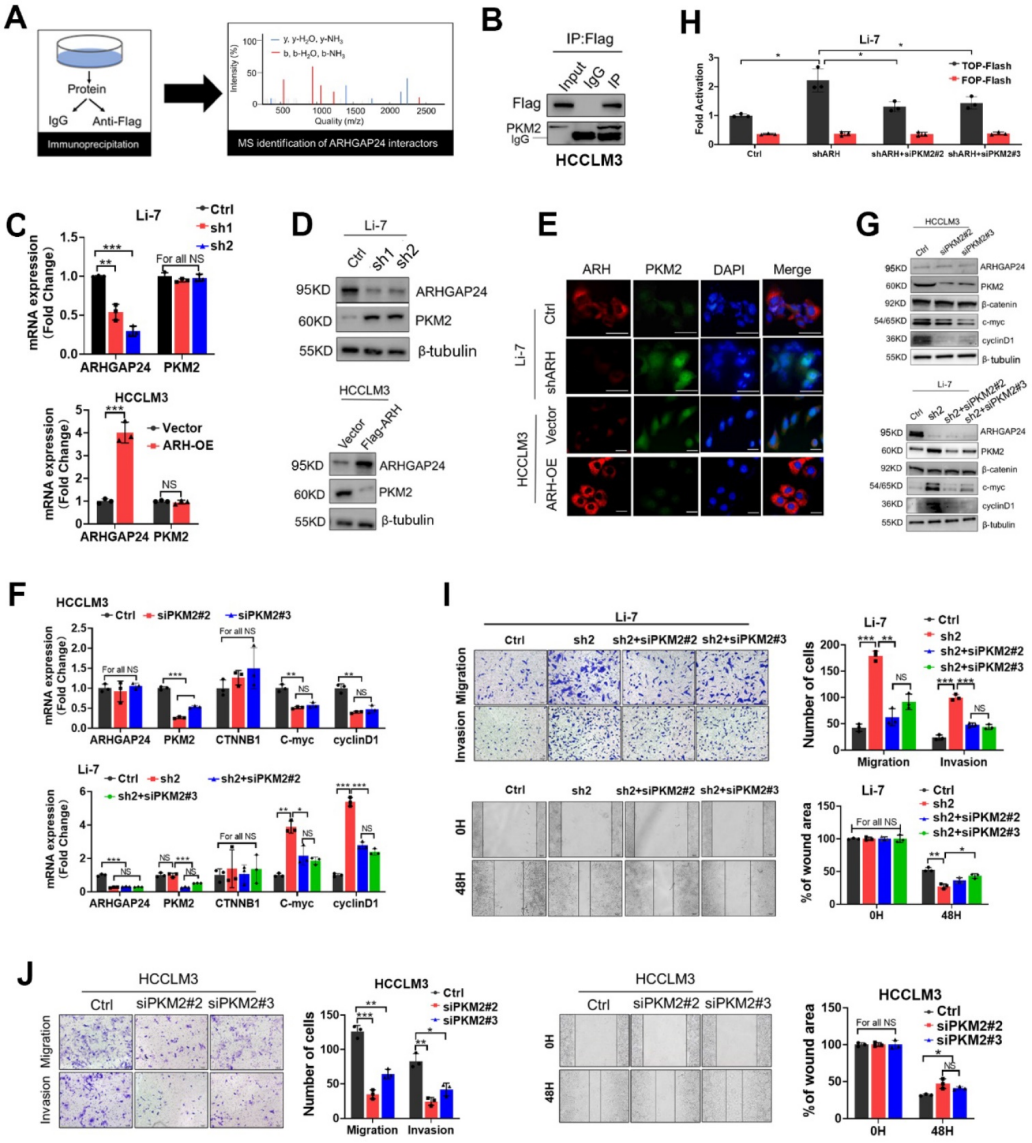
** ARHGAP24 restrains β-catenin signaling via downregulating PKM2 protein abundance. A.** Schematic diagram showing IP-MS strategies for identification of the interactor partner protein of ARHGAP24. **B.** CO-IP analysis revealed strong interaction between ARHGAP24 and PKM2 in HCC cells.** C.** mRNA expression of ARHGAP24 and PKM2 were detected by qRT-PCR.** D.** Protein expression of PKM2 after ARHGAP24 knockdown (upper panel) or overexpression (lower panel) were detected by WB assay. **E.** Effects of ARHGAP24 modulation on PKM2 expression were assessed via using immunofluorescence staining. **F and G.** The effects of silencing PKM2 on mRNA (F) and protein (G) expressions of the downstream targeted genes of β-catenin in ARHGAP24-low (upper panel) or ARHGAP24 knockdown cells (lower panel). **H.** The transcriptional activity of β-catenin in the indicated cells was analyzed by TOP/FOP Flash assay. **I.** The effects of silenced PKM2 on the abilities of cell migration and invasion in Li-7-shARH cells. **J.** The effects of silenced PKM2 on the abilities of cell migration and invasion in HCCLM3 cells.

RhoGAPs shed their enzyme-independent functions mainly by working as a scaffold to facilitate interactions between other proteins to sustain or restrain tumor progression 18-19. We therefore performed LC-MS/MS to identify proteins interacting with ARHGAP24 in ARHGAP24 overexpressed HCCLM3 cells (**Figure 6A**). The results identified pyruvate kinase (PKM) as the top-ranked protein in addition to ARHGAP24 (**Table S1**). Previous studies reported that PKM2 was highly expressed in liver cancer tissues and promoted cancer cell proliferation and invasion. Importantly, PKM2 expression was reported to be the vital enhancer for β-catenin transactivation 20-21. We carried out further immunoprecipitation (IP) assays to confirm the interaction between PKM2 and ARHGAP24 (**Figure 6B**). Interestingly, *PKM2* mRNA expression was not affected by *ARHGAP24* modulation, as shown by qRT-PCR assays (**Figure 6C**); however, PKM2 protein expression was significantly increased after ARHGAP24 knockdwon or decreased after ARHGAP24 overexpression, according to western blotting (**Figure 6D**) and confirmed by immunofluoresence staining (**Figure 6E**). To clarify if the ARHGAP24 modulation of cell proliferation and invasion was dependent on PKM2, we silenced *PKM2* in HCCLM3 and Li-7-shARH cells (**Figure S7A-B**). PKM2 knockdown suppressed the mRNA and protein expression levels of EMT-related markers and downstream target genes of β-catenin in HCCLM3, which mimicked the inhibitory effects of ARHGAP24 overexpression. Notably, interfering PKM2 in Li-7-shARH cells almost abrogated the enhanced expressions of EMT-related markers and downstream target genes of β-catenin caused by ARHGAP24 knockdown, as shown by qRT-PCR and western blot assays (**Figure 6F-G**). Moreover, silencing *PKM2* greatly abolished the increased β-catenin transcriptional activity resulted from ARHGAP24 knockdown, without affecting RAC1 activity (**Figure 6H, Figure S7C**). Functional experiments further revealed that silencing* PKM2* decreased the proliferation and invasion of HCCLM3 as the ARHGAP24 knockdown did, and significantly abolished the promotional effects of ARHGAP24 silence on the invasiveness and growth in Li-7 cells (**Figure 6I-J, Figure S7D-E**). Collectively, these findings revealed that ARHGAP24 suppressed β-catenin transactivation by interacting with PKM2 to decrease its protein expression in HCC.

### ARHGAP24 degraded PKM2 via the ubiquitin-proteasome pathway by recruiting WWP1

**Figure 7:**
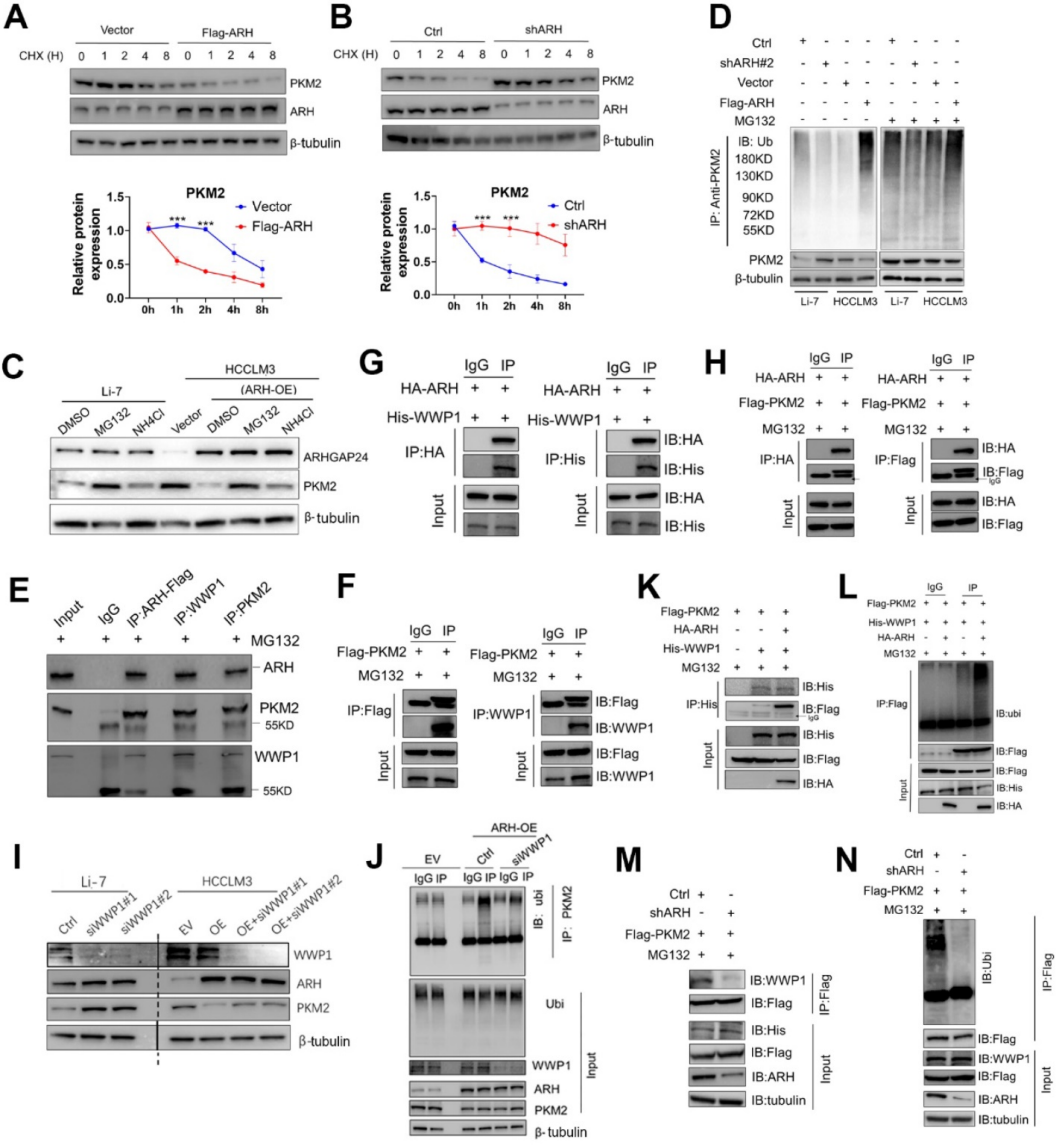
** ARHGAP24 enhances PKM2 ubiquitination by promoting the interaction of PKM2 and WWP1. A.** WB analysis of PKM2 and ARHGAP24 protein in HCCLM3 cells transfected with ARHGAP24 overexpressing plasmid and corresponding control in the presence of CHX. **B.** WB analysis of PKM2 and ARHGAP24 protein in Li-7 cells transfected with ARHGAP24 knocking down plasmid and corresponding control in the presence of CHX. **C**. WB analysis of PKM2 and ARHGAP24 protein in Li-7 cells and ARHGAP24 overexpressed-HCCLM3 cells in the presence of 10 µM MG132 or 10 µM NH4Cl for 8 h. **D.** Ubiquitination assay in HCC cells transfected with the indicated plasmids, followed by WB analysis of indicated proteins.** E.** Co-IP analysis of the interaction of ARHGAP24, WWP1 and PKM2 in HCLCM3 cells in the presence of MG132. **F.** IP and WB analyses of the interaction of PKM2 and WWP1 in Li-7 cells transfected with the indicated plasmids. **G.** IP and WB analyses of the interaction of ARHGAP24 and WWP1 in HEK293T cells transfected with the indicated plasmids. **H.** IP and WB analyses of the interaction of ARHGAP24 and PKM2 in HEK293T cells transfected with the indicated plasmids. **I**. The effects of silenced WWP1 on the protein expression of WWP1, ARHGAP24 and PKM2 by WB analysis. **J.** IP analyses of the PKM2 ubiquitination in ARHGAP24 overexpressed-HCCLM3 cells transfected with the indicated plasmids. **K.** IP analyses of the interaction of Flag-PKM2 and His-WWP1 in the presence and absence of ARHGAP24. **L.** IP analyses of the PKM2 ubiquitination in the presence and absence of ARHGAP24. **M.** The interaction of WWP1 and PKM2 was detected by IP assays in ARHGAP24 knockdown cells or control cells. **N.** IP analyses of the PKM2 ubiquitination in ARHGAP24 knockdown cells or control cells.

Since expression of *PKM2* mRNA was not affected by ARHGAP24 expression modulation, we speculated that ARHGAP24 might exert its function by inhibiting the stability of PKM2 protein. We tested this hypothesis by cycloheximide chase assays to investigate the stability of PKM2. Overexpression of *ARHGAP24* in HCCLM3 cells substantially decreased the half-life of PKM2 protein (**Figure 7A**), while knockdown of *ARHGAP24* in Li-7 cells remarkably increased the half-life of PKM2 (**Figure 7B**). We further explored the mechanism underlying ARHGAP24-induced PKM2 protein degradation by adding the proteasome inhibitor MG132 or the lysosome inhibitor NH_4_CI to the culture medium of Li-7 (high *ARHGAP24* expression) and HCCLM3 cells (*ARHGAP24* overexpression). The results showed that MG132 could effectively rescue the decrease in PKM2 induced by high *ARHGAP24* expression, while NH_4_CI showed weaker effects (**Figure 7C**). Furthermore, *ARHGAP24* knockdown inhibited but *ARHGAP24* overexpression promoted the ubiquitination of PKM2 (**Figure 7D**). These results suggest that ARHGAP24-mediated PKM2 protein decrease mainly via E3 ligase-induced ubiquitination, followed by proteasomal degradation pathway.

We further analyzed the potential ARHGAP24-binding candidate E3 ligases from by Co-IP combined with MS in ARHGAP24-FLAG-overexpressing cells. WWP1 was identified as the only potential E3 ligase candidate. We therefore hypothesized that ARHGAP24-enhanced PKM2 ubiquitination was dependent on WWP1, via formation of a regulator complex. Co-IP analysis confirmed that these three proteins formed a complex in HCCLM3 cells transfected with Flag-tagged ARHGAP24 (**Figure 7E),** and exogenous, Flag-tagged PKM2 could successfully interact with endogenous WWP1 in ARHGAP24-high Li-7 cells (**Figure 7F**). The interactions between ARHGAP24 and PKM2, WWP1 and ARHGAP24 were also confirmed by IP analysis in HEK293T cells (**Figure 7G-H**). Notably, knockdown of *WWP1* abrogated the decrease in PKM2 induced by high ARHGAP24 expression, but expression levels of ARHGAP24 and WWP1 were not affected by each other (**Figure 7I**). ARHGAP24-mediated PKM2 ubiquitination was markedly weakened when *WWP1* was silenced (**Figure 7J**). Furthermore, co-transfection of His-WWP1 plasmids with FLAG-PKM2 and HA-ARH in HEK293 cells promoted WWP1-PKM2 interactions (**Figure 7K**). Consistently, more ubiquitinated PKM2 proteins were immunoprecipitated from cells co-expressing the three plasmids compared with cells co-transfected with WWP1 and PKM2 (**Figure 7L**). In contrast, WWP1-PKM2 interactions and WWP1-mediated PKM2 ubiquitination were markedly weakened by *ARHGAP24* knockdown in Li-7 cells (**Figure 7M-N**). These data collectively indicate that ARHGAP24 acts as a scaffolding protein to potentiate WWP1-mediated PKM2 ubiquitination and degradation.

### C-terminal region of ARHGAP24 was responsible for the scaffolding function

**Figure 8:**
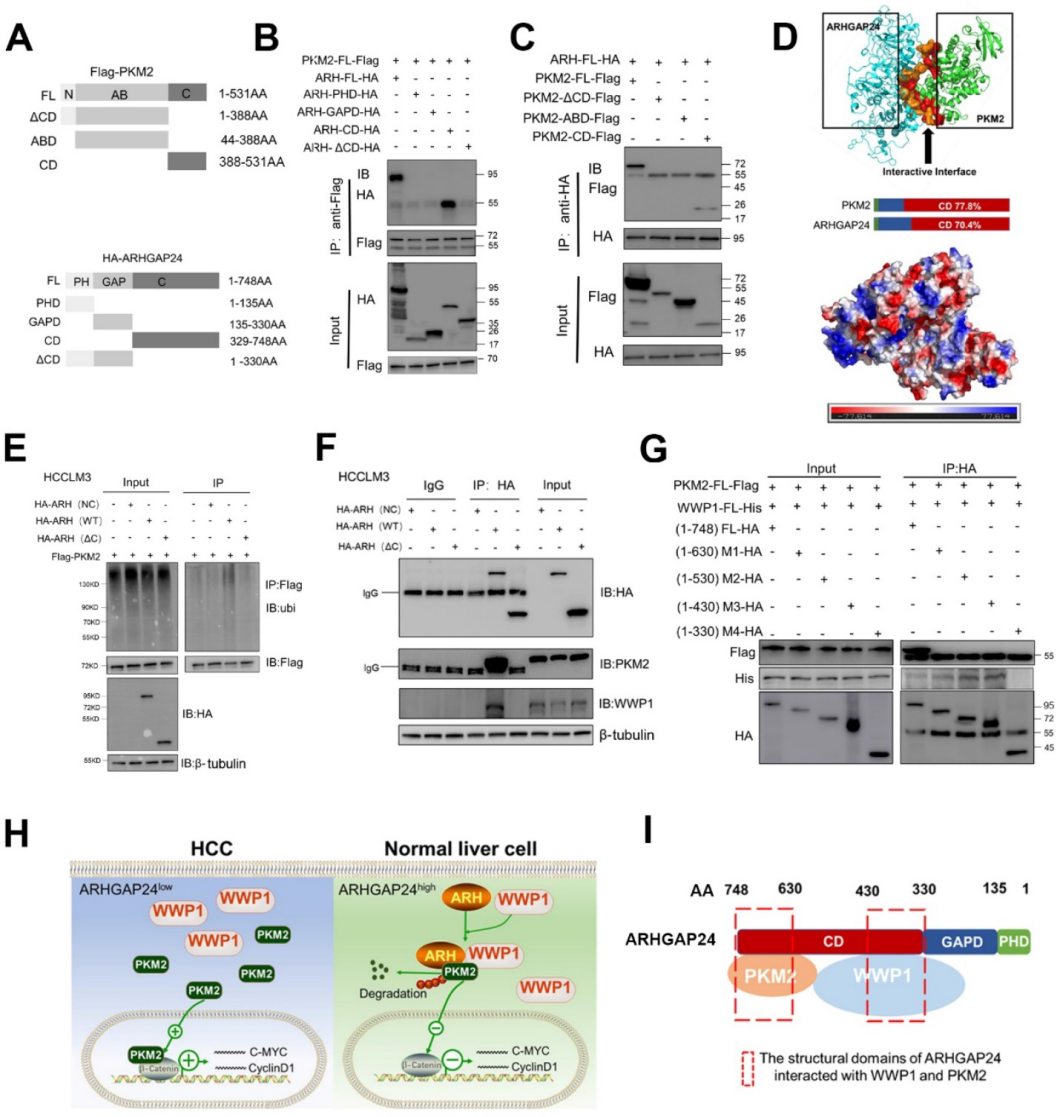
** The C-terminal region of ARHGAP24 for the interaction of WWP1 and PKM2. A.** Schematic representation of Flag-tagged full-length PKM2 and HA-tagged full-length ARHGAP24, along with their various deletion mutants. **B.** HEK293T cells were co-transfected with the full-length PKM2 along with the indicated HA-tagged ARHGAP24 constructs. **C.** HEK293T cells were co-transfected with the full-length ARHGAP24 along with the indicated Flag-tagged PKM2 constructs. The interaction between ARHGAP24 and PKM2 was detected by IP and WB assays. **D.** Representative pictures of the binding domain of the ARHGAP24 and PKM2 structure. **E.** Ubiquitination assays of PKM2 in the presence or absence of ARHGAP24 C-terminal domain in HCCLM3 cells. **F**. IP and WB analyses of the interaction of ARHGAP24, WWP1 and PKM2 in the presence and absence of ARHGAP24 C-terminal domain. **G.** HEK293T cells were co-transfected with the Flag-tagged full-length PKM2 and His-tagged full-length WWP1, along with the indicated ARHGAP24 mutants. **H.** The schematic diagram of the mechanisms of ARHGAP24 on HCC progression. **I.** The structural domains of ARHGAP24 interacted with WWP1 and PKM2.

To further verify the role of ARHGAP24 as a scaffold, we transfected different concentrations of ARHGAP24 plasmids into HCCLM3 and MHCC97H cells, and showed that interaction of PKM2 and WWP1 was increased in *ARHGAP24*-overexpressing cells compared with control cells, as shown by western blot and IP assays (**Figure S8A**), validating the critical role of ARHGAP24 as a scaffold. Deletion mutants of HA-tagged ARHGAP24 and FLAG-tagged PKM2 were constructed to further identify the binding motifs for the ARHGAP24-PKM2 interaction. Representative images were shown in **Figure 8A**. We mapped the domain that accounted for ARHGAP24 binding to PKM2 by generating expression constructs for full-length HA-tagged ARHGAP24 (ARH-FL-HA) and a series of ARHGAP24 mutants lacking different domains, including ARH-PHD, ARH-GAPD, ARH-CD and ARH-ΔCD, and co-expressed these constructs along with full-length FLAG-tagged PKM2. The results indicated that the C-terminal domain of ARH (ARH-CD-HA) was responsible for the interaction with PKM2 (**Figure 8B**). Additionally, we transfected expression vectors encoding ARH-FL-HA or deletion mutants (PKM2-ΔCD, PKM2-ABD, PKM2-CD) and FLAG-tagged PKM2 into HEK293T cells, followed by IP and western blot assays with anti-HA or anti-PKM2 antibody, and showed that the C-terminal of PKM2 interacted with ARHGAP24 (**Figure 8C**). Furthermore, bioinformatics analysis of ARHGAP24-PKM2 interactions revealed that the interaction domains of both proteins were at their respective C-terminal, with a probability > 70% (**Figure 8D**). Representative protein structures of PKM2 and ARHGAP24 and their interaction domains are shown in **Figure S8B**.

To confirm the role of the functional domain of ARHGAP24 in PKM2 protein stability and ubiquitination, we transfected expression vectors encoding FL-ARH-HA or deletion mutant (ARH-ΔCD-HA) into HCCLM3 cells. Results showed that mutant ARHGAP24 failed to induce ubiquitination of PKM2 as the WT-ARHGAP24 did (**Figure 8E**). Notably, PKM2 and WWP1 proteins in HCCLM3 cells could not be immunoprecipitated with ARHGAP24 when the C-terminal domain of ARHGAP24 was deleted (**Figure 8F**). Furthermore, C-terminal domain-deleted ARHGAP24 also failed to induce ubiquitination of PKM2 (**Figure S8C**). These findings consistently suggested that ARHGAP24 serves as a scaffolding protein that recruits WWP1 to PKM2 via its C-terminal. We further transfected expression vectors encoding PKM2-FL-FLAG, WWP1-FL-His and different deletion mutants of ARHGAP24, including ARH-FL-HA, M1 (amino acids (aa) 1 - 630), M2 (aa 1 - 530), M3 (aa 1 - 430) and M4 (aa 1 - 330) into HEK293T cells, followed by IP and western blot assays. The results showed that the C-terminal fragments 329 - 430 aa and 631 - 748 aa of ARHGAP24 bound directly to WWP1 and PKM2, respectively (**Figure 8G**). A schematic diagram of the mechanism of ARHGAP24 in HCC progression (**Figure 8H**) and the structural domains of ARHGAP24 interacting with WWP1 and PKM2 are shown in **Figure 8I**.

## Discussion

Abnormalities in Rho GTPase activation have major consequences for cancer cell proliferation and metastasis 22-23. RhoGAPs have been shown to inhibit the activation of Rho GTPase with an inactive GDP-bound state 6-7. RhoGAPs thus inactivate Rho GTPases (Rac1, CDC42) and have generally been presumed to act as tumor suppressors. However, the tumor suppressor roles of RhoGAPs have generally been reported in tumors other than HCC. For example, low ARHGAP30 expression promoted the proliferation and migration of colorectal carcinoma cells 18. Yagi found that ARAP3 expression inhibited cancer invasiveness by modulating cell adhesion and motility 24. Knockdown of ARHGAP15 resulted in activation of PAK1/2 and indirectly promoted Rac activation, thereby enhancing cancer-promoting signal transduction 25. Other RhoGAPs, such as ARHGAP4, ARHGAP6, ARHGAP9, ARHGAP12 and ARHGAP25, have also demonstrated tumor suppressor roles in different types of tumors 26-30. However, no study has systematically investigated the clinical significance of RhoGAPs and analyzed the functions of significant molecules in HCC. DLC1 and ARHGAP9 were reported to be tumor suppressors inhibiting HCC progression 28, 31. In the current study, we investigated the prognostic value of 64 RhoGAPs using TCGA and GEO databases, and showed that ARHGAP24 was significantly correlated with tumor progression. We also confirmed that ARHGAP24 was an independent indicator for TTR and OS in HCC patients. Importantly, low ARHGAP24 expression could help clinicians to identify HCC patients at high risk of recurrence, such as patients with alpha-fetoprotein < 400 µg/µL and early-stage tumors.

Previous studies reported that ARHGAP24 was a Rac-specific Rho-GTPase-activating protein that could inhibit cell morphology, migration and invasion 32-33. In the current study, ARHGAP24 exhibited different inhibitory efficiencies on RAC1 activity in different HCC cell lines. ARHGAP24 failed to decrease GTP-bound Rac1 levels in HCCLM3 cells, while ARHGAP24 overexpression could result in drastic inhibition of Rac1 activities in Huh7 cells, and silencing ARHGAP24 led to increased Rac1 activity in Li-7 cells. The inhibitory efficiency of ARHGAP24 on Rac1 thus varies among different HCC cell lines. Muller et al. recently performed fluorescence resonance energy transfer-based RhoGAP activity assays and revealed that ARHGAP24 had weak inhibitory efficiency against activated Rac1. Meanwhile, other RhoGAPs and guanine nucleotide exchange factors (RhoGEFs) were shown to have high substrate specificity and catalytic activities 8, in accordance with the current results showing dramatic differences in the inhibitory function of ARHGAP24 on Rac1 signaling among different cell lines. We speculated that this phenomenon might be attributed to the distinct intracellular environment. Previous studies showed that Rac1 signaling was typically regulated by RhoGEFs, RhoGAPs and guanine nucleotide dissociation inhibitors (RhoGDIs), and balancing the Rho signaling responses required coordination among all these factors 7-8. For instance, Feng et al. found that RASAL2 promoted small GTPase Rac1 signaling, which could bind and antagonize the Rac1-GAP protein ARHGAP24 in breast cancer 14. Interestingly, the highly metastatic HCCLM3 HCC cell line was reported to show high RhoGEF expression (DOCK1, IQGAP1) 34-35, which might activate Rac1 and maintain GTP-bound Rac1 at a high level regardless of *ARHGAP24* overexpression. On the other hand, modulation of ARHGAP24 expression might break the intracellular balance among RhoGEF, RhoGAP and RhoGDIs in Huh7 and Li-7 cells, resulting in alteration of Rac1 activity.

In fact, overexpression of ARHGAP24 could still result in dramatic inhibition of cell proliferation and invasion of HCCLM3 cells, regardless of the alteration in Rac1 activity (i.e., overexpression of ARHGAP24 could led to decreased proliferation and invasion but have no influence on GTP-Rac1 or GTP-CDC42 levels). Rac1 inactivation resulting from ARHGAP24 overexpression led to less nuclear accumulation of β-catenin in Huh7 cells, which might also contribute to the suppression of invasiveness of HCC cells. However, the present study found that forced expression of a mutant ARHGAP (Q158R) in Huh7 cells, which failed to inactivate Rac1 activity, had similar inhibitory effects on the proliferation, migration and invasion capacities of HCC cells without impairing Rac1 activity, as well as on the nuclear accumulation of β-catenin, a process regulated by Rac1 and considered to be crucial for β-catenin signaling activation. This lack of an association between the biological function and inhibition potentials of Rac1 signaling suggest that the main function of ARHGAP24 was not dependent on its enzymatic activity, and thus did not rely on its role in regulating Rac1 signaling. RhoGAPs have multiple domains. One RhoGAP domain contains catalytic arginine and thus maintains the GDP activation of Rho proteins, as the major mechanism for regulating cancer cell migration and invasion 8. However, other domains with unknown structures and functions may also be responsible for cell biological functions. Members of RhoGAPs could work as a scaffold to facilitate interactions between other proteins during tumor development, in an enzyme-independent manner. Wang et al. showed that ARHGAP30 promoted p53 acetylation and function independent of RhoGAP activity 18, while Yang et al. found that DLC1 interactions with S100A10 did not affect its RhoGAP activity 36. We therefore reasoned that ARHGAP24 might also exert its suppressive function as a scaffold.

In our study, LC/MS revealed PKM2 as a new binding partner of ARHGAP24 and showed that it could recruit WWP1, an E3 ligase, to promote ubiquitination as well as proteasomal degradation of PKM2 in HCC cells, even in Rac1 signaling-activated HCC cell lines. Notably, our results further revealed that the C-terminal of ARHGAP24 (fragments 329 - 430 and 631 - 748 aa), rather than the RhoGAP domain (135 - 330 aa), bound directly to WWP1 and PKM2. These findings consistently suggest that ARHGAP24 serves as a scaffolding protein that recruits WWP1 to PKM2 via its C-terminal. As reported previously, Rac1 activated β-catenin signaling mainly by facilitating its nuclear accumulation 37-38. However, the transcription activity of β-catenin was controlled by PKM2, indicating that Rac1 worked as an upstream regulator for β-catenin signaling, while PKM2 played a more elemental role and acted as a more crucial hub for β-catenin activation than Rac1. Knockdown of PKM2 accordingly resulted in a significant decrease in invasiveness among HCCLM3 cells in which Rac1 was highly activated. Critically, downregulation of PKM2 also abolished the promotional effects of *ARHGAP24* knockdown on β-catenin transcription activities and invasive potential in Rac1-activated HCC cells without suppression of Rac1 activity (*ARHGAP24*-knockdown Li-7 cells). Yang et al. accordingly revealed that PKM2 regulated β-catenin transactivation upon epidermal growth factor receptor activation, and PKM2 depletion significantly inhibited the binding of β-catenin to the promoter region of *CCND1* and *MYC* 20-21. Our data thus identified a novel role for ARHGAP24 in restraining PKM2 abundance by serving as a scaffold in HCC, independent of Rac1 activation.

PKM2 has been found to be highly expressed in various cancers 39. It can serve as a rate-limiting enzyme of cellular glycolysis or a transcriptional coactivator to promote cancer cell proliferation and invasion 40-41. Exploration of the underlying mechanisms responsible for the high expression of PKM2 will therefore provide new insights into HCC therapy. Ubiquitination modification was recently determined to be the core mechanism regulating intracellular protein stability, which is closely related to the expression of PKM2 42-43. Additionally, phosphorylation of PKM2 at Thr328, Thr454 and Tyr105 also relies on ubiquitination modification to maintain the stability of PKM2 44-46. However, the critical E3 ligases that directly mediate the degradation of PKM2 are rarely reported. Chen et al. found that E3 ligase ZFP91 promoted the ubiquitination of hnRNPA1 and proteasomal degradation, thereby resulting in PKM2 splicing 43. It has also been shown that PKM2 protein stability is regulated by Parkin, TRIM58 and CHIP E3 ligases 47-48. In our study, co-IP together with MS revealed that WWP1 was a novel E3 ligase that directly degraded PKM2. These results suggest that WWP1 could identify a novel substrate, PKM2, in high ARHGAP24-expressing cells and degrade it to regulate cell proliferation and invasion.

Several oncogenes related to the progression and poor prognosis of tumors were recently identified as suppressor genes, including *MYH9*, *USP9X* and *PHKB* 49-51. Additionally, canonical tumor suppressors, such as *TP53* and *PTEN*, have been found to promote carcinogenesis 50, 52. *WWP1* has been implicated as an oncogene in breast, prostate and liver cancer 53-55, and has been identified as a physical PTEN interactor, inducing polyubiquitination of PTEN to suppress its dimerization and membrane recruitment and unleash its tumor suppressive activity 56. ARID5a was also found to be a substrate of WWP1, and degradation of ARID5a resulted in the amplification of interleukin-6 expression, thereby inducing further inflammation 57. However, we unexpectedly found that *WWP1* had a tumor suppressor role. When ARHGAP24 is highly expressed in cancer cells, it may recruit WWP1 to form protein complexes and then promote PKM2 degradation. Taken together, our findings suggest that *WWP1* can serve as an oncogene or a tumor suppressor, depending on its interactions with different substrates. Notably, the tumor suppressor role of *WWP1* in HCC was mediated by *ARHGAP24* expression.

## Conclusions

This study was the first to identify ARHGAP24 as an independent prognostic indicator for HCC. Functional experiments revealed that it inhibits HCC progression and metastasis independent of RhoGAP activity, but attenuates β-catenin transactivation upon PKM2 degradation. Importantly, we identified a novel E3 ligase, WWP1, that can be recruited by the C-terminal of ARHGAP24 and subsequently induce proteasomal degradation of PKM2. These findings establish a novel function of RhoGAPs in HCC and provide a promising therapeutic target for HCC.

## Supplementary Material

Supplementary methods, figures and tables.

## Abbreviations

HCC: Hepatocellular carcinoma
RhoGAPs: Rho-GTPase activating proteins
ARHGAP24: Rho GTPase activating protein 24
PKM2: Pyruvate kinase M2
WWP1: WW Domain Containing E3 Ubiquitin Protein Ligase 1
TCGA: The Cancer Genome Atlas
GEO: Gene Expression Omnibus databases
CPTAC: clinical proteomic tumor analysis consortium
qRT-PCR: Quantitative reverse transcription-polymerase chain reaction
IHC: immunohistochemistry
LC-MS/MS: liquid chromatography-tandem mass spectrometry
CO-IP: co-immunoprecipitation
TTR: Time to tumor recurrence
OS: Overall survival
PFS: Progression-free survival
RFS: Relapse-free survival
DSS: Disease-specific survival
HR: Hazard ratio
WB: Western blotting
AFP: Alpha-fetoprotein
MVI: Microvascular invasion
PCNA: Proliferating cell nuclear antigen
CDK2: Cyclin-dependent kinase 2
EMT: Epithelial-mesenchymal transition
MMP-9: Matrix metalloproteinase-9
DEGs: Differentially expressed genes
GSEA: Gene set enrichment analysis
IP: Immunoprecipitation
CHX: Cycloheximide
RhoGEFs: Guanine nucleotide exchange factors
RhoGDIs: Guanine nucleotide dissociation inhibitors
MYC: MYC Proto-Oncogene
CCND1: Cyclin D1
Rac1: Rac Family Small GTPase 1
DOCK1: Dedicator Of Cytokinesis 1
IQGAP1: IQ Motif Containing GTPase Activating Protein 1
DLC1: DLC1 Rho GTPase Activating Protein
TRIM58: Tripartite Motif Containing 58
ZFP91: ZFP91 Zinc Finger Protein, Atypical E3 Ubiquitin Ligase
PHKB: Phosphorylase Kinase Regulatory Subunit Beta
PTEN: Phosphatase And Tensin Homolog
