# Supplementary material for: ARHGAP24 represses β-catenin transactivation-induced invasiveness in hepatocellular carcinoma mainly by acting as a GTPase-independent scaffold
Source: Theranostics. 2022 Aug 21;12(14):6189–206. doi: 10.7150/thno.72134 (PMC9475462; doi:10.7150/thno.72134)
Supplement: Supplementary file 1 — Supplementary methods, figures and tables. [file thnov12p6189s1.pdf]

## Title page

ARHGAP24 represses β-catenin transactivation-induced invasiveness in hepatocellular carcinoma mainly by acting as a GTPase-independent scaffold

Wenjing Yang[1*], Beili Wang[1,3,4*], Qian Yu[1,5*], Te Liu[6], Tong Li[1], Tongtong Tian[1], Anli Jin[1], Lin Ding[1], Wei Chen[1], Hao Wang[1], Jingrong Xian[1], Baishen Pan[1,3], Jian Zhou[2,3], Jia Fan[2,3], Xinrong Yang[2,3#], Wei Guo[1,3,4,5#]

[1]Department of Laboratory Medicine, Zhongshan Hospital, Fudan University, Shanghai 200032, P. R. China.

[2]Department of Liver Surgery & Transplantation, Liver Cancer Institute, Zhongshan Hospital, Fudan University; Key Laboratory of Carcinogenesis and Cancer Invasion, Ministry of Education, Shanghai 200032, P. R. China.

[3]Cancer Center, Zhongshan Hospital, Fudan University, Shanghai 200032, P. R. China.

[4]Department of Laboratory Medicine, Xiamen Branch, Zhongshan Hospital, Fudan University, Xiamen 361015, P. R. China.

[5]Department of Laboratory Medicine, Wusong Branch, Zhongshan Hospital, Fudan University, Shanghai 200940, P. R. China.

[6]Shanghai Geriatric Institute of Chinese Medicine, Shanghai University of Traditional Chinese Medicine, Shanghai 200031, P. R. China.

*These authors contributed equally.

**Contact information**

Wei Guo (Corresponding author)

Department of Laboratory Medicine, Zhongshan Hospital, Fudan University, Shanghai 200032, China.

Tel & Fax: +86-21-64041990-2376

Email: guo.wei@zs-hospital.sh.cn

Or to

Xin-Rong Yang (Corresponding author)

Department of Liver Surgery, Liver Cancer Institute, Zhongshan Hospital, Fudan University, 136 Yi Xue Yuan Road, Shanghai 200032, P. R. China.

Tel. & Fax: +86-21-64037181.

E-mail: yang.xinrong@zs-hospital.sh.cn

## Supplementary Figures

**Supplementary figure 1. Low ARHGAP24 expression indicates the poorer prognosis of HCC patients. A.** Kaplan-Meier analysis of the overall survival (OS) in HCC patients stratified by ARHGAP24 expression according to K-M plotter dataset. **B.** Kaplan-Meier analysis of the relapse-free survival (RFS) in HCC patients stratified by ARHGAP24 expression according to K-M plotter dataset. **C.** Kaplan-Meier analysis of the progression-free survival (PFS) in HCC patients stratified by ARHGAP24 expression according to K-M plotter dataset. **D.** Kaplan-Meier analysis of the disease-specific survival (DSS) in HCC patients stratified by ARHGAP24 expression according to K-M plotter dataset.

**Supplementary figure 2. ARHGAP24 expression could predict the prognosis of HCC patients with high risk of recurrence. A.** Kaplan-Meier analysis of progression-free survival (PFS) of HCC patients with tumor size < 5 cm, with single tumor number, and with early tumor differentiation. **B.** Kaplan-Meier analysis of progression-free survival (PFS) of HCC patients with early tumor stage (Chinese liver tumor stage), without satellite lesions and without microvascular invasion. **C.** Univariate cox proportional regression analysis of factors associated with HCC tumor recurrence.

**Supplementary figure 3. Kaplan-Meier analysis for OS in other tumors. A.**

Kaplan-Meier analysis of the OS of kidney renal clear cell carcinoma in ARHGAP24 high expression group and ARHGAP24 low expression group. **B.** Kaplan-Meier analysis of the OS of lung adenocarcinoma in ARHGAP24 high expression group and ARHGAP24 low expression group. **C.** Kaplan-Meier analysis of the OS of pancreatic ductal adenocarcinoma in ARHGAP24 high expression group and ARHGAP24 low expression group. **D.** Kaplan-Meier analysis of the OS of kidney renal papillary cell carcinoma in ARHGAP24 high expression group and ARHGAP24 low expression group. **E.** Kaplan-Meier analysis of the OS of lung squamous cell carcinoma in ARHGAP24 high expression group and ARHGAP24 low expression group.

**Supplementary figure 4. ARHGAP24 overexpression inhibits huh7 cell proliferation. A.** Cell proliferation-related molecules were detected by WB assays in ARHGAP24 overexpressed-Huh7 cells. **B.** Effects of ARHGAP24 overexpression on huh7 cell proliferation using colony formation assays. **C.** Effects of ARHGAP24 overexpression on huh7 cell proliferation using CCK8 assays. **D.** Effects of ARHGAP24 knockdown on Li-7 cell cycle using flow cytometry. **E.** Effects of ARHGAP24 overexpression on Huh7 cell cycle using flow cytometry. **F.** Effects of ARHGAP24 overexpression on HCCLM3 cell cycle using flow cytometry. **G.** Effects of ARHGAP24 overexpression on Huh7 cell apoptosis using flow cytometry.

**Supplementary figure 5. ARHGAP24 inhibits cell invasiveness via inactivating wnt/β-catenin signaling pathway. A.** Effects of ARHGAP24 overexpression on Huh7 cell migration and invasion using Transwell assays. **B.** Effects of ARHGAP24 overexpression on Huh7 cell migration using scratch wound healing assays. **C.** qRT-PCR analysis of EMT-related markers in ARHGAP24 overexpressed Huh7 cells. **D.** Protein expression of EMT-related markers in ARHGAP24 overexpressed Huh7 cells. **E.** Protein expression of downstream target genes of β-catenin in ARHGAP24 overexpression and knockdown cells. **F.** Effects of ICG-001 on HCCLM3 cell proliferation using CCK8 assays. **G.** Effects of ICG-001 on sh-ARH Li-7 cell proliferation using CCK8 assays.

**Supplementary figure 6. ARHGAP24 inhibits cell invasiveness independent of Rho GTPase activity. A.** Q158R plasmids were successfully constructed and confirmed by sanger sequence analysis. **B.** Protein expressions of EMT-related markers in HCCLM3 cells transfected with the indicated plasmids. **C.** Protein expressions of EMT-related markers in Huh7 cells transfected with the indicated plasmids.

**Supplementary figure 7. ARHGAP24 inhibits HCC progression by degradation of PKM2. A.** PKM2 was successfully silenced in ARHGAP24 knockdown cells. **B.** Protein expressions of EMT-related markers were detected

by WB assays. **C.** Silenced PKM2 have no effects on increased Rho GTPase activity caused by knockdown ARHGAP24 in Li-7 cells. **D.** Effects of PKM2 silencing on HCCLM3 cell proliferation using CCK8 assays. **E.** Effects of PKM2 silencing on sh-ARH Li-7 cell proliferation using CCK8 assays.

**Supplementary figure 8. ARHGAP24 recruits WWP1 to degrade PKM2. A.** IP and WB analyses of PKM2, WWP1 and ARHGAP24 protein expression in HCCLM3 and MHCC97H cells transfected with the numbers of ARHGAP24-HA plasmids. **B.** Representative pictures of the binding domain of the ARHGAP24 and PKM2 structure. **C.** IP and WB analyses and Ubiquitination assays of PKM2 in the presence or absence of ARHGAP24 C-terminal domain.

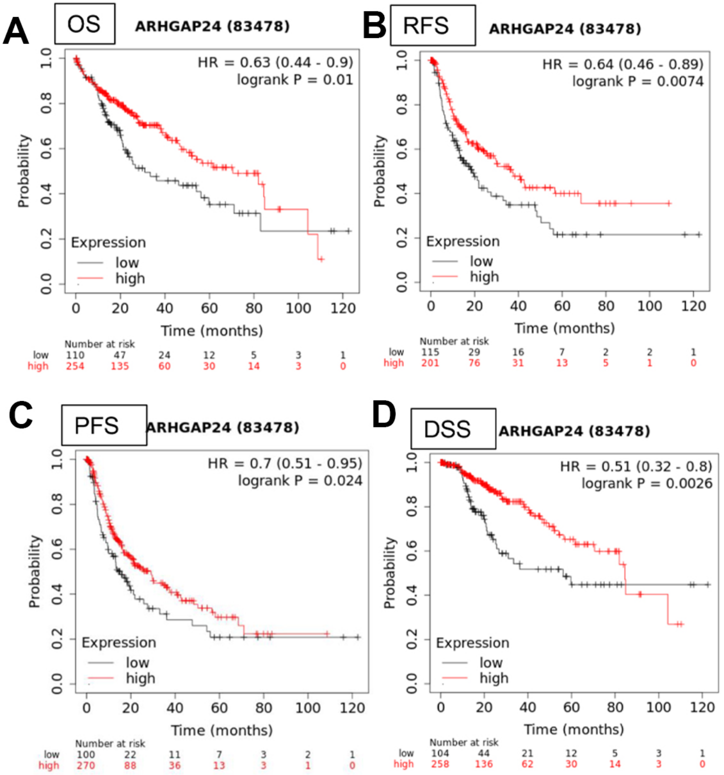
A
OS
ARHGAP24 (83478)
HR = 0.63 (0.44 - 0.9)
logrank P = 0.01
Probability
Time (months)
Expression
low
high
Number at risk
low 110 47 24 12 5 3 1
high 254 135 60 30 14 3 0
B
RFS
ARHGAP24 (83478)
HR = 0.64 (0.46 - 0.89)
logrank P = 0.0074
Probability
Time (months)
Expression
low
high
Number at risk
low 115 29 16 7 2 2 1
high 201 76 31 13 5 1 0
C
PFS
ARHGAP24 (83478)
HR = 0.7 (0.51 - 0.95)
logrank P = 0.024
Probability
Time (months)
Expression
low
high
Number at risk
low 100 22 11 7 3 2 1
high 270 88 36 13 3 1 0
D
DSS
ARHGAP24 (83478)
HR = 0.51 (0.32 - 0.8)
logrank P = 0.0026
Probability
Time (months)
Expression
low
high
Number at risk
low 104 44 21 12 5 3 1
high 258 136 62 30 14 3 0

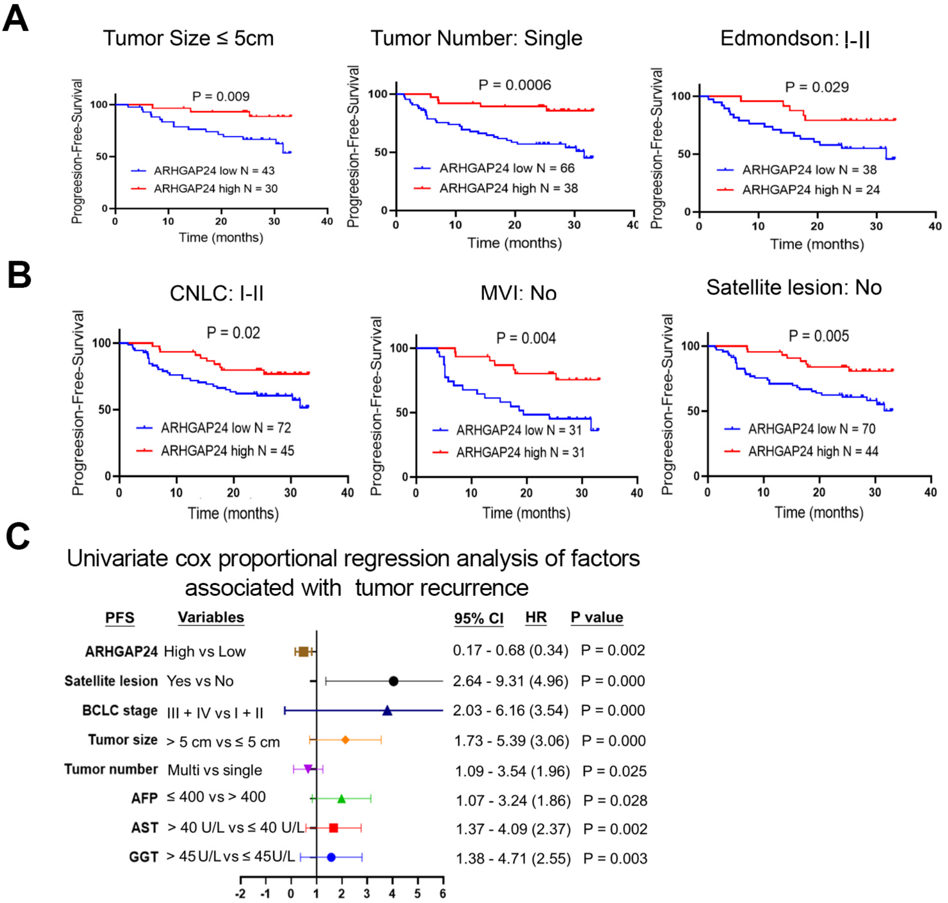
A
Tumor Size ≤ 5cm
P = 0.009
ARHGAP24 low N = 43
ARHGAP24 high N = 30
Tumor Number: Single
P = 0.0006
ARHGAP24 low N = 66
ARHGAP24 high N = 38
Edmondson: I-II
P = 0.029
ARHGAP24 low N = 38
ARHGAP24 high N = 24
Progreesion-Free-Survival
Time (months)
B
CNLC: I-II
P = 0.02
ARHGAP24 low N = 72
ARHGAP24 high N = 45
MVI: No
P = 0.004
ARHGAP24 low N = 31
ARHGAP24 high N = 31
Satellite lesion: No
P = 0.005
ARHGAP24 low N = 70
ARHGAP24 high N = 44
C
Univariate cox proportional regression analysis of factors associated with tumor recurrence
PFS
Variables
95% CI
HR
P value
ARHGAP24 High vs Low 0.17 - 0.68 (0.34) P = 0.002
Satellite lesion Yes vs No 2.64 - 9.31 (4.96) P = 0.000
BCLC stage III + IV vs I + II 2.03 - 6.16 (3.54) P = 0.000
Tumor size > 5 cm vs ≤ 5 cm 1.73 - 5.39 (3.06) P = 0.000
Tumor number Multi vs single 1.09 - 3.54 (1.96) P = 0.025
AFP ≤ 400 vs > 400 1.07 - 3.24 (1.86) P = 0.028
AST > 40 U/L vs ≤ 40 U/L 1.37 - 4.09 (2.37) P = 0.002
GGT > 45 U/L vs ≤ 45U/L 1.38 - 4.71 (2.55) P = 0.003

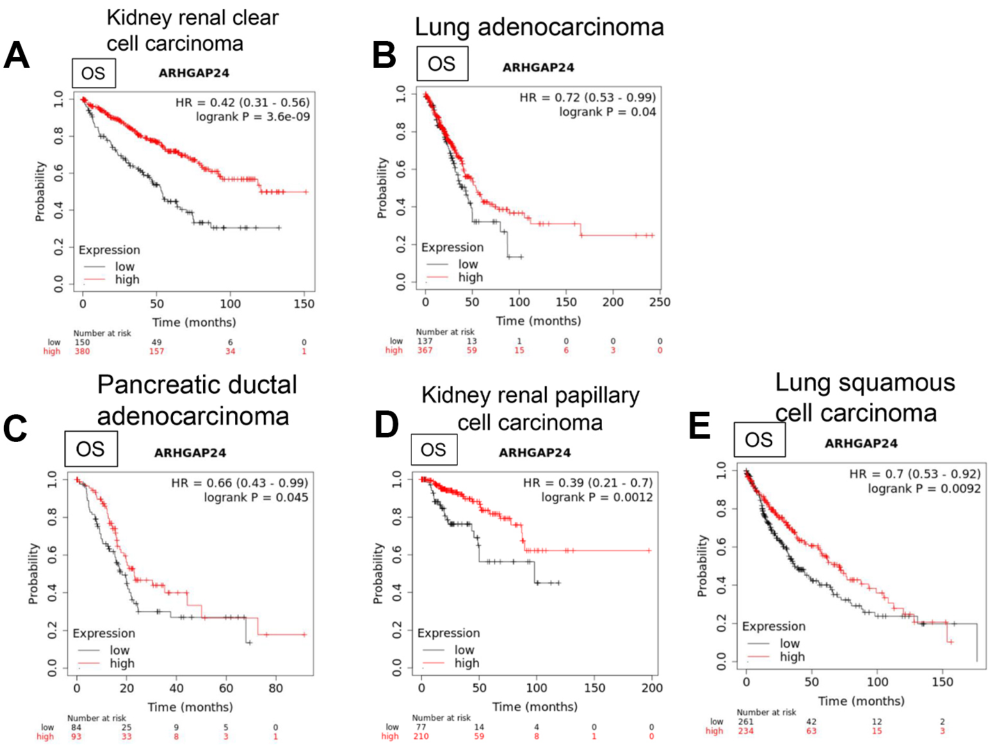

A
Kidney renal clear cell carcinoma
OS
ARHGAP24
HR = 0.42 (0.31 - 0.56)
logrank P = 3.6e-09
Probability
Time (months)
Expression
low
high
Number at risk
low 150 49 6 0
high 380 157 34 1
B
Lung adenocarcinoma
OS
ARHGAP24
HR = 0.72 (0.53 - 0.99)
logrank P = 0.04
Number at risk
low 137 13 1 0 0 0
high 367 59 15 6 3 0
C
Pancreatic ductal adenocarcinoma
OS
ARHGAP24
HR = 0.66 (0.43 - 0.99)
logrank P = 0.045
Number at risk
low 84 25 9 5 0
high 93 33 8 3 1
D
Kidney renal papillary cell carcinoma
OS
ARHGAP24
HR = 0.39 (0.21 - 0.7)
logrank P = 0.0012
Number at risk
low 77 14 4 0 0
high 210 59 8 1 0
E
Lung squamous cell carcinoma
OS
ARHGAP24
HR = 0.7 (0.53 - 0.92)
logrank P = 0.0092
Number at risk
low 261 42 12 2
high 234 63 15 3

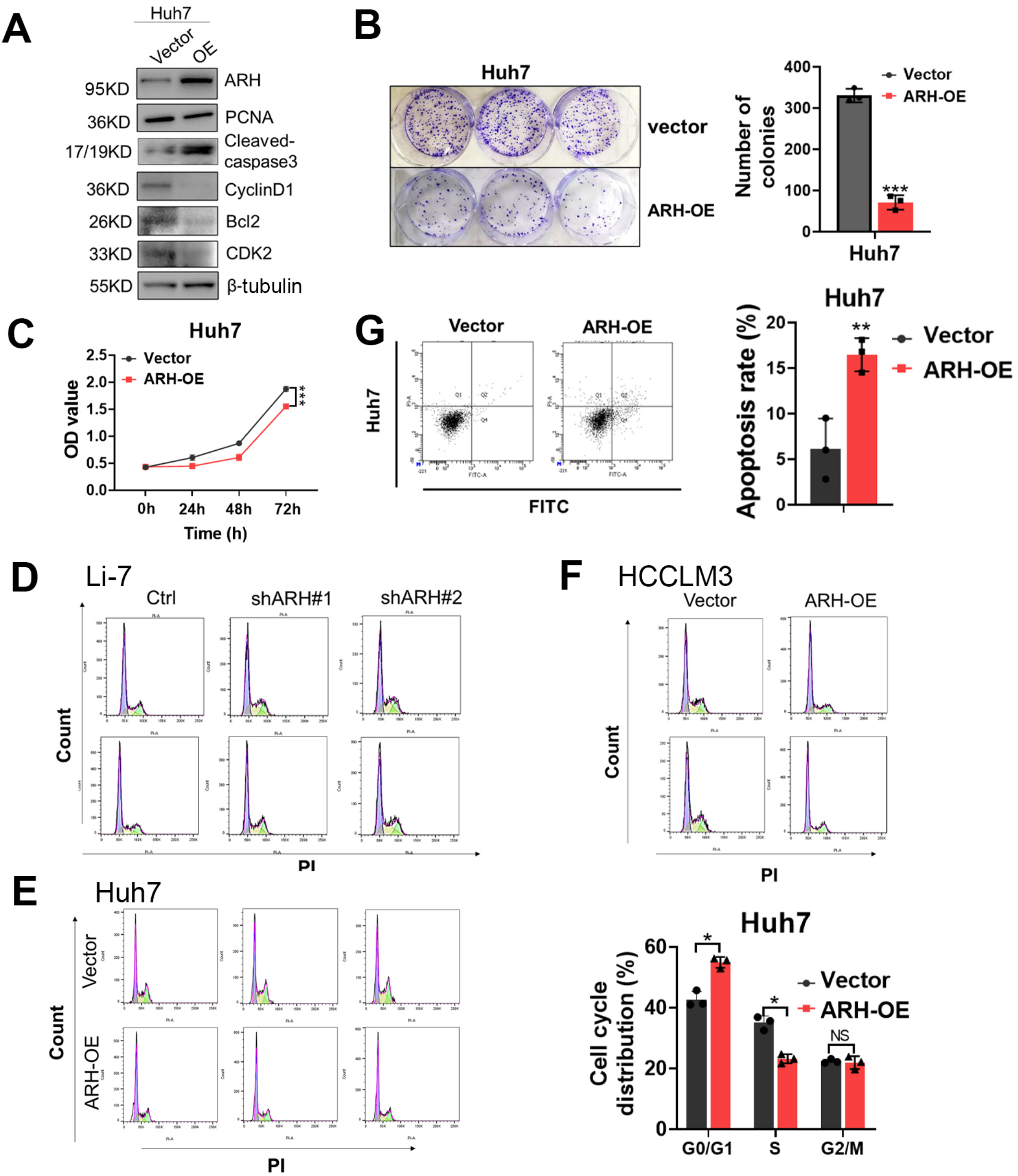
A
Huh7
Vector
OE
95KD
ARH
36KD
PCNA
17/19KD
Cleaved-caspase3
36KD
CyclinD1
26KD
Bcl2
33KD
CDK2
55KD
β-tubulin
B
Huh7
vector
ARH-OE
Number of colonies
Vector
ARH-OE
***
C
Huh7
OD value
Vector
ARH-OE
***
0h
24h
48h
72h
Time (h)
G
Vector
ARH-OE
Huh7
FITC
Apoptosis rate (%)
**
D
Li-7
Ctrl
shARH#1
shARH#2
Count
PI
F
HCCLM3
Vector
ARH-OE
Count
PI
E
Huh7
Vector
ARH-OE
Count
PI
Huh7
Cell cycle distribution (%)
Vector
ARH-OE
*
*
NS
G0/G1
S
G2/M

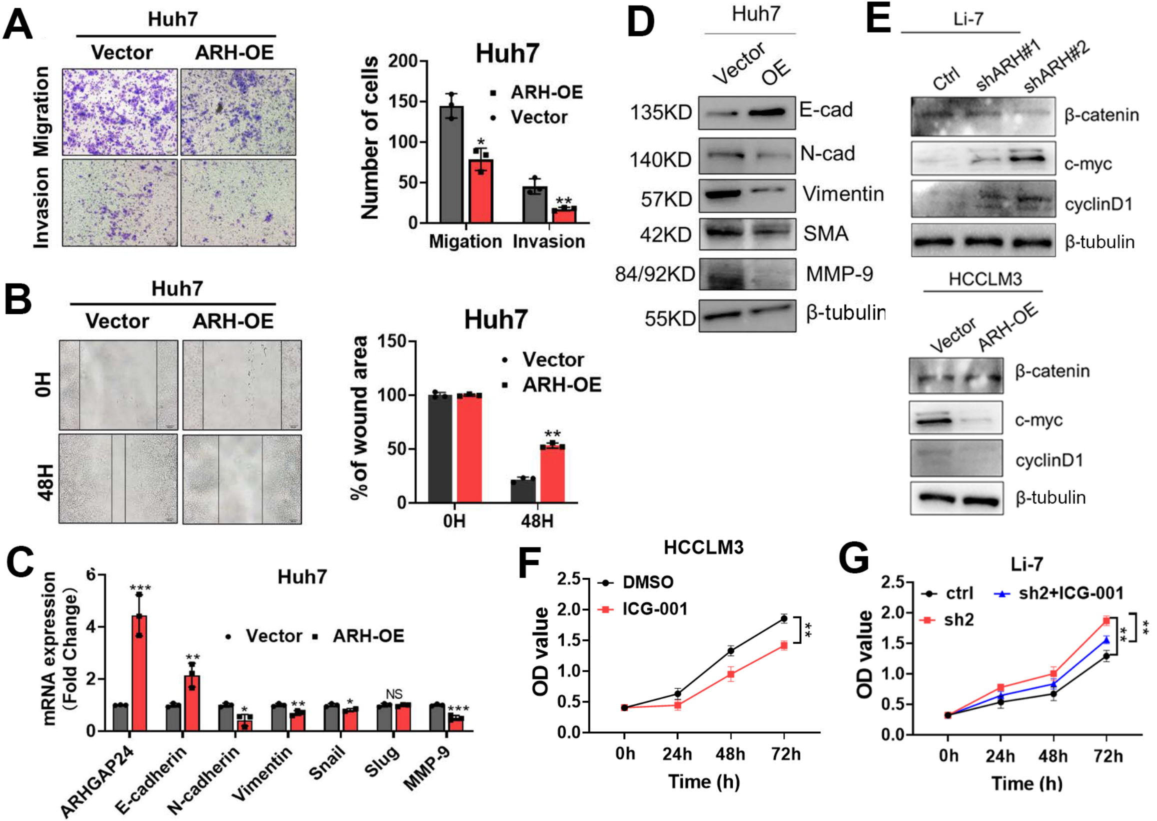

A
Huh7
Vector
ARH-OE
Migration
Invasion
Huh7
Number of cells
ARH-OE
Vector
Migation
Invasion
B
Huh7
Vector
ARH-OE
0H
48H
Huh7
%of wound area
Vector
ARH-OE
0H
48H
C
Huh7
mRNA expression (Fold Change)
Vector
ARH-OE
NS
ARHGAP24
E-cadherin
N-cadherin
Vimentin
Snail
Slug
MMP-9
D
Huh7
Vector
OE
135KD
E-cad
140KD
N-cad
57KD
Vimentin
42KD
SMA
84/92KD
MMP-9
55KD
β-tubulin
E
Li-7
Ctrl
shARH#1
shARH#2
β-catenin
c-myc
cyclinD1
β-tubulin
HCCLM3
Vector
ARH-OE
β-catenin
c-myc
cyclinD1
β-tubulin
F
HCCLM3
DMSO
ICG-001
OD value
Time (h)
G
Li-7
ctrl
sh2
sh2+ICG-001
OD value
Time (h)

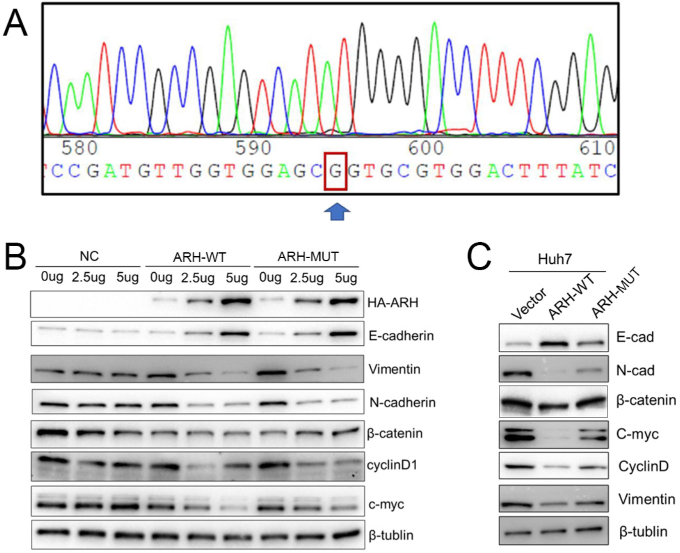
A
580
590
600
610
TCCGATGTTGGTGGAGCGGTGCGTGGACTTTATC
B
NC
ARH-WT
ARH-MUT
0ug 2.5ug 5ug 0ug 2.5ug 5ug 0ug 2.5ug 5ug
HA-ARH
E-cadherin
Vimentin
N-cadherin
β-catenin
cyclinD1
c-myc
β-tublin
C
Huh7
Vector
ARH-WT
ARH-MUT
E-cad
N-cad
β-catenin
C-myc
CyclinD
Vimentin
β-tublin

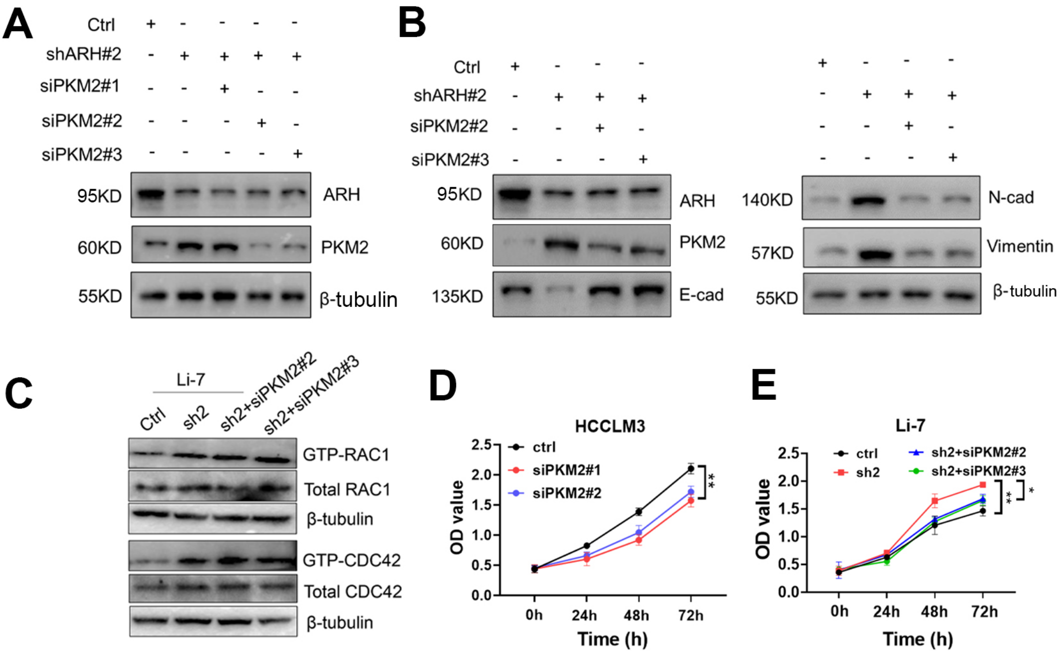
A
Ctrl
shARH#2
siPKM2#1
siPKM2#2
siPKM2#3
95KD
ARH
60KD
PKM2
55KD
β-tubulin
B
Ctrl
shARH#2
siPKM2#2
siPKM2#3
95KD
ARH
60KD
PKM2
135KD
E-cad
140KD
N-cad
57KD
Vimentin
55KD
β-tubulin
C
Li-7
Ctrl
sh2
sh2+siPKM2#2
sh2+siPKM2#3
GTP-RAC1
Total RAC1
β-tubulin
GTP-CDC42
Total CDC42
β-tubulin
D
HCCLM3
ctrl
siPKM2#1
siPKM2#2
OD value
Time (h)
0h
24h
48h
72h
E
Li-7
ctrl
sh2
sh2+siPKM2#2
sh2+siPKM2#3
OD value
Time (h)
0h
24h
48h
72h

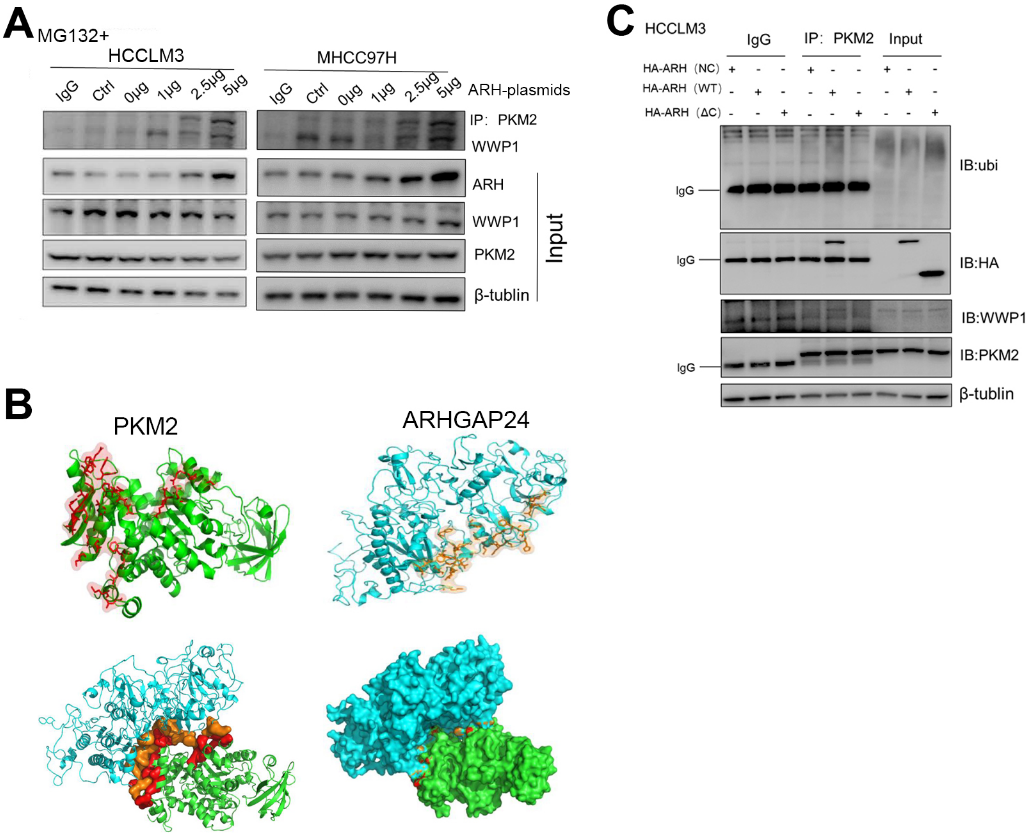
A
MG132+
HCCLM3
MHCC97H
IgG
Ctrl
0μg
1μg
2.5μg
5μg
ARH-plasmids
IP: PKM2
WWP1
ARH
WWP1
PKM2
β-tublin
Input
B
PKM2
ARHGAP24
C
HCCLM3
IgG
IP: PKM2
Input
HA-ARH (NC)
HA-ARH (WT)
HA-ARH (ΔC)
IB:ubi
IB:HA
IB:WWP1
IB:PKM2
β-tublin

## Supplementary Tables

**Supplementary Table 1.** List of potential interactors of ARHGAP24 identified by IP-MS.

**Supplementary Table 2.** The correlations between ARHGAP24 expression and clinicopathological characteristics in HCC.

**Supplementary Table 3.** Univariate cox proportional regression analysis of factors associated with recurrence and overall survival.

| Number | Accession | -10lgP | Avg. Mass | Description |
|---|---|---|---|---|
| 1 | sp\|Q8N264\|RHG24_HUMAN | 476.95 | 84258 | Rho GTPase-activating protein 24 OS=Homo sapiens OX=9606 GN=ARHGAP24 PE=1 SV=2 |
| 2 | tr\|D6RCP5\|D6RCP5_HUMAN | 447.89 | 69481 | Rho GTPase-activating protein 24 (Fragment) OS=Homo sapiens OX=9606 GN=ARHGAP24 PE=1 SV=1 |
| 3 | sp\|P07900\|HS90A_HUMAN | 408.91 | 84660 | Heat shock protein HSP 90-alpha OS=Homo sapiens OX=9606 GN=HSP90AA1 PE=1 SV=5 |
| 4 | sp\|P04264\|K2C1_HUMAN | 405.95 | 66039 | Keratin type II cytoskeletal 1 OS=Homo sapiens OX=9606 GN=KRT1 PE=1 SV=6 |
| 5 | sp\|P08238\|HS90B_HUMAN | 397.88 | 83264 | Heat shock protein HSP 90-beta OS=Homo sapiens OX=9606 GN=HSP90AB1 PE=1 SV=4 |
| 6 | sp\|P14618\|KPYM_HUMAN | 388.63 | 57937 | Pyruvate kinase PKM OS=Homo sapiens OX=9606 GN=PKM PE=1 SV=4 |
| 7 | sp\|P35527\|K1C9_HUMAN | 378.05 | 62064 | Keratin type I cytoskeletal 9 OS=Homo sapiens OX=9606 GN=KRT9 PE=1 SV=3 |
| 8 | sp\|Q9H0M0\|WWP1_HUMAN | 334.02 | 105202 | NEDD4-like E3 ubiquitin-protein ligase WWP1 OS=Homo sapiens OX=9606 GN=WWP1 PE=1 SV=1 |
| 9 | sp\|P13639\|EF2_HUMAN | 326.51 | 95338 | Elongation factor 2 OS=Homo sapiens OX=9606 GN=EEF2 PE=1 SV=4 |
| 10 | sp\|P23246\|SFPQ_HUMAN | 321.83 | 76150 | Splicing factor proline- and glutamine-rich OS=Homo sapiens OX=9606 GN=SFPQ PE=1 SV=2 |
| 11 | sp\|P10809\|CH60_HUMAN | 320.54 | 61055 | 60 kDa heat shock protein mitochondrial OS=Homo sapiens OX=9606 GN=HSPD1 PE=1 SV=2 |
| 12 | sp\|P13645\|K1C10_HUMAN | 318.53 | 58827 | Keratin type I cytoskeletal 10 OS=Homo sapiens OX=9606 GN=KRT10 PE=1 SV=6 |
| 13 | sp\|P0DMV8\|HS71A_HUMAN | 304.36 | 70052 | Heat shock 70 kDa protein 1A OS=Homo sapiens OX=9606 GN=HSPA1A PE=1 SV=1 |
| 14 | sp\|P0DMV9\|HS71B_HUMAN | 304.36 | 70052 | Heat shock 70 kDa protein 1B OS=Homo sapiens OX=9606 GN=HSPA1B PE=1 SV=1 |
| 15 | tr\|A0A0G2JIW1\|A0A0G2JIW1_HUMAN | 304.36 | 70109 | Heat shock 70 kDa protein 1B OS=Homo sapiens OX=9606 GN=HSPA1B PE=1 SV=1 |
| 16 | sp\|P35908\|K22E_HUMAN | 303.27 | 65433 | Keratin type II cytoskeletal 2 epidermal OS=Homo sapiens OX=9606 GN=KRT2 PE=1 SV=2 |
| 17 | sp\|P27824\|CALX_HUMAN | 300.52 | 67568 | Calnexin OS=Homo sapiens OX=9606 GN=CANX PE=1 SV=2 |
| 18 | sp\|P09874\|PARP1_HUMAN | 298.49 | 113084 | Poly [ADP-ribose] polymerase 1 OS=Homo sapiens OX=9606 GN=PARP1 PE=1 SV=4 |
| 19 | sp\|P13010\|XRCC5_HUMAN | 283.79 | 82705 | X-ray repair cross-complementing protein 5 OS=Homo sapiens OX=9606 GN=XRCC5 PE=1 SV=3 |
| 20 | sp\|Q00839\|HNRPU_HUMAN | 280.32 | 90585 | Heterogeneous nuclear ribonucleoprotein U OS=Homo sapiens OX=9606 GN=HNRNPU PE=1 SV=6 |
| 21 | sp\|P55072\|TERA_HUMAN | 277.94 | 89322 | Transitional endoplasmic reticulum ATPase OS=Homo sapiens OX=9606 GN=VCP PE=1 SV=4 |
| 22 | sp\|Q9NR30\|DDX21_HUMAN | 276.45 | 87344 | Nucleolar RNA helicase 2 OS=Homo sapiens OX=9606 GN=DDX21 PE=1 SV=5 |
| 23 | tr\|B9A067\|B9A067_HUMAN | 275.06 | 78974 | MICOS complex subunit MIC60 OS=Homo sapiens OX=9606 GN=IMMT PE=1 SV=2 |
| 24 | sp\|Q16891\|MIC60_HUMAN | 275.06 | 83678 | MICOS complex subunit MIC60 OS=Homo sapiens OX=9606 GN=IMMT PE=1 SV=1 |
| 25 | sp\|P31948\|STIP1_HUMAN | 271.16 | 62639 | Stress-induced-phosphoprotein 1 OS=Homo sapiens OX=9606 GN=STIP1 PE=1 SV=1 |
| 26 | sp\|P33993\|MCM7_HUMAN | 270.96 | 81308 | DNA replication licensing factor MCM7 OS=Homo sapiens OX=9606 GN=MCM7 PE=1 SV=4 |
| 27 | sp\|O43143\|DHX15_HUMAN | 268 | 90933 | Pre-mRNA-splicing factor ATP-dependent RNA helicase DHX15 OS=Homo sapiens OX=9606 GN=DHX15 PE=1 SV=2 |
| 28 | sp\|Q01813\|PFKAP_HUMAN | 264.17 | 85596 | ATP-dependent 6-phosphofructokinase platelet type OS=Homo sapiens OX=9606 GN=PFKP PE=1 SV=2 |
| 29 | sp\|P22314\|UBA1_HUMAN | 262.3 | 117849 | Ubiquitin-like modifier-activating enzyme 1 OS=Homo sapiens OX=9606 GN=UBA1 PE=1 SV=3 |
| 30 | sp\|P11142\|HSP7C_HUMAN | 261.73 | 70898 | Heat shock cognate 71 kDa protein OS=Homo sapiens OX=9606 GN=HSPA8 PE=1 SV=1 |
| 31 | sp\|Q12906\|ILF3_HUMAN | 259.29 | 95339 | Interleukin enhancer-binding factor 3 OS=Homo sapiens OX=9606 GN=ILF3 PE=1 SV=3 |
| 32 | sp\|P49368\|TCPG_HUMAN | 258.11 | 60534 | T-complex protein 1 subunit gamma OS=Homo sapiens OX=9606 GN=CCT3 PE=1 SV=4 |
| 33 | sp\|P47897\|SYQ_HUMAN | 256.49 | 87799 | Glutamine--tRNA ligase OS=Homo sapiens OX=9606 GN=QARS PE=1 SV=1 |
| 34 | sp\|P38646\|GRP75_HUMAN | 256.08 | 73681 | Stress-70 protein mitochondrial OS=Homo sapiens OX=9606 GN=HSPA9 PE=1 SV=2 |
| 35 | sp\|P19338\|NUCL_HUMAN | 254.37 | 76615 | Nucleolin OS=Homo sapiens OX=9606 GN=NCL PE=1 SV=3 |
| 36 | sp\|P53396\|ACLY_HUMAN | 254.33 | 120839 | ATP-citrate synthase OS=Homo sapiens OX=9606 GN=ACLY PE=1 SV=3 |
| 37 | sp\|P33992\|MCM5_HUMAN | 252.29 | 82286 | DNA replication licensing factor MCM5 OS=Homo sapiens OX=9606 GN=MCM5 PE=1 SV=5 |
| 38 | sp\|O75534\|CSDE1_HUMAN | 251.09 | 88885 | Cold shock domain-containing protein E1 OS=Homo sapiens OX=9606 GN=CSDE1 PE=1 SV=2 |
| 39 | sp\|P13797\|PLST_HUMAN | 250.79 | 70811 | Plastin-3 OS=Homo sapiens OX=9606 GN=PLS3 PE=1 SV=4 |
| 40 | sp\|P61978\|HNRPK_HUMAN | 240.92 | 50976 | Heterogeneous nuclear ribonucleoprotein K OS=Homo sapiens OX=9606 GN=HNRNPK PE=1 SV=1 |
| 41 | sp\|Q14974\|IMB1_HUMAN | 240.54 | 97170 | Importin subunit beta-1 OS=Homo sapiens OX=9606 GN=KPNB1 PE=1 SV=2 |
| 42 | sp\|Q8N1F7\|NUP93_HUMAN | 240.08 | 93488 | Nuclear pore complex protein Nup93 OS=Homo sapiens OX=9606 GN=NUP93 PE=1 SV=2 |
| 43 | sp\|Q14697\|GANAB_HUMAN | 235.54 | 106874 | Neutral alpha-glucosidase AB OS=Homo sapiens OX=9606 GN=GANAB PE=1 SV=3 |
| 44 | sp\|P50990\|TCPQ_HUMAN | 230.63 | 59621 | T-complex protein 1 subunit theta OS=Homo sapiens OX=9606 GN=CCT8 PE=1 SV=4 |
| 45 | sp\|P29401\|TKT_HUMAN | 229.32 | 67878 | Transketolase OS=Homo sapiens OX=9606 GN=TKT PE=1 SV=3 |
| 46 | sp\|Q4VCS5\|AMOT_HUMAN | 228.51 | 118085 | Angiomotin OS=Homo sapiens OX=9606 GN=AMOT PE=1 SV=1 |
| 47 | sp\|Q99798\|ACON_HUMAN | 225.8 | 85425 | Aconitate hydratase mitochondrial OS=Homo sapiens OX=9606 GN=ACO2 PE=1 SV=2 |
| 48 | tr\|A2A274\|A2A274_HUMAN | 225.8 | 87820 | Aconitate hydratase mitochondrial OS=Homo sapiens OX=9606 GN=ACO2 PE=1 SV=1 |
| 49 | sp\|Q12788\|TBL3_HUMAN | 222.6 | 89035 | Transducin beta-like protein 3 OS=Homo sapiens OX=9606 GN=TBL3 PE=1 SV=2 |
| 50 | sp\|Q9UHB6\|LIMA1_HUMAN | 222.03 | 85226 | LIM domain and actin-binding protein 1 OS=Homo sapiens OX=9606 GN=LIMA1 PE=1 SV=1 |
| 51 | sp\|P02768\|ALBU_HUMAN | 221.41 | 69367 | Serum albumin OS=Homo sapiens OX=9606 GN=ALB PE=1 SV=2 |
| 52 | sp\|P14625\|ENPL_HUMAN | 219.92 | 92469 | Endoplasmin OS=Homo sapiens OX=9606 GN=HSP90B1 PE=1 SV=1 |
| 53 | tr\|C9J2Y9\|C9J2Y9_HUMAN | 219.8 | 133057 | DNA-directed RNA polymerase subunit beta OS=Homo sapiens OX=9606 GN=POLR2B PE=1 SV=2 |
| 54 | sp\|P30876\|RPB2_HUMAN | 219.8 | 133896 | DNA-directed RNA polymerase II subunit RPB2 OS=Homo sapiens OX=9606 GN=POLR2B PE=1 SV=1 |
| 55 | sp\|P31939\|PUR9_HUMAN | 217.52 | 64616 | Bifunctional purine biosynthesis protein PURH OS=Homo sapiens OX=9606 GN=ATIC PE=1 SV=3 |
| 56 | sp\|P78347\|GTF2I_HUMAN | 217.03 | 112416 | General transcription factor II-I OS=Homo sapiens OX=9606 GN=GTF2I PE=1 SV=2 |
| 57 | sp\|P40227\|TCPZ_HUMAN | 214.56 | 58024 | T-complex protein 1 subunit zeta OS=Homo sapiens OX=9606 GN=CCT6A PE=1 SV=3 |
| 58 | sp\|P23921\|RIR1_HUMAN | 210.97 | 90070 | Ribonucleoside-diphosphate reductase large subunit OS=Homo sapiens OX=9606 GN=RRM1 PE=1 SV=1 |
| 59 | sp\|P48643\|TCPE_HUMAN | 208.14 | 59671 | T-complex protein 1 subunit epsilon OS=Homo sapiens OX=9606 GN=CCT5 PE=1 SV=1 |
| 60 | sp\|P05023\|AT1A1_HUMAN | 205.4 | 112896 | Sodium/potassium-transporting ATPase subunit alpha-1 OS=Homo sapiens OX=9606 GN=ATP1A1 PE=1 SV=1 |
| 61 | sp\|P26639\|SYTC_HUMAN | 205.3 | 83435 | Threonine--tRNA ligase cytoplasmic OS=Homo sapiens OX=9606 GN=TARS PE=1 SV=3 |
| 62 | sp\|P34932\|HSP74_HUMAN | 203.65 | 94331 | Heat shock 70 kDa protein 4 OS=Homo sapiens OX=9606 GN=HSPA4 PE=1 SV=4 |
| 63 | tr\|A0A087WYC1\|A0A087WYC1_HUMAN | 203.65 | 78606 | Heat shock 70 kDa protein 4 OS=Homo sapiens OX=9606 GN=HSPA4 PE=1 SV=1 |
| 64 | tr\|J3KTA4\|J3KTA4_HUMAN | 200.66 | 69087 | Probable ATP-dependent RNA helicase DDX5 OS=Homo sapiens OX=9606 GN=DDX5 PE=1 SV=1 |
| 65 | sp\|P17844\|DDX5_HUMAN | 200.66 | 69148 | Probable ATP-dependent RNA helicase DDX5 OS=Homo sapiens OX=9606 GN=DDX5 PE=1 SV=1 |
| 66 | tr\|F1T0B3\|F1T0B3_HUMAN | 198.89 | 73915 | ATP-dependent RNA helicase DDX1 OS=Homo sapiens OX=9606 GN=DDX1 PE=1 SV=1 |
| 67 | tr\|A0A087X2G1\|A0A087X2G1_HUMAN | 198.89 | 73975 | ATP-dependent RNA helicase DDX1 OS=Homo sapiens OX=9606 GN=DDX1 PE=1 SV=1 |
| 68 | sp\|Q92499\|DDX1_HUMAN | 198.89 | 82432 | ATP-dependent RNA helicase DDX1 OS=Homo sapiens OX=9606 GN=DDX1 PE=1 SV=2 |
| 69 | tr\|A0A0A0MS51\|A0A0A0MS51_HUMAN | 194.07 | 82526 | Gelsolin OS=Homo sapiens OX=9606 GN=GSN PE=1 SV=1 |
| 70 | tr\|A0A0A0MT01\|A0A0A0MT01_HUMAN | 194.07 | 84745 | Gelsolin OS=Homo sapiens OX=9606 GN=GSN PE=1 SV=1 |
| 71 | sp\|P06396\|GELS_HUMAN | 194.07 | 85697 | Gelsolin OS=Homo sapiens OX=9606 GN=GSN PE=1 SV=1 |
| 72 | sp\|P08237\|PFKAM_HUMAN | 193.75 | 85183 | ATP-dependent 6-phosphofructokinase muscle type OS=Homo sapiens OX=9606 GN=PFKM PE=1 SV=2 |
| 73 | tr\|A0A2R8Y891\|A0A2R8Y891_HUMAN | 193.75 | 96908 | ATP-dependent 6-phosphofructokinase OS=Homo sapiens OX=9606 GN=PFKM PE=1 SV=1 |
| 74 | sp\|P30153\|2AAA_HUMAN | 193.62 | 65309 | Serine/threonine-protein phosphatase 2A 65 kDa regulatory subunit A alpha isoform OS=Homo sapiens OX=9606 GN=PPP2R1A PE=1 SV=4 |
| 75 | tr\|M0R3F1\|M0R3F1_HUMAN | 193.02 | 71730 | Heterogeneous nuclear ribonucleoprotein U-like protein 1 (Fragment) OS=Homo sapiens OX=9606 GN=HNRNPUL1 PE=1 SV=1 |
| 76 | tr\|A0A0A0MRA5\|A0A0A0MRA5_HUMAN | 193.02 | 85940 | Heterogeneous nuclear ribonucleoprotein U-like protein 1 OS=Homo sapiens OX=9606 GN=HNRNPUL1 PE=1 SV=1 |
| 77 | tr\|B7Z4B8\|B7Z4B8_HUMAN | 193.02 | 86122 | cDNA FLJ56481 highly similar to Heterogeneous nuclear ribonucleoprotein U-like protein 1 OS=Homo sapiens OX=9606 GN=HNRNPUL1 PE=1 SV=1 |
| 78 | sp\|Q9BUJ2\|HNRL1_HUMAN | 193.02 | 95739 | Heterogeneous nuclear ribonucleoprotein U-like protein 1 OS=Homo sapiens OX=9606 GN=HNRNPUL1 PE=1 SV=2 |
| 79 | sp\|Q9UBT2\|SAE2_HUMAN | 192.31 | 71224 | SUMO-activating enzyme subunit 2 OS=Homo sapiens OX=9606 GN=UBA2 PE=1 SV=2 |
| 80 | sp\|P60709\|ACTB_HUMAN | 191.68 | 41737 | Actin cytoplasmic 1 OS=Homo sapiens OX=9606 GN=ACTB PE=1 SV=1 |
| 81 | sp\|P63261\|ACTG_HUMAN | 191.68 | 41793 | Actin cytoplasmic 2 OS=Homo sapiens OX=9606 GN=ACTG1 PE=1 SV=1 |
| 82 | sp\|Q13200\|PSMD2_HUMAN | 191.27 | 100200 | 26S proteasome non-ATPase regulatory subunit 2 OS=Homo sapiens OX=9606 GN=PSMD2 PE=1 SV=3 |
| 83 | sp\|Q9NZI8\|IF2B1_HUMAN | 190.52 | 63481 | Insulin-like growth factor 2 mRNA-binding protein 1 OS=Homo sapiens OX=9606 GN=IGF2BP1 PE=1 SV=2 |
| 84 | sp\|Q8WUM4\|PDC6I_HUMAN | 188.98 | 96023 | Programmed cell death 6-interacting protein OS=Homo sapiens OX=9606 GN=PDCD6IP PE=1 SV=1 |
| 85 | sp\|P35606\|COPB2_HUMAN | 188.85 | 102487 | Coatomer subunit beta' OS=Homo sapiens OX=9606 GN=COPB2 PE=1 SV=2 |
| 86 | sp\|P14866\|HNRPL_HUMAN | 187.18 | 64133 | Heterogeneous nuclear ribonucleoprotein L OS=Homo sapiens OX=9606 GN=HNRNPL PE=1 SV=2 |
| 87 | tr\|M0QXS5\|M0QXS5_HUMAN | 187.18 | 58481 | Heterogeneous nuclear ribonucleoprotein L (Fragment) OS=Homo sapiens OX=9606 GN=HNRNPL PE=1 SV=1 |
| 88 | tr\|A0A3B3ITJ4\|A0A3B3ITJ4_HUMAN | 187.18 | 59219 | Heterogeneous nuclear ribonucleoprotein L (Fragment) OS=Homo sapiens OX=9606 GN=HNRNPL PE=1 SV=1 |
| 89 | sp\|Q96QK1\|VPS35_HUMAN | 185.58 | 91707 | Vacuolar protein sorting-associated protein 35 OS=Homo sapiens OX=9606 GN=VPS35 PE=1 SV=2 |
| 90 | sp\|Q99613\|EIF3C_HUMAN | 180.16 | 105344 | Eukaryotic translation initiation factor 3 subunit C OS=Homo sapiens OX=9606 GN=EIF3C PE=1 SV=1 |
| 91 | sp\|Q16643\|DREB_HUMAN | 178.98 | 71429 | Drebrin OS=Homo sapiens OX=9606 GN=DBN1 PE=1 SV=4 |
| 92 | sp\|P11586\|C1TC_HUMAN | 178.46 | 101559 | C-1-tetrahydrofolate synthase cytoplasmic OS=Homo sapiens OX=9606 GN=MTHFD1 PE=1 SV=3 |
| 93 | tr\|F5H2F4\|F5H2F4_HUMAN | 178.46 | 110613 | C-1-tetrahydrofolate synthase cytoplasmic OS=Homo sapiens OX=9606 GN=MTHFD1 PE=1 SV=1 |
| 94 | sp\|P68104\|EF1A1_HUMAN | 178.04 | 50141 | Elongation factor 1-alpha 1 OS=Homo sapiens OX=9606 GN=EEF1A1 PE=1 SV=1 |
| 95 | sp\|Q5VTE0\|EF1A3_HUMAN | 178.04 | 50185 | Putative elongation factor 1-alpha-like 3 OS=Homo sapiens OX=9606 GN=EEF1A1P5 PE=5 SV=1 |
| 96 | tr\|E9PK47\|E9PK47_HUMAN | 176.38 | 94070 | Alpha-1 4 glucan phosphorylase OS=Homo sapiens OX=9606 GN=PYGL PE=1 SV=1 |
| 97 | sp\|P06737\|PYGL_HUMAN | 176.38 | 97149 | Glycogen phosphorylase liver form OS=Homo sapiens OX=9606 GN=PYGL PE=1 SV=4 |
| 98 | sp\|Q99832\|TCPH_HUMAN | 174.04 | 59367 | T-complex protein 1 subunit eta OS=Homo sapiens OX=9606 GN=CCT7 PE=1 SV=2 |
| 99 | sp\|P13647\|K2C5_HUMAN | 173.77 | 62378 | Keratin type II cytoskeletal 5 OS=Homo sapiens OX=9606 GN=KRT5 PE=1 SV=3 |
| 100 | sp\|Q9BQE3\|TBA1C_HUMAN | 170.62 | 49895 | Tubulin alpha-1C chain OS=Homo sapiens OX=9606 GN=TUBA1C PE=1 SV=1 |
| 101 | sp\|Q71U36\|TBA1A_HUMAN | 170.62 | 50136 | Tubulin alpha-1A chain OS=Homo sapiens OX=9606 GN=TUBA1A PE=1 SV=1 |
| 102 | tr\|F5H5D3\|F5H5D3_HUMAN | 170.62 | 57730 | Tubulin alpha chain OS=Homo sapiens OX=9606 GN=TUBA1C PE=1 SV=1 |
| 103 | sp\|P68363\|TBA1B_HUMAN | 169.73 | 50152 | Tubulin alpha-1B chain OS=Homo sapiens OX=9606 GN=TUBA1B PE=1 SV=1 |
| 104 | sp\|P18206\|VINC_HUMAN | 169.69 | 123799 | Vinculin OS=Homo sapiens OX=9606 GN=VCL PE=1 SV=4 |
| 105 | sp\|P02452\|CO1A1_HUMAN | 168.95 | 138942 | Collagen alpha-1(I) chain OS=Homo sapiens OX=9606 GN=COL1A1 PE=1 SV=5 |
| 106 | sp\|P04843\|RPN1_HUMAN | 168.47 | 68569 | Dolichyl-diphosphooligosaccharide--protein glycosyltransferase subunit 1 OS=Homo sapiens OX=9606 GN=RPN1 PE=1 SV=1 |
| 107 | tr\|G3V1J5\|G3V1J5_HUMAN | 166.33 | 90584 | Exosome complex exonuclease RRP44 OS=Homo sapiens OX=9606 GN=DIS3 PE=1 SV=1 |
| 108 | sp\|Q9Y2L1\|RRP44_HUMAN | 166.33 | 109003 | Exosome complex exonuclease RRP44 OS=Homo sapiens OX=9606 GN=DIS3 PE=1 SV=2 |
| 109 | sp\|P49321\|NASP_HUMAN | 165.45 | 85238 | Nuclear autoantigenic sperm protein OS=Homo sapiens OX=9606 GN=NASP PE=1 SV=2 |
| 110 | sp\|O60506\|HNRPQ_HUMAN | 165.21 | 69603 | Heterogeneous nuclear ribonucleoprotein Q OS=Homo sapiens OX=9606 GN=SYNCRIP PE=1 SV=2 |
| 111 | sp\|P02533\|K1C14_HUMAN | 164.41 | 51562 | Keratin type I cytoskeletal 14 OS=Homo sapiens OX=9606 GN=KRT14 PE=1 SV=4 |
| 112 | sp\|Q86VP6\|CAND1_HUMAN | 159.18 | 136375 | Cullin-associated NEDD8-dissociated protein 1 OS=Homo sapiens OX=9606 GN=CAND1 PE=1 SV=2 |
| 113 | sp\|Q15029\|U5S1_HUMAN | 158.44 | 109436 | 116 kDa U5 small nuclear ribonucleoprotein component OS=Homo sapiens OX=9606 GN=EFTUD2 PE=1 SV=1 |
| 114 | sp\|Q92973\|TNPO1_HUMAN | 158.14 | 102355 | Transportin-1 OS=Homo sapiens OX=9606 GN=TNPO1 PE=1 SV=2 |
| 115 | sp\|Q9UBF2\|COPG2_HUMAN | 158.11 | 97622 | Coatomer subunit gamma-2 OS=Homo sapiens OX=9606 GN=COPG2 PE=1 SV=1 |
| 116 | sp\|P04844\|RPN2_HUMAN | 157.43 | 69284 | Dolichyl-diphosphooligosaccharide--protein glycosyltransferase subunit 2 OS=Homo sapiens OX=9606 GN=RPN2 PE=1 SV=3 |
| 117 | sp\|P55884\|EIF3B_HUMAN | 156.14 | 92482 | Eukaryotic translation initiation factor 3 subunit B OS=Homo sapiens OX=9606 GN=EIF3B PE=1 SV=3 |
| 118 | tr\|F5GZS6\|F5GZS6_HUMAN | 154.61 | 64873 | 4F2 cell-surface antigen heavy chain OS=Homo sapiens OX=9606 GN=SLC3A2 PE=1 SV=1 |
| 119 | sp\|P08195\|4F2_HUMAN | 154.61 | 67994 | 4F2 cell-surface antigen heavy chain OS=Homo sapiens OX=9606 GN=SLC3A2 PE=1 SV=3 |
| 120 | tr\|J3KPF3\|J3KPF3_HUMAN | 154.61 | 68101 | 4F2 cell-surface antigen heavy chain OS=Homo sapiens OX=9606 GN=SLC3A2 PE=1 SV=1 |
| 121 | tr\|A0A0C4DGX4\|A0A0C4DGX4_HUMAN | 153.92 | 87388 | Cullin-1 OS=Homo sapiens OX=9606 GN=CUL1 PE=1 SV=1 |
| 122 | sp\|Q13616\|CUL1_HUMAN | 153.92 | 89678 | Cullin-1 OS=Homo sapiens OX=9606 GN=CUL1 PE=1 SV=2 |
| 123 | sp\|P36578\|RL4_HUMAN | 153.39 | 47697 | 60S ribosomal protein L4 OS=Homo sapiens OX=9606 GN=RPL4 PE=1 SV=5 |
| 124 | sp\|Q08J23\|NSUN2_HUMAN | 152.3 | 86471 | tRNA (cytosine(34)-C(5))-methyltransferase OS=Homo sapiens OX=9606 GN=NSUN2 PE=1 SV=2 |
| 125 | sp\|Q08945\|SSRP1_HUMAN | 152.08 | 81075 | FACT complex subunit SSRP1 OS=Homo sapiens OX=9606 GN=SSRP1 PE=1 SV=1 |
| 126 | sp\|P49588\|SYAC_HUMAN | 151.65 | 106810 | Alanine--tRNA ligase cytoplasmic OS=Homo sapiens OX=9606 GN=AARS PE=1 SV=2 |
| 127 | sp\|O43242\|PSMD3_HUMAN | 151.05 | 60978 | 26S proteasome non-ATPase regulatory subunit 3 OS=Homo sapiens OX=9606 GN=PSMD3 PE=1 SV=2 |
| 128 | sp\|O43390\|HNRPR_HUMAN | 150.64 | 70943 | Heterogeneous nuclear ribonucleoprotein R OS=Homo sapiens OX=9606 GN=HNRNPR PE=1 SV=1 |

<table>
<tr><td>129</td><td>sp|P08779|K1C16_HUMAN</td><td>150.05</td><td>51268</td><td>Keratin  type I cytoskeletal 16 OS=Homo sapiens OX=9606 GN=KRT16 PE=1 SV=4</td></tr>
<tr><td>130</td><td>tr|A0A087WVC1|A0A087WVC1_HUMAN</td><td>147.5</td><td>82227</td><td>ATP-dependent RNA helicase DDX50 OS=Homo sapiens OX=9606 GN=DDX50 PE=1 SV=1</td></tr>
<tr><td>131</td><td>sp|Q9BQ39|DDX50_HUMAN</td><td>147.5</td><td>82565</td><td>ATP-dependent RNA helicase DDX50 OS=Homo sapiens OX=9606 GN=DDX50 PE=1 SV=1</td></tr>
<tr><td>132</td><td>sp|P04406|G3P_HUMAN</td><td>146.74</td><td>36053</td><td>Glyceraldehyde-3-phosphate dehydrogenase OS=Homo sapiens OX=9606 GN=GAPDH PE=1 SV=3</td></tr>
<tr><td>133</td><td>tr|E7EUT5|E7EUT5_HUMAN</td><td>146.74</td><td>27871</td><td>Glyceraldehyde-3-phosphate dehydrogenase OS=Homo sapiens OX=9606 GN=GAPDH PE=1 SV=1</td></tr>
<tr><td>134</td><td>sp|Q92841|DDX17_HUMAN</td><td>145.74</td><td>80273</td><td>Probable ATP-dependent RNA helicase DDX17 OS=Homo sapiens OX=9606 GN=DDX17 PE=1 SV=2</td></tr>
<tr><td>135</td><td>tr|A0A1X7SBZ2|A0A1X7SBZ2_HUMAN</td><td>145.74</td><td>80255</td><td>Probable ATP-dependent RNA helicase DDX17 OS=Homo sapiens OX=9606 GN=DDX17 PE=1 SV=1</td></tr>
<tr><td>136</td><td>tr|A0A1W2PQ51|A0A1W2PQ51_HUMAN</td><td>145.74</td><td>80458</td><td>Probable ATP-dependent RNA helicase DDX17 OS=Homo sapiens OX=9606 GN=DDX17 PE=1 SV=1</td></tr>
<tr><td>137</td><td>sp|Q13283|G3BP1_HUMAN</td><td>143.71</td><td>52164</td><td>Ras GTPase-activating protein-binding protein 1 OS=Homo sapiens OX=9606 GN=G3BP1 PE=1 SV=1</td></tr>
<tr><td>138</td><td>sp|O75131|CPNE3_HUMAN</td><td>143.28</td><td>60131</td><td>Copine-3 OS=Homo sapiens OX=9606 GN=CPNE3 PE=1 SV=1</td></tr>
<tr><td>139</td><td>sp|Q15233|NONO_HUMAN</td><td>142.47</td><td>54232</td><td>Non-POU domain-containing octamer-binding protein OS=Homo sapiens OX=9606 GN=NONO PE=1 SV=4</td></tr>
<tr><td>140</td><td>sp|Q9Y262|EIF3L_HUMAN</td><td>142.16</td><td>66727</td><td>Eukaryotic translation initiation factor 3 subunit L OS=Homo sapiens OX=9606 GN=EIF3L PE=1 SV=1</td></tr>
<tr><td>141</td><td>tr|B0QY89|B0QY89_HUMAN</td><td>142.16</td><td>70902</td><td>Eukaryotic translation initiation factor 3 subunit L OS=Homo sapiens OX=9606 GN=EIF3L PE=1 SV=1</td></tr>
<tr><td>142</td><td>sp|O00410|IPO5_HUMAN</td><td>141.05</td><td>123630</td><td>Importin-5 OS=Homo sapiens OX=9606 GN=IPO5 PE=1 SV=4</td></tr>
<tr><td>143</td><td>tr|H0Y8C6|H0Y8C6_HUMAN</td><td>141.05</td><td>123867</td><td>Importin-5 (Fragment) OS=Homo sapiens OX=9606 GN=IPO5 PE=1 SV=1</td></tr>
<tr><td>144</td><td>tr|H3BLV9|H3BLV9_HUMAN</td><td>140.42</td><td>76040</td><td>SRSF protein kinase 1 (Fragment) OS=Homo sapiens OX=9606 GN=SRPK1 PE=1 SV=1</td></tr>
<tr><td>145</td><td>sp|Q96SB4|SRPK1_HUMAN</td><td>140.42</td><td>74325</td><td>SRSF protein kinase 1 OS=Homo sapiens OX=9606 GN=SRPK1 PE=1 SV=2</td></tr>
<tr><td>146</td><td>tr|A0A0R4J2E8|A0A0R4J2E8_HUMAN</td><td>139.96</td><td>94623</td><td>Matrin-3 OS=Homo sapiens OX=9606 GN=MATR3 PE=1 SV=1</td></tr>
<tr><td>147</td><td>sp|P43243|MATR3_HUMAN</td><td>139.96</td><td>94623</td><td>Matrin-3 OS=Homo sapiens OX=9606 GN=MATR3 PE=1 SV=2</td></tr>
<tr><td>148</td><td>tr|A8MXP9|A8MXP9_HUMAN</td><td>139.96</td><td>99967</td><td>Matrin-3 OS=Homo sapiens OX=9606 GN=MATR3 PE=1 SV=1</td></tr>
<tr><td>149</td><td>sp|Q8WXF1|PSPC1_HUMAN</td><td>137.88</td><td>58744</td><td>Paraspeckle component 1 OS=Homo sapiens OX=9606 GN=PSPC1 PE=1 SV=1</td></tr>
<tr><td>150</td><td>sp|Q7KZF4|SND1_HUMAN</td><td>137.33</td><td>101997</td><td>Staphylococcal nuclease domain-containing protein 1 OS=Homo sapiens OX=9606 GN=SND1 PE=1 SV=1</td></tr>
<tr><td>151</td><td>tr|A0A3B3IU45|A0A3B3IU45_HUMAN</td><td>136.96</td><td>91191</td><td>DNA helicase OS=Homo sapiens OX=9606 GN=MCM4 PE=1 SV=1</td></tr>
<tr><td>152</td><td>sp|P33991|MCM4_HUMAN</td><td>136.96</td><td>96558</td><td>DNA replication licensing factor MCM4 OS=Homo sapiens OX=9606 GN=MCM4 PE=1 SV=5</td></tr>
<tr><td>153</td><td>tr|A0A087WTA8|A0A087WTA8_HUMAN</td><td>136.64</td><td>129151</td><td>Collagen alpha-2(I) chain OS=Homo sapiens OX=9606 GN=COL1A2 PE=1 SV=1</td></tr>
<tr><td>154</td><td>sp|P08123|CO1A2_HUMAN</td><td>136.64</td><td>129314</td><td>Collagen alpha-2(I) chain OS=Homo sapiens OX=9606 GN=COL1A2 PE=1 SV=7</td></tr>
<tr><td>155</td><td>sp|P08133|ANXA6_HUMAN</td><td>135.68</td><td>75873</td><td>Annexin A6 OS=Homo sapiens OX=9606 GN=ANXA6 PE=1 SV=3</td></tr>
<tr><td>156</td><td>sp|P55060|XPO2_HUMAN</td><td>135.55</td><td>110417</td><td>Exportin-2 OS=Homo sapiens OX=9606 GN=CSE1L PE=1 SV=3</td></tr>
<tr><td>157</td><td>sp|P50991|TCPD_HUMAN</td><td>135.13</td><td>57924</td><td>T-complex protein 1 subunit delta OS=Homo sapiens OX=9606 GN=CCT4 PE=1 SV=4</td></tr>
<tr><td>158</td><td>sp|P11021|BIP_HUMAN</td><td>134.99</td><td>72333</td><td>Endoplasmic reticulum chaperone BiP OS=Homo sapiens OX=9606 GN=HSPA5 PE=1 SV=2</td></tr>
<tr><td>159</td><td>sp|Q14651|PLSI_HUMAN</td><td>133.74</td><td>70254</td><td>Plastin-1 OS=Homo sapiens OX=9606 GN=PLS1 PE=1 SV=2</td></tr>
<tr><td>160</td><td>sp|O15226|NKRF_HUMAN</td><td>133.26</td><td>77673</td><td>NF-kappa-B-repressing factor OS=Homo sapiens OX=9606 GN=NKRF PE=1 SV=2</td></tr>
<tr><td>161</td><td>sp|Q9Y678|COPG1_HUMAN</td><td>133.22</td><td>97718</td><td>Coatomer subunit gamma-1 OS=Homo sapiens OX=9606 GN=COPG1 PE=1 SV=1</td></tr>
<tr><td>162</td><td>tr|A0A2R8Y804|A0A2R8Y804_HUMAN</td><td>133.08</td><td>77207</td><td>Catenin beta-1 OS=Homo sapiens OX=9606 GN=CTNNB1 PE=1 SV=1</td></tr>
<tr><td>163</td><td>tr|A0A2R8Y543|A0A2R8Y543_HUMAN</td><td>133.08</td><td>78405</td><td>Catenin beta-1 OS=Homo sapiens OX=9606 GN=CTNNB1 PE=1 SV=1</td></tr>
<tr><td>164</td><td>tr|B4DGU4|B4DGU4_HUMAN</td><td>133.08</td><td>84766</td><td>Catenin beta-1 OS=Homo sapiens OX=9606 GN=CTNNB1 PE=1 SV=1</td></tr>
<tr><td>165</td><td>tr|A0A2R8YCH5|A0A2R8YCH5_HUMAN</td><td>133.08</td><td>85268</td><td>Catenin beta-1 (Fragment) OS=Homo sapiens OX=9606 GN=CTNNB1 PE=1 SV=1</td></tr>
<tr><td>166</td><td>tr|A0A2R8Y7Z0|A0A2R8Y7Z0_HUMAN</td><td>133.08</td><td>85368</td><td>Catenin beta-1 OS=Homo sapiens OX=9606 GN=CTNNB1 PE=1 SV=1</td></tr>
<tr><td>167</td><td>sp|P35222|CTNB1_HUMAN</td><td>133.08</td><td>85497</td><td>Catenin beta-1 OS=Homo sapiens OX=9606 GN=CTNNB1 PE=1 SV=1</td></tr>
<tr><td>168</td><td>tr|A0A2R8Y5A3|A0A2R8Y5A3_HUMAN</td><td>133.08</td><td>85681</td><td>Catenin beta-1 OS=Homo sapiens OX=9606 GN=CTNNB1 PE=1 SV=1</td></tr>
<tr><td>169</td><td>tr|A0A2R8Y5C3|A0A2R8Y5C3_HUMAN</td><td>133.08</td><td>80699</td><td>Catenin beta-1 OS=Homo sapiens OX=9606 GN=CTNNB1 PE=1 SV=1</td></tr>
<tr><td>170</td><td>tr|A0A2R8Y750|A0A2R8Y750_HUMAN</td><td>133.08</td><td>81430</td><td>Catenin beta-1 OS=Homo sapiens OX=9606 GN=CTNNB1 PE=1 SV=1</td></tr>
<tr><td>171</td><td>tr|A0A2R8Y5Z1|A0A2R8Y5Z1_HUMAN</td><td>133.08</td><td>82426</td><td>Catenin beta-1 OS=Homo sapiens OX=9606 GN=CTNNB1 PE=1 SV=1</td></tr>
<tr><td>172</td><td>sp|P51116|FXR2_HUMAN</td><td>132.38</td><td>74223</td><td>Fragile X mental retardation syndrome-related protein 2 OS=Homo sapiens OX=9606 GN=FXR2 PE=1 SV=2</td></tr>
<tr><td>173</td><td>sp|Q9ULV4|COR1C_HUMAN</td><td>132.25</td><td>53249</td><td>Coronin-1C OS=Homo sapiens OX=9606 GN=CORO1C PE=1 SV=1</td></tr>
<tr><td>174</td><td>sp|P42224|STAT1_HUMAN</td><td>132.2</td><td>87335</td><td>Signal transducer and activator of transcription 1-alpha/beta OS=Homo sapiens OX=9606 GN=STAT1 PE=1 SV=2</td></tr>
<tr><td>175</td><td>sp|Q09161|NCBP1_HUMAN</td><td>131.74</td><td>91839</td><td>Nuclear cap-binding protein subunit 1 OS=Homo sapiens OX=9606 GN=NCBP1 PE=1 SV=1</td></tr>
<tr><td>176</td><td>sp|Q9NSD9|SYFB_HUMAN</td><td>129.52</td><td>66116</td><td>Phenylalanine--tRNA ligase beta subunit OS=Homo sapiens OX=9606 GN=FARSB PE=1 SV=3</td></tr>
<tr><td>177</td><td>tr|A0A0J9YYL3|A0A0J9YYL3_HUMAN</td><td>129.19</td><td>54627</td><td>Poly(U)-binding-splicing factor PUF60 (Fragment) OS=Homo sapiens OX=9606 GN=PUF60 PE=1 SV=1</td></tr>
<tr><td>178</td><td>tr|A0A0J9YXJ8|A0A0J9YXJ8_HUMAN</td><td>129.19</td><td>56331</td><td>Poly(U)-binding-splicing factor PUF60 (Fragment) OS=Homo sapiens OX=9606 GN=PUF60 PE=1 SV=1</td></tr>
<tr><td>179</td><td>tr|A0A0J9YVP6|A0A0J9YVP6_HUMAN</td><td>129.19</td><td>57399</td><td>Poly(U)-binding-splicing factor PUF60 (Fragment) OS=Homo sapiens OX=9606 GN=PUF60 PE=1 SV=1</td></tr>
<tr><td>180</td><td>tr|A0A0J9YXX5|A0A0J9YXX5_HUMAN</td><td>129.19</td><td>59103</td><td>Poly(U)-binding-splicing factor PUF60 (Fragment) OS=Homo sapiens OX=9606 GN=PUF60 PE=1 SV=1</td></tr>
<tr><td>181</td><td>sp|Q9UHX1|PUF60_HUMAN</td><td>129.19</td><td>59876</td><td>Poly(U)-binding-splicing factor PUF60 OS=Homo sapiens OX=9606 GN=PUF60 PE=1 SV=1</td></tr>
<tr><td>182</td><td>sp|Q96AE4|FUBP1_HUMAN</td><td>128.52</td><td>67560</td><td>Far upstream element-binding protein 1 OS=Homo sapiens OX=9606 GN=FUBP1 PE=1 SV=3</td></tr>
<tr><td>183</td><td>tr|E9PEB5|E9PEB5_HUMAN</td><td>128.52</td><td>68904</td><td>Far upstream element-binding protein 1 OS=Homo sapiens OX=9606 GN=FUBP1 PE=1 SV=1</td></tr>
<tr><td>184</td><td>sp|Q9P0K7|RAI14_HUMAN</td><td>127.93</td><td>110041</td><td>Ankycorbin OS=Homo sapiens OX=9606 GN=RAI14 PE=1 SV=2</td></tr>
<tr><td>185</td><td>sp|Q14684|RRP1B_HUMAN</td><td>127.91</td><td>84428</td><td>Ribosomal RNA processing protein 1 homolog B OS=Homo sapiens OX=9606 GN=RRP1B PE=1 SV=3</td></tr>
<tr><td>186</td><td>sp|O00139|KIF2A_HUMAN</td><td>126.4</td><td>79955</td><td>Kinesin-like protein KIF2A OS=Homo sapiens OX=9606 GN=KIF2A PE=1 SV=3</td></tr>
<tr><td>187</td><td>sp|P36871|PGM1_HUMAN</td><td>126.22</td><td>61449</td><td>Phosphoglucomutase-1 OS=Homo sapiens OX=9606 GN=PGM1 PE=1 SV=3</td></tr>
<tr><td>188</td><td>tr|A0A3B3ITK7|A0A3B3ITK7_HUMAN</td><td>126.22</td><td>63958</td><td>Phosphoglucomutase-1 OS=Homo sapiens OX=9606 GN=PGM1 PE=1 SV=1</td></tr>
<tr><td>189</td><td>sp|O43707|ACTN4_HUMAN</td><td>125.98</td><td>104854</td><td>Alpha-actinin-4 OS=Homo sapiens OX=9606 GN=ACTN4 PE=1 SV=2</td></tr>
<tr><td>190</td><td>sp|P22059|OSBP1_HUMAN</td><td>124.67</td><td>89421</td><td>Oxysterol-binding protein 1 OS=Homo sapiens OX=9606 GN=OSBP PE=1 SV=1</td></tr>
<tr><td>191</td><td>sp|P08621|RU17_HUMAN</td><td>124.1</td><td>51557</td><td>U1 small nuclear ribonucleoprotein 70 kDa OS=Homo sapiens OX=9606 GN=SNRNP70 PE=1 SV=2</td></tr>
<tr><td>192</td><td>sp|Q9BXJ9|NAA15_HUMAN</td><td>123.11</td><td>101272</td><td>N-alpha-acetyltransferase 15  NatA auxiliary subunit OS=Homo sapiens OX=9606 GN=NAA15 PE=1 SV=1</td></tr>
<tr><td>193</td><td>sp|Q86Y56|DAAF5_HUMAN</td><td>122.73</td><td>93521</td><td>Dynein assembly factor 5  axonemal OS=Homo sapiens OX=9606 GN=DNAAF5 PE=1 SV=4</td></tr>
<tr><td>194</td><td>sp|P61221|ABCE1_HUMAN</td><td>122.35</td><td>67314</td><td>ATP-binding cassette sub-family E member 1 OS=Homo sapiens OX=9606 GN=ABCE1 PE=1 SV=1</td></tr>
<tr><td>195</td><td>tr|D6R9I9|D6R9I9_HUMAN</td><td>122.35</td><td>47037</td><td>ATP-binding cassette sub-family E member 1 OS=Homo sapiens OX=9606 GN=ABCE1 PE=1 SV=1</td></tr>
<tr><td>196</td><td>sp|O00469|PLOD2_HUMAN</td><td>121.98</td><td>84686</td><td>Procollagen-lysine 2-oxoglutarate 5-dioxygenase 2 OS=Homo sapiens OX=9606 GN=PLOD2 PE=1 SV=2</td></tr>
<tr><td>197</td><td>tr|E7ETU9|E7ETU9_HUMAN</td><td>121.98</td><td>81170</td><td>Procollagen-lysine 2-oxoglutarate 5-dioxygenase 2 OS=Homo sapiens OX=9606 GN=PLOD2 PE=1 SV=1</td></tr>
<tr><td>198</td><td>sp|O43776|SYNC_HUMAN</td><td>121.98</td><td>62943</td><td>Asparagine--tRNA ligase  cytoplasmic OS=Homo sapiens OX=9606 GN=NARS PE=1 SV=1</td></tr>
<tr><td>199</td><td>sp|Q13617|CUL2_HUMAN</td><td>121.86</td><td>86983</td><td>Cullin-2 OS=Homo sapiens OX=9606 GN=CUL2 PE=1 SV=2</td></tr>
<tr><td>200</td><td>tr|A0A0A0MTN0|A0A0A0MTN0_HUMAN</td><td>121.86</td><td>88506</td><td>Cullin-2 OS=Homo sapiens OX=9606 GN=CUL2 PE=1 SV=1</td></tr>
<tr><td>201</td><td>tr|Q5T2B5|Q5T2B5_HUMAN</td><td>121.86</td><td>82359</td><td>Cullin-2 OS=Homo sapiens OX=9606 GN=CUL2 PE=1 SV=1</td></tr>
<tr><td>202</td><td>sp|P25205|MCM3_HUMAN</td><td>121.26</td><td>90981</td><td>DNA replication licensing factor MCM3 OS=Homo sapiens OX=9606 GN=MCM3 PE=1 SV=3</td></tr>
<tr><td>203</td><td>tr|A8MZH3|A8MZH3_HUMAN</td><td>120.08</td><td>19730</td><td>Myelin basic protein OS=Homo sapiens OX=9606 GN=MBP PE=1 SV=1</td></tr>
<tr><td>204</td><td>tr|J3QQK6|J3QQK6_HUMAN</td><td>120.08</td><td>27700</td><td>Myelin basic protein OS=Homo sapiens OX=9606 GN=MBP PE=1 SV=1</td></tr>
<tr><td>205</td><td>tr|E9PMR5|E9PMR5_HUMAN</td><td>120.08</td><td>10811</td><td>Myelin basic protein OS=Homo sapiens OX=9606 GN=MBP PE=1 SV=2</td></tr>
<tr><td>206</td><td>tr|H0YF02|H0YF02_HUMAN</td><td>120.08</td><td>11722</td><td>Myelin basic protein OS=Homo sapiens OX=9606 GN=MBP PE=1 SV=2</td></tr>
<tr><td>207</td><td>tr|H0YEA2|H0YEA2_HUMAN</td><td>120.08</td><td>13061</td><td>Myelin basic protein OS=Homo sapiens OX=9606 GN=MBP PE=1 SV=2</td></tr>
<tr><td>208</td><td>tr|J3QL64|J3QL64_HUMAN</td><td>120.08</td><td>17119</td><td>Myelin basic protein OS=Homo sapiens OX=9606 GN=MBP PE=1 SV=1</td></tr>
<tr><td>209</td><td>tr|J3QKL5|J3QKL5_HUMAN</td><td>120.08</td><td>18875</td><td>Myelin basic protein (Fragment) OS=Homo sapiens OX=9606 GN=MBP PE=1 SV=1</td></tr>
<tr><td>210</td><td>tr|H7BYR8|H7BYR8_HUMAN</td><td>120.08</td><td>25742</td><td>Myelin basic protein OS=Homo sapiens OX=9606 GN=MBP PE=1 SV=1</td></tr>
<tr><td>211</td><td>sp|P02686|MBP_HUMAN</td><td>120.08</td><td>33117</td><td>Myelin basic protein OS=Homo sapiens OX=9606 GN=MBP PE=1 SV=3</td></tr>
<tr><td>212</td><td>sp|O15371|EIF3D_HUMAN</td><td>119.84</td><td>63973</td><td>Eukaryotic translation initiation factor 3 subunit D OS=Homo sapiens OX=9606 GN=EIF3D PE=1 SV=1</td></tr>
<tr><td>213</td><td>tr|F8WJN3|F8WJN3_HUMAN</td><td>119.78</td><td>52270</td><td>Cleavage and polyadenylation-specificity factor subunit 6 OS=Homo sapiens OX=9606 GN=CPSF6 PE=1 SV=1</td></tr>
<tr><td>214</td><td>sp|Q16630|CPSF6_HUMAN</td><td>119.78</td><td>59210</td><td>Cleavage and polyadenylation specificity factor subunit 6 OS=Homo sapiens OX=9606 GN=CPSF6 PE=1 SV=2</td></tr>
<tr><td>215</td><td>tr|E7EQR4|E7EQR4_HUMAN</td><td>118.75</td><td>69372</td><td>Ezrin OS=Homo sapiens OX=9606 GN=EZR PE=1 SV=3</td></tr>
<tr><td>216</td><td>sp|P15311|EZRI_HUMAN</td><td>118.75</td><td>69413</td><td>Ezrin OS=Homo sapiens OX=9606 GN=EZR PE=1 SV=4</td></tr>
<tr><td>217</td><td>sp|P10515|ODP2_HUMAN</td><td>118.51</td><td>68997</td><td>Dihydrolipoyllysine-residue acetyltransferase component of pyruvate dehydrogenase complex  mitochondrial OS=Homo sapiens OX=9606 GN=DLAT P</td></tr>
<tr><td>218</td><td>tr|H0YDD4|H0YDD4_HUMAN</td><td>118.51</td><td>51201</td><td>Acetyltransferase component of pyruvate dehydrogenase complex (Fragment) OS=Homo sapiens OX=9606 GN=DLAT PE=1 SV=1</td></tr>
<tr><td>219</td><td>sp|P20700|LMNB1_HUMAN</td><td>118.45</td><td>66408</td><td>Lamin-B1 OS=Homo sapiens OX=9606 GN=LMNB1 PE=1 SV=2</td></tr>
<tr><td>220</td><td>sp|P35241|RADI_HUMAN</td><td>118.12</td><td>68564</td><td>Radixin OS=Homo sapiens OX=9606 GN=RDX PE=1 SV=1</td></tr>
<tr><td>221</td><td>tr|A0A2R8Y5S7|A0A2R8Y5S7_HUMAN</td><td>118.12</td><td>69336</td><td>Radixin OS=Homo sapiens OX=9606 GN=RDX PE=1 SV=1</td></tr>
<tr><td>222</td><td>tr|A0A2R8Y7M3|A0A2R8Y7M3_HUMAN</td><td>118.12</td><td>62931</td><td>Radixin OS=Homo sapiens OX=9606 GN=RDX PE=1 SV=1</td></tr>
<tr><td>223</td><td>tr|Q5T5C7|Q5T5C7_HUMAN</td><td>117.07</td><td>61313</td><td>Serine--tRNA ligase  cytoplasmic OS=Homo sapiens OX=9606 GN=SARS PE=1 SV=1</td></tr>
<tr><td>224</td><td>sp|P49591|SYSC_HUMAN</td><td>117.07</td><td>58777</td><td>Serine--tRNA ligase  cytoplasmic OS=Homo sapiens OX=9606 GN=SARS PE=1 SV=3</td></tr>
<tr><td>225</td><td>sp|P54886|P5CS_HUMAN</td><td>116.57</td><td>87302</td><td>Delta-1-pyrroline-5-carboxylate synthase OS=Homo sapiens OX=9606 GN=ALDH18A1 PE=1 SV=2</td></tr>
<tr><td>226</td><td>tr|H0Y8E6|H0Y8E6_HUMAN</td><td>116.52</td><td>94118</td><td>DNA helicase (Fragment) OS=Homo sapiens OX=9606 GN=MCM2 PE=1 SV=1</td></tr>
<tr><td>227</td><td>sp|P49736|MCM2_HUMAN</td><td>116.52</td><td>101896</td><td>DNA replication licensing factor MCM2 OS=Homo sapiens OX=9606 GN=MCM2 PE=1 SV=4</td></tr>
<tr><td>228</td><td>tr|H9KV75|H9KV75_HUMAN</td><td>115.95</td><td>94826</td><td>Alpha-actinin-1 OS=Homo sapiens OX=9606 GN=ACTN1 PE=1 SV=1</td></tr>
<tr><td>229</td><td>sp|P12814|ACTN1_HUMAN</td><td>115.95</td><td>103058</td><td>Alpha-actinin-1 OS=Homo sapiens OX=9606 GN=ACTN1 PE=1 SV=2</td></tr>
<tr><td>230</td><td>tr|B1AHC9|B1AHC9_HUMAN</td><td>115.46</td><td>64284</td><td>X-ray repair cross-complementing protein 6 OS=Homo sapiens OX=9606 GN=XRCC6 PE=1 SV=1</td></tr>
<tr><td>231</td><td>sp|P12956|XRCC6_HUMAN</td><td>115.46</td><td>69843</td><td>X-ray repair cross-complementing protein 6 OS=Homo sapiens OX=9606 GN=XRCC6 PE=1 SV=2</td></tr>
<tr><td>232</td><td>sp|Q9NVE7|PANK4_HUMAN</td><td>114.89</td><td>85991</td><td>Pantothenate kinase 4 OS=Homo sapiens OX=9606 GN=PANK4 PE=1 SV=1</td></tr>
<tr><td>233</td><td>sp|P16402|H13_HUMAN</td><td>114.81</td><td>22350</td><td>Histone H1.3 OS=Homo sapiens OX=9606 GN=HIST1H1D PE=1 SV=2</td></tr>
<tr><td>234</td><td>sp|P10412|H14_HUMAN</td><td>114.81</td><td>21865</td><td>Histone H1.4 OS=Homo sapiens OX=9606 GN=HIST1H1E PE=1 SV=2</td></tr>
<tr><td>235</td><td>sp|P16403|H12_HUMAN</td><td>114.81</td><td>21365</td><td>Histone H1.2 OS=Homo sapiens OX=9606 GN=HIST1H1C PE=1 SV=2</td></tr>
<tr><td>236</td><td>sp|P14923|PLAK_HUMAN</td><td>113.24</td><td>81745</td><td>Junction plakoglobin OS=Homo sapiens OX=9606 GN=JUP PE=1 SV=3</td></tr>
<tr><td>237</td><td>sp|P14314|GLU2B_HUMAN</td><td>112.69</td><td>59426</td><td>Glucosidase 2 subunit beta OS=Homo sapiens OX=9606 GN=PRKCSH PE=1 SV=2</td></tr>
<tr><td>238</td><td>tr|K7ELL7|K7ELL7_HUMAN</td><td>112.69</td><td>60192</td><td>Glucosidase 2 subunit beta OS=Homo sapiens OX=9606 GN=PRKCSH PE=1 SV=1</td></tr>
<tr><td>239</td><td>tr|A0A0U1RQH7|A0A0U1RQH7_HUMAN</td><td>112.58</td><td>26699</td><td>RNA-binding protein 39 (Fragment) OS=Homo sapiens OX=9606 GN=RBM39 PE=1 SV=1</td></tr>
<tr><td>240</td><td>tr|H0Y4X3|H0Y4X3_HUMAN</td><td>112.58</td><td>36633</td><td>RNA-binding protein 39 (Fragment) OS=Homo sapiens OX=9606 GN=RBM39 PE=1 SV=8</td></tr>
<tr><td>241</td><td>tr|G3XAC6|G3XAC6_HUMAN</td><td>112.58</td><td>48024</td><td>RNA-binding protein 39 OS=Homo sapiens OX=9606 GN=RBM39 PE=1 SV=1</td></tr>
<tr><td>242</td><td>sp|Q14498|RBM39_HUMAN</td><td>112.58</td><td>59380</td><td>RNA-binding protein 39 OS=Homo sapiens OX=9606 GN=RBM39 PE=1 SV=2</td></tr>
<tr><td>243</td><td>sp|O94874|UFL1_HUMAN</td><td>112.28</td><td>89595</td><td>E3 UFM1-protein ligase 1 OS=Homo sapiens OX=9606 GN=UFL1 PE=1 SV=2</td></tr>
<tr><td>244</td><td>sp|O95831|AIFM1_HUMAN</td><td>112.07</td><td>66901</td><td>Apoptosis-inducing factor 1  mitochondrial OS=Homo sapiens OX=9606 GN=AIFM1 PE=1 SV=1</td></tr>
<tr><td>245</td><td>sp|Q13724|MOGS_HUMAN</td><td>111.86</td><td>91918</td><td>Mannosyl-oligosaccharide glucosidase OS=Homo sapiens OX=9606 GN=MOGS PE=1 SV=5</td></tr>
<tr><td>246</td><td>sp|P78371|TCPB_HUMAN</td><td>111.78</td><td>57488</td><td>T-complex protein 1 subunit beta OS=Homo sapiens OX=9606 GN=CCT2 PE=1 SV=4</td></tr>
<tr><td>247</td><td>tr|F5GWF6|F5GWF6_HUMAN</td><td>111.78</td><td>56807</td><td>T-complex protein 1 subunit beta OS=Homo sapiens OX=9606 GN=CCT2 PE=1 SV=2</td></tr>
<tr><td>248</td><td>tr|F8VQ14|F8VQ14_HUMAN</td><td>111.78</td><td>44813</td><td>T-complex protein 1 subunit beta OS=Homo sapiens OX=9606 GN=CCT2 PE=1 SV=1</td></tr>
<tr><td>249</td><td>sp|O14556|G3PT_HUMAN</td><td>108.54</td><td>44501</td><td>Glyceraldehyde-3-phosphate dehydrogenase  testis-specific OS=Homo sapiens OX=9606 GN=GAPDHS PE=1 SV=2</td></tr>
<tr><td>250</td><td>sp|O43264|ZW10_HUMAN</td><td>108.49</td><td>88830</td><td>Centromere/kinetochore protein zw10 homolog OS=Homo sapiens OX=9606 GN=ZW10 PE=1 SV=3</td></tr>
<tr><td>251</td><td>sp|Q92945|FUBP2_HUMAN</td><td>107.77</td><td>73115</td><td>Far upstream element-binding protein 2 OS=Homo sapiens OX=9606 GN=KHSRP PE=1 SV=4</td></tr>
<tr><td>252</td><td>tr|A0A087WTP3|A0A087WTP3_HUMAN</td><td>107.77</td><td>73028</td><td>Far upstream element-binding protein 2 OS=Homo sapiens OX=9606 GN=KHSRP PE=1 SV=1</td></tr>
<tr><td>253</td><td>sp|P11216|PYGB_HUMAN</td><td>106.3</td><td>96696</td><td>Glycogen phosphorylase  brain form OS=Homo sapiens OX=9606 GN=PYGB PE=1 SV=5</td></tr>
<tr><td>254</td><td>sp|P33176|KINH_HUMAN</td><td>106.19</td><td>109685</td><td>Kinesin-1 heavy chain OS=Homo sapiens OX=9606 GN=KIF5B PE=1 SV=1</td></tr>
<tr><td>255</td><td>sp|O00425|IF2B3_HUMAN</td><td>105</td><td>63705</td><td>Insulin-like growth factor 2 mRNA-binding protein 3 OS=Homo sapiens OX=9606 GN=IGF2BP3 PE=1 SV=2</td></tr>
<tr><td>256</td><td>sp|Q14566|MCM6_HUMAN</td><td>104.26</td><td>92889</td><td>DNA replication licensing factor MCM6 OS=Homo sapiens OX=9606 GN=MCM6 PE=1 SV=1</td></tr>
<tr><td>257</td><td>sp|O75083|WDR1_HUMAN</td><td>102.46</td><td>66194</td><td>WD repeat-containing protein 1 OS=Homo sapiens OX=9606 GN=WDR1 PE=1 SV=4</td></tr>
</table>

| | | | | |
|---|---|---|---|---|
| 258 | sp\|Q15393\|SF3B3_HUMAN | 102.17 | 135577 | Splicing factor 3B subunit 3 OS=Homo sapiens OX=9606 GN=SF3B3 PE=1 SV=4 |
| 259 | sp\|P53597\|SUCA_HUMAN | 100.63 | 36250 | Succinate--CoA ligase [ADP/GDP-forming] subunit alpha mitochondrial OS=Homo sapiens OX=9606 GN=SUCLG1 PE=1 SV=4 |
| 260 | sp\|Q13423\|NNTM_HUMAN | 99.66 | 113895 | NAD(P) transhydrogenase mitochondrial OS=Homo sapiens OX=9606 GN=NNT PE=1 SV=3 |
| 261 | tr\|E9PCX7\|E9PCX7_HUMAN | 99.66 | 99729 | NAD(P) transhydrogenase mitochondrial OS=Homo sapiens OX=9606 GN=NNT PE=1 SV=1 |
| 262 | sp\|O43395\|PRPF3_HUMAN | 98.24 | 77529 | U4/U6 small nuclear ribonucleoprotein Prp3 OS=Homo sapiens OX=9606 GN=PRPF3 PE=1 SV=2 |
| 263 | sp\|P51610\|HCFC1_HUMAN | 96.71 | 208730 | Host cell factor 1 OS=Homo sapiens OX=9606 GN=HCFC1 PE=1 SV=2 |
| 264 | tr\|A6NEM2\|A6NEM2_HUMAN | 96.71 | 213474 | Host cell factor 1 OS=Homo sapiens OX=9606 GN=HCFC1 PE=1 SV=2 |
| 265 | sp\|Q07065\|CKAP4_HUMAN | 93.02 | 66023 | Cytoskeleton-associated protein 4 OS=Homo sapiens OX=9606 GN=CKAP4 PE=1 SV=2 |
| 266 | sp\|Q9Y697\|NFS1_HUMAN | 92.86 | 50196 | Cysteine desulfurase mitochondrial OS=Homo sapiens OX=9606 GN=NFS1 PE=1 SV=3 |
| 267 | tr\|H7C0I5\|H7C0I5_HUMAN | 92.86 | 15422 | Uncharacterized protein (Fragment) OS=Homo sapiens OX=9606 PE=4 SV=1 |
| 268 | tr\|H0YGN5\|H0YGN5_HUMAN | 92.86 | 17797 | Uncharacterized protein (Fragment) OS=Homo sapiens OX=9606 PE=4 SV=1 |
| 269 | tr\|H7BXI1\|H7BXI1_HUMAN | 92.14 | 98010 | Extended synaptotagmin-2 (Fragment) OS=Homo sapiens OX=9606 GN=ESYT2 PE=1 SV=1 |
| 270 | tr\|A0A087WXU3\|A0A087WXU3_HUMAN | 92.14 | 102372 | Extended synaptotagmin-2 OS=Homo sapiens OX=9606 GN=ESYT2 PE=1 SV=1 |
| 271 | sp\|A0FGR8\|ESYT2_HUMAN | 92.14 | 102357 | Extended synaptotagmin-2 OS=Homo sapiens OX=9606 GN=ESYT2 PE=1 SV=1 |
| 272 | sp\|P49411\|EFTU_HUMAN | 91.79 | 49542 | Elongation factor Tu mitochondrial OS=Homo sapiens OX=9606 GN=TUFM PE=1 SV=2 |
| 273 | sp\|Q16531\|DDB1_HUMAN | 90.23 | 126968 | DNA damage-binding protein 1 OS=Homo sapiens OX=9606 GN=DDB1 PE=1 SV=1 |
| 274 | tr\|F5GY55\|F5GY55_HUMAN | 90.23 | 121714 | DNA damage-binding protein 1 OS=Homo sapiens OX=9606 GN=DDB1 PE=1 SV=1 |
| 275 | sp\|Q93034\|CUL5_HUMAN | 89.77 | 90955 | Cullin-5 OS=Homo sapiens OX=9606 GN=CUL5 PE=1 SV=4 |
| 276 | sp\|Q04727\|TLE4_HUMAN | 89.76 | 83755 | Transducin-like enhancer protein 4 OS=Homo sapiens OX=9606 GN=TLE4 PE=1 SV=3 |
| 277 | sp\|Q04724\|TLE1_HUMAN | 89.76 | 83201 | Transducin-like enhancer protein 1 OS=Homo sapiens OX=9606 GN=TLE1 PE=1 SV=2 |
| 278 | tr\|H3BR35\|H3BR35_HUMAN | 89.66 | 52951 | Eukaryotic peptide chain release factor GTP-binding subunit ERF3A (Fragment) OS=Homo sapiens OX=9606 GN=GSPT1 PE=1 SV=1 |
| 279 | sp\|P15170\|ERF3A_HUMAN | 89.66 | 55756 | Eukaryotic peptide chain release factor GTP-binding subunit ERF3A OS=Homo sapiens OX=9606 GN=GSPT1 PE=1 SV=1 |
| 280 | sp\|Q86YZ3\|HORN_HUMAN | 89.6 | 282389 | Hornerin OS=Homo sapiens OX=9606 GN=HRNR PE=1 SV=2 |
| 281 | sp\|Q7Z2T5\|TRM1L_HUMAN | 88.74 | 81747 | TRMT1-like protein OS=Homo sapiens OX=9606 GN=TRMT1L PE=1 SV=2 |
| 282 | tr\|A0A3B3IS54\|A0A3B3IS54_HUMAN | 87.13 | 55163 | Aspartate--tRNA ligase mitochondrial OS=Homo sapiens OX=9606 GN=DARS2 PE=1 SV=1 |
| 283 | tr\|A0A3B3IT01\|A0A3B3IT01_HUMAN | 87.13 | 67686 | Aspartate--tRNA ligase mitochondrial OS=Homo sapiens OX=9606 GN=DARS2 PE=1 SV=1 |
| 284 | tr\|A0A3B3ISK7\|A0A3B3ISK7_HUMAN | 87.13 | 70987 | Aspartate--tRNA ligase mitochondrial OS=Homo sapiens OX=9606 GN=DARS2 PE=1 SV=1 |
| 285 | sp\|Q6PI48\|SYDM_HUMAN | 87.13 | 73563 | Aspartate--tRNA ligase mitochondrial OS=Homo sapiens OX=9606 GN=DARS2 PE=1 SV=1 |
| 286 | tr\|A0A2R8Y645\|A0A2R8Y645_HUMAN | 86.65 | 79475 | ATP-dependent RNA helicase DDX3X OS=Homo sapiens OX=9606 GN=DDX3X PE=1 SV=1 |
| 287 | tr\|A0A2R8YDT5\|A0A2R8YDT5_HUMAN | 86.65 | 52798 | ATP-dependent RNA helicase DDX3X OS=Homo sapiens OX=9606 GN=DDX3X PE=1 SV=1 |
| 288 | tr\|A0A2R8Y7T2\|A0A2R8Y7T2_HUMAN | 86.65 | 52885 | ATP-dependent RNA helicase DDX3X OS=Homo sapiens OX=9606 GN=DDX3X PE=1 SV=1 |
| 289 | tr\|A0A2R8Y5G6\|A0A2R8Y5G6_HUMAN | 86.65 | 69048 | ATP-dependent RNA helicase DDX3X OS=Homo sapiens OX=9606 GN=DDX3X PE=1 SV=1 |
| 290 | tr\|A0A0D9SFB3\|A0A0D9SFB3_HUMAN | 86.65 | 70840 | ATP-dependent RNA helicase DDX3X OS=Homo sapiens OX=9606 GN=DDX3X PE=1 SV=1 |
| 291 | tr\|A0A2R8YF78\|A0A2R8YF78_HUMAN | 86.65 | 70940 | ATP-dependent RNA helicase DDX3X OS=Homo sapiens OX=9606 GN=DDX3X PE=1 SV=1 |
| 292 | tr\|A0A2R8YCW1\|A0A2R8YCW1_HUMAN | 86.65 | 71694 | ATP-dependent RNA helicase DDX3X OS=Homo sapiens OX=9606 GN=DDX3X PE=1 SV=1 |
| 293 | tr\|A0A0D9SG12\|A0A0D9SG12_HUMAN | 86.65 | 71541 | ATP-dependent RNA helicase DDX3X OS=Homo sapiens OX=9606 GN=DDX3X PE=1 SV=1 |
| 294 | sp\|O15523\|DDX3Y_HUMAN | 86.65 | 73154 | ATP-dependent RNA helicase DDX3Y OS=Homo sapiens OX=9606 GN=DDX3Y PE=1 SV=2 |
| 295 | tr\|A0A2R8YFR4\|A0A2R8YFR4_HUMAN | 86.65 | 73127 | ATP-dependent RNA helicase DDX3X OS=Homo sapiens OX=9606 GN=DDX3X PE=1 SV=1 |
| 296 | tr\|A0A2R8YFS5\|A0A2R8YFS5_HUMAN | 86.65 | 73156 | ATP-dependent RNA helicase DDX3X OS=Homo sapiens OX=9606 GN=DDX3X PE=1 SV=1 |
| 297 | tr\|A0A2U3TZJ9\|A0A2U3TZJ9_HUMAN | 86.65 | 73215 | ATP-dependent RNA helicase DDX3X OS=Homo sapiens OX=9606 GN=DDX3X PE=1 SV=1 |
| 298 | tr\|A0A2R8Y4A4\|A0A2R8Y4A4_HUMAN | 86.65 | 73244 | ATP-dependent RNA helicase DDX3X OS=Homo sapiens OX=9606 GN=DDX3X PE=1 SV=1 |
| 299 | sp\|O00571\|DDX3X_HUMAN | 86.65 | 73244 | ATP-dependent RNA helicase DDX3X OS=Homo sapiens OX=9606 GN=DDX3X PE=1 SV=3 |
| 300 | tr\|A0A0D9SF53\|A0A0D9SF53_HUMAN | 86.65 | 81477 | ATP-dependent RNA helicase DDX3X OS=Homo sapiens OX=9606 GN=DDX3X PE=1 SV=1 |
| 301 | tr\|F6S8Q4\|F6S8Q4_HUMAN | 86.65 | 69356 | ATP-dependent RNA helicase DDX3X OS=Homo sapiens OX=9606 GN=DDX3X PE=1 SV=1 |
| 302 | tr\|A0A2R8YDH3\|A0A2R8YDH3_HUMAN | 86.65 | 56248 | ATP-dependent RNA helicase DDX3X OS=Homo sapiens OX=9606 GN=DDX3X PE=1 SV=1 |
| 303 | sp\|P21399\|ACOC_HUMAN | 85.04 | 98399 | Cytoplasmic aconitate hydratase OS=Homo sapiens OX=9606 GN=ACO1 PE=1 SV=3 |
| 304 | sp\|Q96SB3\|NEB2_HUMAN | 85.01 | 89192 | Neurabin-2 OS=Homo sapiens OX=9606 GN=PPP1R9B PE=1 SV=2 |
| 305 | tr\|D3DTX6\|D3DTX6_HUMAN | 85.01 | 89334 | Neurabin-2 OS=Homo sapiens OX=9606 GN=PPP1R9B PE=1 SV=1 |
| 306 | sp\|P42285\|MTREX_HUMAN | 84.98 | 117805 | Exosome RNA helicase MTR4 OS=Homo sapiens OX=9606 GN=MTREX PE=1 SV=3 |
| 307 | sp\|P25705\|ATPA_HUMAN | 84.66 | 59751 | ATP synthase subunit alpha mitochondrial OS=Homo sapiens OX=9606 GN=ATP5F1A PE=1 SV=1 |
| 308 | sp\|P06744\|G6PI_HUMAN | 83.77 | 63147 | Glucose-6-phosphate isomerase OS=Homo sapiens OX=9606 GN=GPI PE=1 SV=4 |
| 309 | tr\|A0A0A0MTS2\|A0A0A0MTS2_HUMAN | 83.77 | 64825 | Glucose-6-phosphate isomerase (Fragment) OS=Homo sapiens OX=9606 GN=GPI PE=1 SV=1 |
| 310 | tr\|A0A2U3TZU2\|A0A2U3TZU2_HUMAN | 83.77 | 67286 | Glucose-6-phosphate isomerase OS=Homo sapiens OX=9606 GN=GPI PE=1 SV=1 |
| 311 | tr\|K7EQ48\|K7EQ48_HUMAN | 83.77 | 50210 | Glucose-6-phosphate isomerase (Fragment) OS=Homo sapiens OX=9606 GN=GPI PE=1 SV=3 |
| 312 | sp\|Q14CN4\|K2C72_HUMAN | 82.24 | 55877 | Keratin type II cytoskeletal 72 OS=Homo sapiens OX=9606 GN=KRT72 PE=1 SV=2 |
| 313 | sp\|P02786\|TFR1_HUMAN | 81.34 | 84871 | Transferrin receptor protein 1 OS=Homo sapiens OX=9606 GN=TFRC PE=1 SV=2 |
| 314 | tr\|G3V0E5\|G3V0E5_HUMAN | 81.34 | 75964 | Transferrin receptor (P90 CD71) isoform CRA_c OS=Homo sapiens OX=9606 GN=TFRC PE=1 SV=1 |
| 315 | sp\|P31040\|SDHA_HUMAN | 81.15 | 72692 | Succinate dehydrogenase [ubiquinone] flavoprotein subunit mitochondrial OS=Homo sapiens OX=9606 GN=SDHA PE=1 SV=2 |
| 316 | sp\|Q9Y4W2\|LAS1L_HUMAN | 79.5 | 83065 | Ribosomal biogenesis protein LAS1L OS=Homo sapiens OX=9606 GN=LAS1L PE=1 SV=2 |
| 317 | tr\|H0YNJ6\|H0YNJ6_HUMAN | 78.37 | 46938 | GMP reductase OS=Homo sapiens OX=9606 GN=GMPR2 PE=1 SV=1 |
| 318 | tr\|H0YLV5\|H0YLV5_HUMAN | 78.37 | 35852 | GMP reductase OS=Homo sapiens OX=9606 GN=GMPR2 PE=1 SV=1 |
| 319 | tr\|H0YMB3\|H0YMB3_HUMAN | 78.37 | 33922 | GMP reductase OS=Homo sapiens OX=9606 GN=GMPR2 PE=1 SV=1 |
| 320 | tr\|A0A087WWM4\|A0A087WWM4_HUMA | 78.37 | 38217 | GMP reductase OS=Homo sapiens OX=9606 GN=GMPR2 PE=1 SV=1 |
| 321 | sp\|Q9P2T1\|GMPR2_HUMAN | 78.37 | 37874 | GMP reductase 2 OS=Homo sapiens OX=9606 GN=GMPR2 PE=1 SV=1 |
| 322 | tr\|F8WAN9\|F8WAN9_HUMAN | 78.37 | 38269 | GMP reductase OS=Homo sapiens OX=9606 GN=GMPR2 PE=1 SV=1 |
| 323 | tr\|H0YNH0\|H0YNH0_HUMAN | 78.37 | 26670 | GMP reductase 2 (Fragment) OS=Homo sapiens OX=9606 GN=GMPR2 PE=1 SV=8 |
| 324 | sp\|Q9Y3T9\|NOC2L_HUMAN | 76.5 | 84919 | Nucleolar complex protein 2 homolog OS=Homo sapiens OX=9606 GN=NOC2L PE=1 SV=4 |
| 325 | sp\|O95373\|IPO7_HUMAN | 75.92 | 119516 | Importin-7 OS=Homo sapiens OX=9606 GN=IPO7 PE=1 SV=1 |
| 326 | tr\|G3XAH6\|G3XAH6_HUMAN | 74.12 | 80626 | Poly(A) polymerase alpha OS=Homo sapiens OX=9606 GN=PAPOLA PE=1 SV=1 |
| 327 | sp\|P51003\|PAPOA_HUMAN | 74.12 | 82843 | Poly(A) polymerase alpha OS=Homo sapiens OX=9606 GN=PAPOLA PE=1 SV=4 |
| 328 | tr\|A0A2R8Y3X5\|A0A2R8Y3X5_HUMAN | 73.87 | 99133 | Dynamin-like 120 kDa protein mitochondrial OS=Homo sapiens OX=9606 GN=OPA1 PE=1 SV=1 |
| 329 | sp\|O60313\|OPA1_HUMAN | 73.87 | 111630 | Dynamin-like 120 kDa protein mitochondrial OS=Homo sapiens OX=9606 GN=OPA1 PE=1 SV=3 |
| 330 | tr\|A0A2R8YE78\|A0A2R8YE78_HUMAN | 73.87 | 112063 | Dynamin-like 120 kDa protein mitochondrial OS=Homo sapiens OX=9606 GN=OPA1 PE=1 SV=1 |
| 331 | tr\|A0A2R8YDM2\|A0A2R8YDM2_HUMAN | 73.87 | 112273 | Dynamin-like 120 kDa protein mitochondrial OS=Homo sapiens OX=9606 GN=OPA1 PE=1 SV=1 |
| 332 | tr\|C9JMB8\|C9JMB8_HUMAN | 73.87 | 112628 | Dynamin-like 120 kDa protein mitochondrial OS=Homo sapiens OX=9606 GN=OPA1 PE=1 SV=2 |
| 333 | tr\|E5KLJ9\|E5KLJ9_HUMAN | 73.87 | 113681 | Dynamin-like 120 kDa protein mitochondrial OS=Homo sapiens OX=9606 GN=OPA1 PE=1 SV=1 |
| 334 | tr\|Q5JP53\|Q5JP53_HUMAN | 73.52 | 47767 | Tubulin beta chain OS=Homo sapiens OX=9606 GN=TUBB PE=1 SV=1 |
| 335 | sp\|P07437\|TBB5_HUMAN | 73.52 | 49671 | Tubulin beta chain OS=Homo sapiens OX=9606 GN=TUBB PE=1 SV=2 |
| 336 | sp\|P06733\|ENOA_HUMAN | 73.35 | 47169 | Alpha-enolase OS=Homo sapiens OX=9606 GN=ENO1 PE=1 SV=2 |
| 337 | tr\|A0A2R8Y6G6\|A0A2R8Y6G6_HUMAN | 73.35 | 47327 | Alpha-enolase OS=Homo sapiens OX=9606 GN=ENO1 PE=1 SV=1 |
| 338 | sp\|P27694\|RFA1_HUMAN | 73.12 | 68138 | Replication protein A 70 kDa DNA-binding subunit OS=Homo sapiens OX=9606 GN=RPA1 PE=1 SV=2 |
| 339 | sp\|O14654\|IRS4_HUMAN | 72.82 | 133767 | Insulin receptor substrate 4 OS=Homo sapiens OX=9606 GN=IRS4 PE=1 SV=1 |
| 340 | sp\|Q15758\|AAAT_HUMAN | 72.72 | 56598 | Neutral amino acid transporter B(0) OS=Homo sapiens OX=9606 GN=SLC1A5 PE=1 SV=2 |
| 341 | tr\|M0QXM4\|M0QXM4_HUMAN | 72.72 | 39443 | Amino acid transporter OS=Homo sapiens OX=9606 GN=SLC1A5 PE=1 SV=1 |
| 342 | tr\|F8W6P5\|F8W6P5_HUMAN | 72.62 | 9670 | Hemoglobin subunit beta (Fragment) OS=Homo sapiens OX=9606 GN=HBB PE=1 SV=1 |
| 343 | tr\|A0A2R8Y7R2\|A0A2R8Y7R2_HUMAN | 72.62 | 12176 | Hemoglobin subunit beta OS=Homo sapiens OX=9606 GN=HBB PE=1 SV=1 |
| 344 | sp\|P68871\|HBB_HUMAN | 72.62 | 15998 | Hemoglobin subunit beta OS=Homo sapiens OX=9606 GN=HBB PE=1 SV=2 |
| 345 | tr\|A0A087X1B2\|A0A087X1B2_HUMAN | 71.36 | 56400 | U4/U6.U5 tri-snRNP-associated protein 2 OS=Homo sapiens OX=9606 GN=USP39 PE=1 SV=1 |
| 346 | sp\|Q53GS9\|SNUT2_HUMAN | 71.36 | 65381 | U4/U6.U5 tri-snRNP-associated protein 2 OS=Homo sapiens OX=9606 GN=USP39 PE=1 SV=2 |
| 347 | tr\|B9A018\|B9A018_HUMAN | 71.36 | 62044 | U4/U6.U5 tri-snRNP-associated protein 2 OS=Homo sapiens OX=9606 GN=USP39 PE=1 SV=1 |
| 348 | tr\|A0A087WW31\|A0A087WW31_HUMAN | 71.36 | 6756 | U4/U6.U5 tri-snRNP-associated protein 2 (Fragment) OS=Homo sapiens OX=9606 GN=USP39 PE=1 SV=1 |
| 349 | sp\|Q06210\|GFPT1_HUMAN | 71.36 | 78806 | Glutamine--fructose-6-phosphate aminotransferase [isomerizing] 1 OS=Homo sapiens OX=9606 GN=GFPT1 PE=1 SV=3 |
| 350 | sp\|Q15031\|SYLM_HUMAN | 70.75 | 101976 | Probable leucine--tRNA ligase mitochondrial OS=Homo sapiens OX=9606 GN=LARS2 PE=1 SV=2 |
| 351 | sp\|Q8N1G4\|LRC47_HUMAN | 70.35 | 63473 | Leucine-rich repeat-containing protein 47 OS=Homo sapiens OX=9606 GN=LRRC47 PE=1 SV=1 |
| 352 | sp\|Q12874\|SF3A3_HUMAN | 69.51 | 58849 | Splicing factor 3A subunit 3 OS=Homo sapiens OX=9606 GN=SF3A3 PE=1 SV=1 |
| 353 | sp\|Q969S9\|RRF2M_HUMAN | 69.42 | 86601 | Ribosome-releasing factor 2 mitochondrial OS=Homo sapiens OX=9606 GN=GFM2 PE=1 SV=1 |
| 354 | sp\|O43747\|AP1G1_HUMAN | 68.96 | 91351 | AP-1 complex subunit gamma-1 OS=Homo sapiens OX=9606 GN=AP1G1 PE=1 SV=5 |
| 355 | sp\|Q9UGP8\|SEC63_HUMAN | 68.33 | 87997 | Translocation protein SEC63 homolog OS=Homo sapiens OX=9606 GN=SEC63 PE=1 SV=2 |
| 356 | sp\|Q01518\|CAP1_HUMAN | 68.31 | 51902 | Adenylyl cyclase-associated protein 1 OS=Homo sapiens OX=9606 GN=CAP1 PE=1 SV=5 |
| 357 | tr\|Q5T0R7\|Q5T0R7_HUMAN | 68.31 | 19202 | Adenylyl cyclase-associated protein 1 (Fragment) OS=Homo sapiens OX=9606 GN=CAP1 PE=1 SV=1 |
| 358 | tr\|Q5T0R6\|Q5T0R6_HUMAN | 68.31 | 19416 | Adenylyl cyclase-associated protein 1 (Fragment) OS=Homo sapiens OX=9606 GN=CAP1 PE=1 SV=1 |
| 359 | tr\|Q5T0R5\|Q5T0R5_HUMAN | 68.31 | 19810 | Adenylyl cyclase-associated protein 1 (Fragment) OS=Homo sapiens OX=9606 GN=CAP1 PE=1 SV=8 |
| 360 | tr\|Q5T0R4\|Q5T0R4_HUMAN | 68.31 | 22532 | Adenylyl cyclase-associated protein 1 (Fragment) OS=Homo sapiens OX=9606 GN=CAP1 PE=1 SV=8 |
| 361 | tr\|Q5T0R3\|Q5T0R3_HUMAN | 68.31 | 22734 | Adenylyl cyclase-associated protein 1 (Fragment) OS=Homo sapiens OX=9606 GN=CAP1 PE=1 SV=1 |
| 362 | tr\|Q5T0R2\|Q5T0R2_HUMAN | 68.31 | 23249 | Adenylyl cyclase-associated protein 1 (Fragment) OS=Homo sapiens OX=9606 GN=CAP1 PE=1 SV=1 |
| 363 | tr\|Q5T0R1\|Q5T0R1_HUMAN | 68.31 | 23930 | Adenylyl cyclase-associated protein 1 (Fragment) OS=Homo sapiens OX=9606 GN=CAP1 PE=1 SV=1 |
| 364 | tr\|Q5T0R9\|Q5T0R9_HUMAN | 68.31 | 28635 | Adenylyl cyclase-associated protein 1 (Fragment) OS=Homo sapiens OX=9606 GN=CAP1 PE=1 SV=8 |
| 365 | sp\|P54136\|SYRC_HUMAN | 68.26 | 75379 | Arginine--tRNA ligase cytoplasmic OS=Homo sapiens OX=9606 GN=RARS PE=1 SV=2 |
| 366 | tr\|A0A0A0MSB4\|A0A0A0MSB4_HUMAN | 67.92 | 72219 | Oxysterol-binding protein OS=Homo sapiens OX=9606 GN=OSBP2 PE=1 SV=1 |
| 367 | tr\|Q6ZN50\|Q6ZN50_HUMAN | 67.92 | 83764 | Oxysterol-binding protein OS=Homo sapiens OX=9606 GN=OSBP2 PE=1 SV=1 |
| 368 | sp\|Q969R2\|OSBP2_HUMAN | 67.92 | 101266 | Oxysterol-binding protein 2 OS=Homo sapiens OX=9606 GN=OSBP2 PE=1 SV=2 |
| 369 | sp\|Q9BXP5\|SRRT_HUMAN | 67.86 | 100667 | Serrate RNA effector molecule homolog OS=Homo sapiens OX=9606 GN=SRRT PE=1 SV=1 |
| 370 | tr\|H7C3A1\|H7C3A1_HUMAN | 67.86 | 56734 | Serrate RNA effector molecule homolog (Fragment) OS=Homo sapiens OX=9606 GN=SRRT PE=1 SV=1 |
| 371 | tr\|A0A0A0MSP6\|A0A0A0MSP6_HUMAN | 67.86 | 16435 | Serrate RNA effector molecule homolog (Fragment) OS=Homo sapiens OX=9606 GN=SRRT PE=1 SV=6 |
| 372 | sp\|Q96HY7\|DHTK1_HUMAN | 67.73 | 103077 | Probable 2-oxoglutarate dehydrogenase E1 component DHKTD1 mitochondrial OS=Homo sapiens OX=9606 GN=DHTKD1 PE=1 SV=2 |
| 373 | sp\|Q8IXB1\|DJC10_HUMAN | 67.3 | 91080 | DnaJ homolog subfamily C member 10 OS=Homo sapiens OX=9606 GN=DNAJC10 PE=1 SV=2 |
| 374 | tr\|K4DI93\|K4DI93_HUMAN | 66.71 | 102756 | Cullin 4B isoform CRA_e OS=Homo sapiens OX=9606 GN=CUL4B PE=1 SV=1 |
| 375 | sp\|Q13620\|CUL4B_HUMAN | 66.71 | 103982 | Cullin-4B OS=Homo sapiens OX=9606 GN=CUL4B PE=1 SV=4 |
| 376 | tr\|H0Y2W2\|H0Y2W2_HUMAN | 66.52 | 64363 | ATPase family AAA domain-containing protein 3A (Fragment) OS=Homo sapiens OX=9606 GN=ATAD3A PE=1 SV=1 |
| 377 | sp\|Q9NVI7\|ATD3A_HUMAN | 66.52 | 71369 | ATPase family AAA domain-containing protein 3A OS=Homo sapiens OX=9606 GN=ATAD3A PE=1 SV=2 |
| 378 | sp\|Q5T9A4\|ATD3B_HUMAN | 66.52 | 72573 | ATPase family AAA domain-containing protein 3B OS=Homo sapiens OX=9606 GN=ATAD3B PE=1 SV=1 |
| 379 | sp\|Q6P996\|PDXD1_HUMAN | 66.31 | 86707 | Pyridoxal-dependent decarboxylase domain-containing protein 1 OS=Homo sapiens OX=9606 GN=PDXDC1 PE=1 SV=2 |
| 380 | tr\|H3BND4\|H3BND4_HUMAN | 66.31 | 88762 | Pyridoxal-dependent decarboxylase domain-containing protein 1 OS=Homo sapiens OX=9606 GN=PDXDC1 PE=1 SV=1 |
| 381 | tr\|Q86XE2\|Q86XE2_HUMAN | 66.31 | 54982 | PDXDC1 protein OS=Homo sapiens OX=9606 GN=PDXDC1 PE=1 SV=2 |
| 382 | tr\|Q6XYB5\|Q6XYB5_HUMAN | 66.31 | 22427 | LP8165 OS=Homo sapiens OX=9606 GN=PDXDC1 PE=1 SV=1 |
| 383 | sp\|P11387\|TOP1_HUMAN | 66.2 | 90726 | DNA topoisomerase 1 OS=Homo sapiens OX=9606 GN=TOP1 PE=1 SV=2 |
| 384 | sp\|Q07666\|KHDR1_HUMAN | 66.18 | 48227 | KH domain-containing RNA-binding signal transduction-associated protein 1 OS=Homo sapiens OX=9606 GN=KHDRBS1 PE=1 SV=1 |
| 385 | sp\|Q9BXB4\|OSB11_HUMAN | 66.11 | 83643 | Oxysterol-binding protein-related protein 11 OS=Homo sapiens OX=9606 GN=OSBPL11 PE=1 SV=2 |
| 386 | tr\|Q5T760\|Q5T760_HUMAN | 65.92 | 42316 | Serine/arginine-rich-splicing factor 11 (Fragment) OS=Homo sapiens OX=9606 GN=SRSF11 PE=1 SV=1 |

| | | | | |
|---|---|---|---|---|
| 387 | sp\|Q05519\|SRS11_HUMAN | 65.92 | 53542 | Serine/arginine-rich splicing factor 11 OS=Homo sapiens OX=9606 GN=SRSF11 PE=1 SV=1 |
| 388 | sp\|P23378\|GCSP_HUMAN | 65.88 | 112730 | Glycine dehydrogenase (decarboxylating)  mitochondrial OS=Homo sapiens OX=9606 GN=GLDC PE=1 SV=2 |
| 389 | tr\|A0A1W2PQV3\|A0A1W2PQV3_HUMAN | 65.88 | 42610 | Glycine dehydrogenase (decarboxylating)  mitochondrial OS=Homo sapiens OX=9606 GN=GLDC PE=1 SV=1 |
| 390 | tr\|A0A1W2PP74\|A0A1W2PP74_HUMAN | 65.88 | 46273 | Glycine dehydrogenase (decarboxylating)  mitochondrial OS=Homo sapiens OX=9606 GN=GLDC PE=1 SV=1 |
| 391 | sp\|Q9UKN8\|TF3C4_HUMAN | 65.44 | 91982 | General transcription factor 3C polypeptide 4 OS=Homo sapiens OX=9606 GN=GTF3C4 PE=1 SV=2 |
| 392 | sp\|P62979\|RS27A_HUMAN | 65.32 | 17965 | Ubiquitin-40S ribosomal protein S27a OS=Homo sapiens OX=9606 GN=RPS27A PE=1 SV=2 |
| 393 | sp\|P62987\|RL40_HUMAN | 65.32 | 14728 | Ubiquitin-60S ribosomal protein L40 OS=Homo sapiens OX=9606 GN=UBA52 PE=1 SV=2 |
| 394 | tr\|M0R2S1\|M0R2S1_HUMAN | 65.32 | 15476 | Ubiquitin-60S ribosomal protein L40 (Fragment) OS=Homo sapiens OX=9606 GN=UBA52 PE=1 SV=1 |
| 395 | tr\|J3QTR3\|J3QTR3_HUMAN | 65.32 | 12237 | Ubiquitin-40S ribosomal protein S27a (Fragment) OS=Homo sapiens OX=9606 GN=RPS27A PE=1 SV=1 |
| 396 | tr\|J3QS39\|J3QS39_HUMAN | 65.32 | 10469 | Polyubiquitin-B (Fragment) OS=Homo sapiens OX=9606 GN=UBB PE=1 SV=1 |
| 397 | tr\|F5H6Q2\|F5H6Q2_HUMAN | 65.32 | 13732 | Polyubiquitin-C (Fragment) OS=Homo sapiens OX=9606 GN=UBC PE=1 SV=8 |
| 398 | tr\|F5GYU3\|F5GYU3_HUMAN | 65.32 | 15032 | Polyubiquitin-C (Fragment) OS=Homo sapiens OX=9606 GN=UBC PE=1 SV=1 |
| 399 | tr\|F5H2Z3\|F5H2Z3_HUMAN | 65.32 | 15310 | Polyubiquitin-C (Fragment) OS=Homo sapiens OX=9606 GN=UBC PE=1 SV=1 |
| 400 | tr\|F5H265\|F5H265_HUMAN | 65.32 | 16841 | Polyubiquitin-C (Fragment) OS=Homo sapiens OX=9606 GN=UBC PE=1 SV=1 |
| 401 | tr\|B4DV12\|B4DV12_HUMAN | 65.32 | 17215 | Polyubiquitin-B OS=Homo sapiens OX=9606 GN=UBB PE=1 SV=1 |
| 402 | tr\|F5H747\|F5H747_HUMAN | 65.32 | 18073 | Polyubiquitin-C (Fragment) OS=Homo sapiens OX=9606 GN=UBC PE=1 SV=8 |
| 403 | tr\|F5GXK7\|F5GXK7_HUMAN | 65.32 | 19016 | Polyubiquitin-C (Fragment) OS=Homo sapiens OX=9606 GN=UBC PE=1 SV=2 |
| 404 | tr\|J3QKN0\|J3QKN0_HUMAN | 65.32 | 23163 | Polyubiquitin-B (Fragment) OS=Homo sapiens OX=9606 GN=UBB PE=1 SV=1 |
| 405 | sp\|P0CG47\|UBB_HUMAN | 65.32 | 25762 | Polyubiquitin-B OS=Homo sapiens OX=9606 GN=UBB PE=1 SV=1 |
| 406 | sp\|P0CG48\|UBC_HUMAN | 65.32 | 77039 | Polyubiquitin-C OS=Homo sapiens OX=9606 GN=UBC PE=1 SV=3 |
| 407 | sp\|Q32P28\|P3H1_HUMAN | 65.03 | 83394 | Prolyl 3-hydroxylase 1 OS=Homo sapiens OX=9606 GN=P3H1 PE=1 SV=2 |
| 408 | tr\|E9PKI2\|E9PKI2_HUMAN | 64.39 | 8602 | Nucleosome assembly protein 1-like 4 (Fragment) OS=Homo sapiens OX=9606 GN=NAP1L4 PE=1 SV=1 |
| 409 | tr\|E9PNW0\|E9PNW0_HUMAN | 64.39 | 12250 | Nucleosome assembly protein 1-like 4 (Fragment) OS=Homo sapiens OX=9606 GN=NAP1L4 PE=1 SV=1 |
| 410 | tr\|E9PJJ2\|E9PJJ2_HUMAN | 64.39 | 14653 | Nucleosome assembly protein 1-like 4 (Fragment) OS=Homo sapiens OX=9606 GN=NAP1L4 PE=1 SV=1 |
| 411 | tr\|E9PS34\|E9PS34_HUMAN | 64.39 | 15282 | Nucleosome assembly protein 1-like 4 (Fragment) OS=Homo sapiens OX=9606 GN=NAP1L4 PE=1 SV=1 |
| 412 | tr\|E9PNJ7\|E9PNJ7_HUMAN | 64.39 | 16000 | Nucleosome assembly protein 1-like 4 (Fragment) OS=Homo sapiens OX=9606 GN=NAP1L4 PE=1 SV=1 |
| 413 | tr\|E9PP22\|E9PP22_HUMAN | 64.39 | 17350 | Nucleosome assembly protein 1-like 4 (Fragment) OS=Homo sapiens OX=9606 GN=NAP1L4 PE=1 SV=2 |
| 414 | tr\|C9J6D1\|C9J6D1_HUMAN | 64.39 | 20261 | Nucleosome assembly protein 1-like 4 (Fragment) OS=Homo sapiens OX=9606 GN=NAP1L4 PE=1 SV=1 |
| 415 | sp\|Q99733\|NP1L4_HUMAN | 64.39 | 42823 | Nucleosome assembly protein 1-like 4 OS=Homo sapiens OX=9606 GN=NAP1L4 PE=1 SV=1 |
| 416 | tr\|C9J1B1\|C9J1B1_HUMAN | 64.39 | 11405 | Nucleosome assembly protein 1-like 4 (Fragment) OS=Homo sapiens OX=9606 GN=NAP1L4 PE=1 SV=2 |
| 417 | tr\|E9PKT8\|E9PKT8_HUMAN | 64.39 | 19156 | Nucleosome assembly protein 1-like 4 (Fragment) OS=Homo sapiens OX=9606 GN=NAP1L4 PE=1 SV=1 |
| 418 | tr\|C9JZI7\|C9JZI7_HUMAN | 64.39 | 31840 | Nucleosome assembly protein 1-like 4 (Fragment) OS=Homo sapiens OX=9606 GN=NAP1L4 PE=1 SV=1 |
| 419 | sp\|P53985\|MOT1_HUMAN | 63.11 | 53944 | Monocarboxylate transporter 1 OS=Homo sapiens OX=9606 GN=SLC16A1 PE=1 SV=3 |
| 420 | tr\|Q5T8R3\|Q5T8R3_HUMAN | 63.11 | 31689 | Monocarboxylate transporter 1 (Fragment) OS=Homo sapiens OX=9606 GN=SLC16A1 PE=1 SV=1 |
| 421 | tr\|Q5T8R5\|Q5T8R5_HUMAN | 63.11 | 49219 | Monocarboxylate transporter 1 (Fragment) OS=Homo sapiens OX=9606 GN=SLC16A1 PE=1 SV=1 |
| 422 | tr\|F8VX11\|F8VX11_HUMAN | 62.91 | 41455 | Eukaryotic translation initiation factor 4B (Fragment) OS=Homo sapiens OX=9606 GN=EIF4B PE=1 SV=1 |
| 423 | sp\|P23588\|IF4B_HUMAN | 62.91 | 69151 | Eukaryotic translation initiation factor 4B OS=Homo sapiens OX=9606 GN=EIF4B PE=1 SV=2 |
| 424 | tr\|E7EX17\|E7EX17_HUMAN | 62.91 | 69698 | Eukaryotic translation initiation factor 4B OS=Homo sapiens OX=9606 GN=EIF4B PE=1 SV=1 |
| 425 | sp\|P08670\|VIME_HUMAN | 62.08 | 53652 | Vimentin OS=Homo sapiens OX=9606 GN=VIM PE=1 SV=4 |
| 426 | tr\|B0YJC4\|B0YJC4_HUMAN | 62.08 | 49653 | Vimentin variant 3 OS=Homo sapiens OX=9606 GN=VIM PE=1 SV=1 |
| 427 | sp\|Q5SY16\|NOL9_HUMAN | 62.01 | 79323 | Polynucleotide 5'-hydroxyl-kinase NOL9 OS=Homo sapiens OX=9606 GN=NOL9 PE=1 SV=1 |
| 428 | sp\|P09960\|LKHA4_HUMAN | 61.62 | 69285 | Leukotriene A-4 hydrolase OS=Homo sapiens OX=9606 GN=LTA4H PE=1 SV=2 |
| 429 | sp\|Q96S59\|RANB9_HUMAN | 61.24 | 77847 | Ran-binding protein 9 OS=Homo sapiens OX=9606 GN=RANBP9 PE=1 SV=1 |
| 430 | tr\|F5H365\|F5H365_HUMAN | 60.15 | 82969 | Protein transport protein SEC23 OS=Homo sapiens OX=9606 GN=SEC23A PE=1 SV=1 |
| 431 | sp\|Q15436\|SC23A_HUMAN | 60.15 | 86161 | Protein transport protein Sec23A OS=Homo sapiens OX=9606 GN=SEC23A PE=1 SV=2 |
| 432 | sp\|Q9BW62\|KATL1_HUMAN | 59.75 | 55392 | Katanin p60 ATPase-containing subunit A-like 1 OS=Homo sapiens OX=9606 GN=KATNAL1 PE=1 SV=1 |
| 433 | tr\|B7ZBC8\|B7ZBC8_HUMAN | 59.75 | 31829 | Katanin p60 ATPase-containing subunit A1 (Fragment) OS=Homo sapiens OX=9606 GN=KATNA1 PE=1 SV=1 |
| 434 | sp\|O75449\|KTNA1_HUMAN | 59.75 | 55965 | Katanin p60 ATPase-containing subunit A1 OS=Homo sapiens OX=9606 GN=KATNA1 PE=1 SV=1 |
| 435 | tr\|C9J470\|C9J470_HUMAN | 59.32 | 68012 | Condensin complex subunit 2 (Fragment) OS=Homo sapiens OX=9606 GN=NCAPH PE=1 SV=8 |
| 436 | tr\|E9PHA2\|E9PHA2_HUMAN | 59.32 | 81573 | Condensin complex subunit 2 OS=Homo sapiens OX=9606 GN=NCAPH PE=1 SV=1 |
| 437 | sp\|Q15003\|CND2_HUMAN | 59.32 | 82563 | Condensin complex subunit 2 OS=Homo sapiens OX=9606 GN=NCAPH PE=1 SV=3 |
| 438 | tr\|F8W7U8\|F8W7U8_HUMAN | 58.35 | 80522 | Double-strand break repair protein OS=Homo sapiens OX=9606 GN=MRE11 PE=1 SV=1 |
| 439 | sp\|P49959\|MRE11_HUMAN | 58.35 | 80593 | Double-strand break repair protein MRE11 OS=Homo sapiens OX=9606 GN=MRE11 PE=1 SV=3 |
| 440 | sp\|Q9Y6Y8\|S23IP_HUMAN | 58.34 | 111076 | SEC23-interacting protein OS=Homo sapiens OX=9606 GN=SEC23IP PE=1 SV=1 |
| 441 | tr\|A0A0C4DH82\|A0A0C4DH82_HUMAN | 58.34 | 53153 | SEC23-interacting protein OS=Homo sapiens OX=9606 GN=SEC23IP PE=1 SV=1 |
| 442 | sp\|P46977\|STT3A_HUMAN | 58.33 | 80530 | Dolichyl-diphosphooligosaccharide--protein glycosyltransferase subunit STT3A OS=Homo sapiens OX=9606 GN=STT3A PE=1 SV=2 |
| 443 | sp\|Q96TA1\|NIBL1_HUMAN | 58.29 | 84138 | Niban-like protein 1 OS=Homo sapiens OX=9606 GN=FAM129B PE=1 SV=3 |
| 444 | tr\|Q5JR04\|Q5JR04_HUMAN | 58.26 | 107210 | Helicase MOV-10 OS=Homo sapiens OX=9606 GN=MOV10 PE=1 SV=1 |
| 445 | sp\|Q9HCE1\|MOV10_HUMAN | 58.26 | 113671 | Helicase MOV-10 OS=Homo sapiens OX=9606 GN=MOV10 PE=1 SV=2 |
| 446 | tr\|B4DKY1\|B4DKY1_HUMAN | 57.7 | 84278 | Cysteine--tRNA ligase  cytoplasmic OS=Homo sapiens OX=9606 GN=CARS PE=1 SV=1 |
| 447 | sp\|P11279\|LAMP1_HUMAN | 57.7 | 44882 | Lysosome-associated membrane glycoprotein 1 OS=Homo sapiens OX=9606 GN=LAMP1 PE=1 SV=3 |
| 448 | sp\|Q9H8H0\|NOL11_HUMAN | 57.62 | 81124 | Nucleolar protein 11 OS=Homo sapiens OX=9606 GN=NOL11 PE=1 SV=1 |
| 449 | tr\|J3QLQ6\|J3QLQ6_HUMAN | 57.62 | 21313 | Nucleolar protein 11 (Fragment) OS=Homo sapiens OX=9606 GN=NOL11 PE=1 SV=1 |
| 450 | sp\|O76031\|CLPX_HUMAN | 56.72 | 69224 | ATP-dependent Clp protease ATP-binding subunit clpX-like  mitochondrial OS=Homo sapiens OX=9606 GN=CLPX PE=1 SV=2 |
| 451 | sp\|P35520\|CBS_HUMAN | 56.56 | 60587 | Cystathionine beta-synthase OS=Homo sapiens OX=9606 GN=CBS PE=1 SV=2 |
| 452 | sp\|P0DN79\|CBSL_HUMAN | 56.56 | 60587 | Cystathionine beta-synthase-like protein OS=Homo sapiens OX=9606 GN=CBSL PE=1 SV=1 |
| 453 | sp\|P13807\|GYS1_HUMAN | 54.92 | 83786 | Glycogen [starch] synthase  muscle OS=Homo sapiens OX=9606 GN=GYS1 PE=1 SV=2 |
| 454 | sp\|A0MZ66\|SHOT1_HUMAN | 53.23 | 71640 | Shootin-1 OS=Homo sapiens OX=9606 GN=SHTN1 PE=1 SV=4 |
| 455 | sp\|O14744\|ANM5_HUMAN | 53.06 | 72684 | Protein arginine N-methyltransferase 5 OS=Homo sapiens OX=9606 GN=PRMT5 PE=1 SV=4 |
| 456 | tr\|H0YJX6\|H0YJX6_HUMAN | 53.06 | 32312 | Protein arginine N-methyltransferase 5 (Fragment) OS=Homo sapiens OX=9606 GN=PRMT5 PE=1 SV=1 |
| 457 | tr\|A0A087X0X3\|A0A087X0X3_HUMAN | 52.78 | 77570 | Heterogeneous nuclear ribonucleoprotein M OS=Homo sapiens OX=9606 GN=HNRNPM PE=1 SV=1 |
| 458 | sp\|P52272\|HNRPM_HUMAN | 52.78 | 77516 | Heterogeneous nuclear ribonucleoprotein M OS=Homo sapiens OX=9606 GN=HNRNPM PE=1 SV=3 |
| 459 | tr\|M0R019\|M0R019_HUMAN | 52.78 | 37627 | Heterogeneous nuclear ribonucleoprotein M (Fragment) OS=Homo sapiens OX=9606 GN=HNRNPM PE=1 SV=8 |
| 460 | tr\|M0R0Y6\|M0R0Y6_HUMAN | 52.78 | 10739 | Heterogeneous nuclear ribonucleoprotein M OS=Homo sapiens OX=9606 GN=HNRNPM PE=1 SV=1 |
| 461 | tr\|M0QYQ7\|M0QYQ7_HUMAN | 52.78 | 24114 | Heterogeneous nuclear ribonucleoprotein M (Fragment) OS=Homo sapiens OX=9606 GN=HNRNPM PE=1 SV=1 |
| 462 | tr\|M0R0N3\|M0R0N3_HUMAN | 52.78 | 30082 | Heterogeneous nuclear ribonucleoprotein M (Fragment) OS=Homo sapiens OX=9606 GN=HNRNPM PE=1 SV=8 |
| 463 | tr\|M0R2T0\|M0R2T0_HUMAN | 52.78 | 38226 | Heterogeneous nuclear ribonucleoprotein M (Fragment) OS=Homo sapiens OX=9606 GN=HNRNPM PE=1 SV=1 |
| 464 | sp\|Q13435\|SF3B2_HUMAN | 51.84 | 100228 | Splicing factor 3B subunit 2 OS=Homo sapiens OX=9606 GN=SF3B2 PE=1 SV=2 |
| 465 | tr\|E9PPJ0\|E9PPJ0_HUMAN | 51.84 | 98170 | Splicing factor 3B subunit 2 OS=Homo sapiens OX=9606 GN=SF3B2 PE=1 SV=1 |
| 466 | tr\|H0YEX5\|H0YEX5_HUMAN | 51.84 | 35017 | Splicing factor 3B subunit 2 (Fragment) OS=Homo sapiens OX=9606 GN=SF3B2 PE=1 SV=1 |
| 467 | sp\|P07196\|NFL_HUMAN | 50.57 | 61517 | Neurofilament light polypeptide OS=Homo sapiens OX=9606 GN=NEFL PE=1 SV=3 |
| 468 | sp\|Q9NVH1\|DJC11_HUMAN | 50.54 | 63278 | DnaJ homolog subfamily C member 11 OS=Homo sapiens OX=9606 GN=DNAJC11 PE=1 SV=2 |
| 469 | tr\|H0Y3P2\|H0Y3P2_HUMAN | 50.2 | 98118 | Eukaryotic translation initiation factor 4 gamma 2 OS=Homo sapiens OX=9606 GN=EIF4G2 PE=1 SV=1 |
| 470 | tr\|D3DQV9\|D3DQV9_HUMAN | 50.2 | 102330 | Eukaryotic translation initiation factor 4 gamma 2 (Fragment) OS=Homo sapiens OX=9606 GN=EIF4G2 PE=1 SV=1 |
| 471 | sp\|P78344\|IF4G2_HUMAN | 50.2 | 102362 | Eukaryotic translation initiation factor 4 gamma 2 OS=Homo sapiens OX=9606 GN=EIF4G2 PE=1 SV=1 |
| 472 | sp\|Q8TAT6\|NPL4_HUMAN | 49.92 | 68120 | Nuclear protein localization protein 4 homolog OS=Homo sapiens OX=9606 GN=NPLOC4 PE=1 SV=3 |
| 473 | sp\|P17174\|AATC_HUMAN | 49.8 | 46248 | Aspartate aminotransferase  cytoplasmic OS=Homo sapiens OX=9606 GN=GOT1 PE=1 SV=3 |
| 474 | sp\|Q8TE77\|SSH3_HUMAN | 49.75 | 72996 | Protein phosphatase Slingshot homolog 3 OS=Homo sapiens OX=9606 GN=SSH3 PE=1 SV=2 |
| 475 | tr\|A0A087WWG1\|A0A087WWG1_HUMA | 49.75 | 36103 | Protein phosphatase Slingshot homolog 3 (Fragment) OS=Homo sapiens OX=9606 GN=SSH3 PE=1 SV=1 |
| 476 | tr\|E9PIT5\|E9PIT5_HUMAN | 49.75 | 24747 | Protein phosphatase Slingshot homolog 3 (Fragment) OS=Homo sapiens OX=9606 GN=SSH3 PE=1 SV=2 |
| 477 | sp\|Q99460\|PSMD1_HUMAN | 48.78 | 105836 | 26S proteasome non-ATPase regulatory subunit 1 OS=Homo sapiens OX=9606 GN=PSMD1 PE=1 SV=2 |
| 478 | tr\|A0A087WW66\|A0A087WW66_HUMAI | 48.78 | 105851 | 26S proteasome non-ATPase regulatory subunit 1 OS=Homo sapiens OX=9606 GN=PSMD1 PE=1 SV=1 |
| 479 | sp\|Q9Y2A7\|NCKP1_HUMAN | 48.24 | 128790 | Nck-associated protein 1 OS=Homo sapiens OX=9606 GN=NCKAP1 PE=1 SV=1 |
| 480 | sp\|Q08AM6\|VAC14_HUMAN | 48.22 | 87973 | Protein VAC14 homolog OS=Homo sapiens OX=9606 GN=VAC14 PE=1 SV=1 |
| 481 | sp\|Q13618\|CUL3_HUMAN | 48.06 | 88930 | Cullin-3 OS=Homo sapiens OX=9606 GN=CUL3 PE=1 SV=2 |
| 482 | tr\|A0A087X1R9\|A0A087X1R9_HUMAN | 48.06 | 6485 | Cullin-3 (Fragment) OS=Homo sapiens OX=9606 GN=CUL3 PE=1 SV=1 |
| 483 | tr\|H7C1L6\|H7C1L6_HUMAN | 48.06 | 22662 | Cullin-3 (Fragment) OS=Homo sapiens OX=9606 GN=CUL3 PE=1 SV=1 |
| 484 | sp\|P22102\|PUR2_HUMAN | 47.64 | 107767 | Trifunctional purine biosynthetic protein adenosine-3 OS=Homo sapiens OX=9606 GN=GART PE=1 SV=1 |
| 485 | sp\|Q9NW82\|WDR70_HUMAN | 47.49 | 73201 | WD repeat-containing protein 70 OS=Homo sapiens OX=9606 GN=WDR70 PE=1 SV=1 |
| 486 | tr\|D6RIW8\|D6RIW8_HUMAN | 47.49 | 43240 | WD repeat-containing protein 70 OS=Homo sapiens OX=9606 GN=WDR70 PE=1 SV=1 |
| 487 | tr\|B7Z6D5\|B7Z6D5_HUMAN | 47.35 | 86605 | Probable ATP-dependent RNA helicase DDX27 OS=Homo sapiens OX=9606 GN=DDX27 PE=1 SV=1 |
| 488 | sp\|Q96GQ7\|DDX27_HUMAN | 47.35 | 89835 | Probable ATP-dependent RNA helicase DDX27 OS=Homo sapiens OX=9606 GN=DDX27 PE=1 SV=2 |
| 489 | tr\|A0A1B0GWA9\|A0A1B0GWA9_HUMAN | 47.3 | 4709 | GTP-binding protein Di-Ras2 (Fragment) OS=Homo sapiens OX=9606 GN=DIRAS2 PE=1 SV=1 |
| 490 | tr\|A0A1B0GVC3\|A0A1B0GVC3_HUMAN | 47.3 | 12540 | GTP-binding protein Di-Ras2 (Fragment) OS=Homo sapiens OX=9606 GN=DIRAS2 PE=1 SV=1 |
| 491 | sp\|Q96HU8\|DIRA2_HUMAN | 47.3 | 22485 | GTP-binding protein Di-Ras2 OS=Homo sapiens OX=9606 GN=DIRAS2 PE=1 SV=1 |
| 492 | sp\|Q9UEY8\|ADDG_HUMAN | 46.66 | 79155 | Gamma-adducin OS=Homo sapiens OX=9606 GN=ADD3 PE=1 SV=1 |
| 493 | tr\|H0YGW7\|H0YGW7_HUMAN | 45.83 | 67669 | ATP-binding cassette sub-family F member 1 (Fragment) OS=Homo sapiens OX=9606 GN=ABCF1 PE=1 SV=1 |
| 494 | sp\|Q8NE71\|ABCF1_HUMAN | 45.83 | 95926 | ATP-binding cassette sub-family F member 1 OS=Homo sapiens OX=9606 GN=ABCF1 PE=1 SV=2 |
| 495 | tr\|A0A0G2JIC2\|A0A0G2JIC2_HUMAN | 45.83 | 27218 | ATP-binding cassette sub-family F member 1 OS=Homo sapiens OX=9606 GN=ABCF1 PE=1 SV=1 |
| 496 | sp\|O43592\|XPOT_HUMAN | 45.32 | 109964 | Exportin-T OS=Homo sapiens OX=9606 GN=XPOT PE=1 SV=2 |
| 497 | tr\|A0A1W2PNX8\|A0A1W2PNX8_HUMAN | 45.29 | 118395 | Protein unc-45 homolog A OS=Homo sapiens OX=9606 GN=UNC45A PE=1 SV=1 |
| 498 | sp\|Q9H3U1\|UN45A_HUMAN | 45.29 | 103077 | Protein unc-45 homolog A OS=Homo sapiens OX=9606 GN=UNC45A PE=1 SV=1 |
| 499 | tr\|H0Y8B3\|H0Y8B3_HUMAN | 43.84 | 58124 | Adenosylhomocysteinase (Fragment) OS=Homo sapiens OX=9606 GN=AHCYL2 PE=1 SV=1 |
| 500 | sp\|O43865\|SAHH2_HUMAN | 43.84 | 58952 | S-adenosylhomocysteine hydrolase-like protein 1 OS=Homo sapiens OX=9606 GN=AHCYL1 PE=1 SV=2 |
| 501 | sp\|Q96HN2\|SAHH3_HUMAN | 43.84 | 66721 | Adenosylhomocysteinase 3 OS=Homo sapiens OX=9606 GN=AHCYL2 PE=1 SV=1 |
| 502 | tr\|Q3BDU5\|Q3BDU5_HUMAN | 43.71 | 55638 | Prelamin-A/C OS=Homo sapiens OX=9606 GN=LMNA PE=1 SV=1 |
| 503 | sp\|P02545\|LMNA_HUMAN | 43.71 | 74140 | Prelamin-A/C OS=Homo sapiens OX=9606 GN=LMNA PE=1 SV=1 |
| 504 | tr\|Q5TCI8\|Q5TCI8_HUMAN | 43.71 | 55762 | Prelamin-A/C OS=Homo sapiens OX=9606 GN=LMNA PE=1 SV=1 |
| 505 | tr\|A0A0C4DGC5\|A0A0C4DGC5_HUMAN | 43.71 | 27536 | Prelamin-A/C (Fragment) OS=Homo sapiens OX=9606 GN=LMNA PE=1 SV=1 |
| 506 | sp\|Q13263\|TIF1B_HUMAN | 43.37 | 88550 | Transcription intermediary factor 1-beta OS=Homo sapiens OX=9606 GN=TRIM28 PE=1 SV=5 |
| 507 | tr\|M0R0K9\|M0R0K9_HUMAN | 43.37 | 48435 | Transcription intermediary factor 1-beta (Fragment) OS=Homo sapiens OX=9606 GN=TRIM28 PE=1 SV=1 |
| 508 | tr\|A6NJA2\|A6NJA2_HUMAN | 42.75 | 51087 | Ubiquitin carboxyl-terminal hydrolase 14 OS=Homo sapiens OX=9606 GN=USP14 PE=1 SV=2 |
| 509 | sp\|P54578\|UBP14_HUMAN | 42.75 | 56069 | Ubiquitin carboxyl-terminal hydrolase 14 OS=Homo sapiens OX=9606 GN=USP14 PE=1 SV=3 |
| 510 | tr\|E9PRV7\|E9PRV7_HUMAN | 42.61 | 46537 | G protein-coupled receptor kinase OS=Homo sapiens OX=9606 GN=GRK2 PE=1 SV=1 |
| 511 | sp\|P25098\|ARBK1_HUMAN | 42.61 | 79574 | Beta-adrenergic receptor kinase 1 OS=Homo sapiens OX=9606 GN=GRK2 PE=1 SV=2 |
| 512 | sp\|O00203\|AP3B1_HUMAN | 42.39 | 121320 | AP-3 complex subunit beta-1 OS=Homo sapiens OX=9606 GN=AP3B1 PE=1 SV=3 |
| 513 | tr\|H0Y873\|H0Y873_HUMAN | 42.12 | 43467 | Arf-GAP with GTPase  ANK repeat and PH domain-containing protein 3 (Fragment) OS=Homo sapiens OX=9606 GN=AGAP3 PE=1 SV=1 |
| 514 | sp\|Q96P47\|AGAP3_HUMAN | 42.12 | 95044 | Arf-GAP with GTPase  ANK repeat and PH domain-containing protein 3 OS=Homo sapiens OX=9606 GN=AGAP3 PE=1 SV=2 |
| 515 | tr\|A0A087WT27\|A0A087WT27_HUMAN | 42.04 | 49095 | Phosphoacetylglucosamine mutase OS=Homo sapiens OX=9606 GN=PGM3 PE=1 SV=1 |

| | | | | |
|---|---|---|---|---|
| 516 | tr\|J3KN95\|J3KN95_HUMAN | 42.04 | 51079 | Phosphoacetylglucosamine mutase OS=Homo sapiens OX=9606 GN=PGM3 PE=1 SV=1 |
| 517 | sp\|O95394\|AGM1_HUMAN | 42.04 | 59852 | Phosphoacetylglucosamine mutase OS=Homo sapiens OX=9606 GN=PGM3 PE=1 SV=1 |
| 518 | tr\|H0Y8I3\|H0Y8I3_HUMAN | 42.04 | 12434 | Phosphoacetylglucosamine mutase (Fragment) OS=Homo sapiens OX=9606 GN=PGM3 PE=1 SV=1 |
| 519 | tr\|H0Y987\|H0Y987_HUMAN | 42.04 | 21837 | Phosphoacetylglucosamine mutase (Fragment) OS=Homo sapiens OX=9606 GN=PGM3 PE=1 SV=1 |
| 520 | sp\|Q9Y5J1\|UTP18_HUMAN | 41.66 | 62004 | U3 small nucleolar RNA-associated protein 18 homolog OS=Homo sapiens OX=9606 GN=UTP18 PE=1 SV=3 |
| 521 | sp\|Q9HCC0\|MCCB_HUMAN | 41.54 | 61333 | Methylcrotonoyl-CoA carboxylase beta chain mitochondrial OS=Homo sapiens OX=9606 GN=MCCC2 PE=1 SV=1 |
| 522 | tr\|E9PLK3\|E9PLK3_HUMAN | 41.49 | 102988 | Aminopeptidase OS=Homo sapiens OX=9606 GN=NPEPPS PE=1 SV=1 |
| 523 | sp\|P55786\|PSA_HUMAN | 41.49 | 103276 | Puromycin-sensitive aminopeptidase OS=Homo sapiens OX=9606 GN=NPEPPS PE=1 SV=2 |
| 524 | tr\|H0YDG0\|H0YDG0_HUMAN | 41.49 | 19052 | Puromycin-sensitive aminopeptidase (Fragment) OS=Homo sapiens OX=9606 GN=NPEPPS PE=1 SV=1 |
| 525 | sp\|P13473\|LAMP2_HUMAN | 41.28 | 44961 | Lysosome-associated membrane glycoprotein 2 OS=Homo sapiens OX=9606 GN=LAMP2 PE=1 SV=2 |
| 526 | tr\|E9PD92\|E9PD92_HUMAN | 41.06 | 29503 | Glucose-6-phosphate 1-dehydrogenase (Fragment) OS=Homo sapiens OX=9606 GN=G6PD PE=1 SV=1 |
| 527 | tr\|E7EM57\|E7EM57_HUMAN | 41.06 | 36543 | Glucose-6-phosphate 1-dehydrogenase (Fragment) OS=Homo sapiens OX=9606 GN=G6PD PE=1 SV=1 |
| 528 | tr\|E7EUI8\|E7EUI8_HUMAN | 41.06 | 38451 | Glucose-6-phosphate 1-dehydrogenase (Fragment) OS=Homo sapiens OX=9606 GN=G6PD PE=1 SV=8 |
| 529 | sp\|P11413\|G6PD_HUMAN | 41.06 | 59257 | Glucose-6-phosphate 1-dehydrogenase OS=Homo sapiens OX=9606 GN=G6PD PE=1 SV=4 |
| 530 | sp\|P62805\|H4_HUMAN | 40.94 | 11367 | Histone H4 OS=Homo sapiens OX=9606 GN=HIST1H4A PE=1 SV=2 |
| 531 | tr\|A0A0B4J2G4\|A0A0B4J2G4_HUMAN | 40.89 | 86239 | Leucine-rich repeat-containing protein 41 OS=Homo sapiens OX=9606 GN=LRRC41 PE=1 SV=1 |
| 532 | sp\|Q15345\|LRC41_HUMAN | 40.89 | 88650 | Leucine-rich repeat-containing protein 41 OS=Homo sapiens OX=9606 GN=LRRC41 PE=1 SV=3 |
| 533 | tr\|A2A2G5\|A2A2G5_HUMAN | 40.7 | 21033 | Glutathione hydrolase 7 (Fragment) OS=Homo sapiens OX=9606 GN=GGT7 PE=1 SV=8 |
| 534 | sp\|Q9UJ14\|GGT7_HUMAN | 40.7 | 70467 | Glutathione hydrolase 7 OS=Homo sapiens OX=9606 GN=GGT7 PE=1 SV=2 |
| 535 | tr\|E5RHJ4\|E5RHJ4_HUMAN | 40.38 | 31090 | Cell cycle and apoptosis regulator protein 2 (Fragment) OS=Homo sapiens OX=9606 GN=CCAR2 PE=1 SV=1 |
| 536 | tr\|H0YB24\|H0YB24_HUMAN | 40.38 | 68932 | Cell cycle and apoptosis regulator protein 2 (Fragment) OS=Homo sapiens OX=9606 GN=CCAR2 PE=1 SV=1 |
| 537 | sp\|Q8N163\|CCAR2_HUMAN | 40.38 | 102902 | Cell cycle and apoptosis regulator protein 2 OS=Homo sapiens OX=9606 GN=CCAR2 PE=1 SV=2 |
| 538 | sp\|Q9NTK5\|OLA1_HUMAN | 39.34 | 44744 | Obg-like ATPase 1 OS=Homo sapiens OX=9606 GN=OLA1 PE=1 SV=2 |
| 539 | tr\|J3KQ32\|J3KQ32_HUMAN | 39.34 | 46938 | Obg-like ATPase 1 OS=Homo sapiens OX=9606 GN=OLA1 PE=1 SV=1 |
| 540 | tr\|H0YLC5\|H0YLC5_HUMAN | 38.55 | 16897 | POU domain class 2 transcription factor 3 (Fragment) OS=Homo sapiens OX=9606 GN=POU2F3 PE=4 SV=9 |
| 541 | tr\|U3KQ96\|U3KQ96_HUMAN | 38.55 | 28649 | POU domain class 2 transcription factor 2 (Fragment) OS=Homo sapiens OX=9606 GN=POU2F2 PE=1 SV=1 |
| 542 | tr\|U3KQN1\|U3KQN1_HUMAN | 38.55 | 33354 | POU domain class 2 transcription factor 2 (Fragment) OS=Homo sapiens OX=9606 GN=POU2F2 PE=1 SV=2 |
| 543 | tr\|H0YNW1\|H0YNW1_HUMAN | 38.55 | 45578 | POU domain protein OS=Homo sapiens OX=9606 GN=POU2F2 PE=1 SV=1 |
| 544 | sp\|Q9UKI9\|PO2F3_HUMAN | 38.55 | 47432 | POU domain class 2 transcription factor 3 OS=Homo sapiens OX=9606 GN=POU2F3 PE=2 SV=3 |
| 545 | sp\|P09086\|PO2F2_HUMAN | 38.55 | 51209 | POU domain class 2 transcription factor 2 OS=Homo sapiens OX=9606 GN=POU2F2 PE=1 SV=3 |
| 546 | tr\|H0YLL6\|H0YLL6_HUMAN | 38.55 | 51720 | POU domain protein OS=Homo sapiens OX=9606 GN=POU2F2 PE=1 SV=1 |
| 547 | tr\|A0A0C4DG88\|A0A0C4DG88_HUMAN | 38.55 | 58683 | POU domain protein (Fragment) OS=Homo sapiens OX=9606 GN=POU2F1 PE=1 SV=1 |
| 548 | tr\|B5ME60\|B5ME60_HUMAN | 38.55 | 60436 | POU domain protein OS=Homo sapiens OX=9606 GN=POU2F2 PE=1 SV=4 |
| 549 | tr\|A0A0A0MT46\|A0A0A0MT46_HUMAN | 38.55 | 61583 | POU domain protein (Fragment) OS=Homo sapiens OX=9606 GN=POU2F1 PE=1 SV=1 |
| 550 | tr\|H0YKT2\|H0YKT2_HUMAN | 38.55 | 65144 | POU domain protein (Fragment) OS=Homo sapiens OX=9606 GN=POU2F2 PE=1 SV=2 |
| 551 | sp\|P14859\|PO2F1_HUMAN | 38.55 | 76472 | POU domain class 2 transcription factor 1 OS=Homo sapiens OX=9606 GN=POU2F1 PE=1 SV=2 |
| 552 | tr\|H7BXY5\|H7BXY5_HUMAN | 38.49 | 68934 | Nexilin (Fragment) OS=Homo sapiens OX=9606 GN=NEXN PE=1 SV=1 |
| 553 | sp\|Q0ZGT2\|NEXN_HUMAN | 38.49 | 80658 | Nexilin OS=Homo sapiens OX=9606 GN=NEXN PE=1 SV=1 |
| 554 | sp\|Q13033\|STRN3_HUMAN | 38.17 | 87209 | Striatin-3 OS=Homo sapiens OX=9606 GN=STRN3 PE=1 SV=3 |
| 555 | sp\|Q5JRX3\|PREP_HUMAN | 38.13 | 117413 | Presequence protease mitochondrial OS=Homo sapiens OX=9606 GN=PITRM1 PE=1 SV=3 |
| 556 | tr\|A0A0A0MRX9\|A0A0A0MRX9_HUMAN | 38.13 | 67071 | Presequence protease mitochondrial OS=Homo sapiens OX=9606 GN=PITRM1 PE=1 SV=1 |
| 557 | tr\|B1APQ0\|B1APQ0_HUMAN | 38.13 | 20206 | Presequence protease mitochondrial (Fragment) OS=Homo sapiens OX=9606 GN=PITRM1 PE=1 SV=1 |
| 558 | sp\|P35580\|MYH10_HUMAN | 37.74 | 228997 | Myosin-10 OS=Homo sapiens OX=9606 GN=MYH10 PE=1 SV=3 |
| 559 | sp\|P35579\|MYH9_HUMAN | 37.74 | 226530 | Myosin-9 OS=Homo sapiens OX=9606 GN=MYH9 PE=1 SV=4 |
| 560 | sp\|Q14247\|SRC8_HUMAN | 37.52 | 61586 | Src substrate cortactin OS=Homo sapiens OX=9606 GN=CTTN PE=1 SV=2 |
| 561 | tr\|F5H0C8\|F5H0C8_HUMAN | 37.51 | 34762 | Gamma-enolase OS=Homo sapiens OX=9606 GN=ENO2 PE=1 SV=1 |
| 562 | sp\|P09104\|ENOG_HUMAN | 37.51 | 47269 | Gamma-enolase OS=Homo sapiens OX=9606 GN=ENO2 PE=1 SV=3 |
| 563 | tr\|F5H1C3\|F5H1C3_HUMAN | 37.51 | 11203 | Gamma-enolase (Fragment) OS=Homo sapiens OX=9606 GN=ENO2 PE=1 SV=1 |
| 564 | tr\|E5RI09\|E5RI09_HUMAN | 37.51 | 15798 | Beta-enolase (Fragment) OS=Homo sapiens OX=9606 GN=ENO3 PE=1 SV=1 |
| 565 | tr\|E5RG95\|E5RG95_HUMAN | 37.51 | 16457 | Beta-enolase (Fragment) OS=Homo sapiens OX=9606 GN=ENO3 PE=1 SV=1 |
| 566 | tr\|K7EPM1\|K7EPM1_HUMAN | 37.51 | 20922 | Beta-enolase (Fragment) OS=Homo sapiens OX=9606 GN=ENO3 PE=1 SV=1 |
| 567 | tr\|K7EKN2\|K7EKN2_HUMAN | 37.51 | 22514 | Beta-enolase (Fragment) OS=Homo sapiens OX=9606 GN=ENO3 PE=1 SV=1 |
| 568 | tr\|E5RGZ4\|E5RGZ4_HUMAN | 37.51 | 30403 | Beta-enolase (Fragment) OS=Homo sapiens OX=9606 GN=ENO3 PE=1 SV=1 |
| 569 | sp\|P13929\|ENOB_HUMAN | 37.51 | 46987 | Beta-enolase OS=Homo sapiens OX=9606 GN=ENO3 PE=1 SV=5 |
| 570 | tr\|G3V4N7\|G3V4N7_HUMAN | 37.46 | 24116 | Creatine kinase B-type (Fragment) OS=Homo sapiens OX=9606 GN=CKB PE=1 SV=1 |
| 571 | sp\|P12277\|KCRB_HUMAN | 37.46 | 42644 | Creatine kinase B-type OS=Homo sapiens OX=9606 GN=CKB PE=1 SV=1 |
| 572 | sp\|P85037\|FOXK1_HUMAN | 37.36 | 75457 | Forkhead box protein K1 OS=Homo sapiens OX=9606 GN=FOXK1 PE=1 SV=1 |
| 573 | tr\|A0A0U1RRM6\|A0A0U1RRM6_HUMAN | 37.2 | 87400 | Protein enabled homolog OS=Homo sapiens OX=9606 GN=ENAH PE=1 SV=1 |
| 574 | sp\|Q8N8S7\|ENAH_HUMAN | 37.2 | 66510 | Protein enabled homolog OS=Homo sapiens OX=9606 GN=ENAH PE=1 SV=2 |
| 575 | tr\|A0A087WZ13\|A0A087WZ13_HUMAN | 37.02 | 77844 | Ribonucleoprotein PTB-binding 1 OS=Homo sapiens OX=9606 GN=RAVER1 PE=1 SV=1 |
| 576 | tr\|E9PAU2\|E9PAU2_HUMAN | 37.02 | 79579 | Ribonucleoprotein PTB-binding 1 OS=Homo sapiens OX=9606 GN=RAVER1 PE=1 SV=1 |
| 577 | sp\|Q8IY67\|RAVR1_HUMAN | 37.02 | 63877 | Ribonucleoprotein PTB-binding 1 OS=Homo sapiens OX=9606 GN=RAVER1 PE=1 SV=1 |
| 578 | tr\|K7EKR9\|K7EKR9_HUMAN | 37.02 | 10950 | Ribonucleoprotein PTB-binding 1 (Fragment) OS=Homo sapiens OX=9606 GN=RAVER1 PE=1 SV=1 |
| 579 | sp\|P55771\|PAX9_HUMAN | 36.9 | 36310 | Paired box protein Pax-9 OS=Homo sapiens OX=9606 GN=PAX9 PE=1 SV=3 |
| 580 | tr\|G3V1N2\|G3V1N2_HUMAN | 36.73 | 11948 | HCG1745306 isoform CRA_a OS=Homo sapiens OX=9606 GN=HBA2 PE=1 SV=1 |
| 581 | tr\|A0A2R8Y7C0\|A0A2R8Y7C0_HUMAN | 36.73 | 13962 | Hemoglobin subunit alpha (Fragment) OS=Homo sapiens OX=9606 GN=HBA2 PE=1 SV=1 |
| 582 | sp\|P69905\|HBA_HUMAN | 36.73 | 15258 | Hemoglobin subunit alpha OS=Homo sapiens OX=9606 GN=HBA1 PE=1 SV=2 |
| 583 | sp\|P02008\|HBAZ_HUMAN | 36.73 | 15637 | Hemoglobin subunit zeta OS=Homo sapiens OX=9606 GN=HBZ PE=1 SV=2 |
| 584 | tr\|J3QLK5\|J3QLK5_HUMAN | 36.46 | 69288 | Serine/threonine-protein kinase tousled-like 2 OS=Homo sapiens OX=9606 GN=TLK2 PE=1 SV=1 |
| 585 | sp\|Q86UE8\|TLK2_HUMAN | 36.46 | 87661 | Serine/threonine-protein kinase tousled-like 2 OS=Homo sapiens OX=9606 GN=TLK2 PE=1 SV=2 |
| 586 | tr\|J3KRK0\|J3KRK0_HUMAN | 36.46 | 68833 | Serine/threonine-protein kinase tousled-like 2 (Fragment) OS=Homo sapiens OX=9606 GN=TLK2 PE=1 SV=1 |
| 587 | tr\|J3QQN4\|J3QQN4_HUMAN | 36.46 | 4173 | Serine/threonine-protein kinase tousled-like 2 (Fragment) OS=Homo sapiens OX=9606 GN=TLK2 PE=1 SV=1 |
| 588 | tr\|J3QS44\|J3QS44_HUMAN | 36.46 | 19253 | Serine/threonine-protein kinase tousled-like 2 (Fragment) OS=Homo sapiens OX=9606 GN=TLK2 PE=1 SV=1 |
| 589 | sp\|A6NNF4\|ZN726_HUMAN | 35.73 | 85539 | Zinc finger protein 726 OS=Homo sapiens OX=9606 GN=ZNF726 PE=1 SV=4 |
| 590 | tr\|A0A0B4J2G0\|A0A0B4J2G0_HUMAN | 35.73 | 98955 | Zinc finger protein 107 OS=Homo sapiens OX=9606 GN=ZNF107 PE=1 SV=1 |
| 591 | sp\|Q9BRH9\|ZN251_HUMAN | 35.73 | 75763 | Zinc finger protein 251 OS=Homo sapiens OX=9606 GN=ZNF251 PE=1 SV=4 |
| 592 | tr\|A0A0A0MR57\|A0A0A0MR57_HUMAN | 35.73 | 107729 | Zinc finger protein 836 OS=Homo sapiens OX=9606 GN=ZNF836 PE=1 SV=1 |
| 593 | sp\|Q6ZNA1\|ZN836_HUMAN | 35.73 | 107717 | Zinc finger protein 836 OS=Homo sapiens OX=9606 GN=ZNF836 PE=2 SV=2 |
| 594 | sp\|A6NN14\|ZN729_HUMAN | 35.73 | 145021 | Zinc finger protein 729 OS=Homo sapiens OX=9606 GN=ZNF729 PE=2 SV=4 |
| 595 | sp\|Q5JVG8\|ZN506_HUMAN | 35.73 | 51537 | Zinc finger protein 506 OS=Homo sapiens OX=9606 GN=ZNF506 PE=2 SV=2 |
| 596 | sp\|Q05481\|ZNF91_HUMAN | 35.73 | 137217 | Zinc finger protein 91 OS=Homo sapiens OX=9606 GN=ZNF91 PE=2 SV=2 |
| 597 | tr\|B9EG95\|B9EG95_HUMAN | 35.73 | 59133 | ZNF682 protein OS=Homo sapiens OX=9606 GN=ZNF682 PE=1 SV=1 |
| 598 | tr\|A0A0A0MTN5\|A0A0A0MTN5_HUMAN | 35.73 | 55603 | Zinc finger protein 100 OS=Homo sapiens OX=9606 GN=ZNF100 PE=1 SV=1 |
| 599 | sp\|Q8IYN0\|ZN100_HUMAN | 35.73 | 62745 | Zinc finger protein 100 OS=Homo sapiens OX=9606 GN=ZNF100 PE=2 SV=2 |
| 600 | sp\|Q96N38\|ZN714_HUMAN | 35.73 | 63883 | Zinc finger protein 714 OS=Homo sapiens OX=9606 GN=ZNF714 PE=2 SV=3 |
| 601 | tr\|A0A087WU35\|A0A087WU35_HUMAN | 35.73 | 63998 | Zinc finger protein 714 OS=Homo sapiens OX=9606 GN=ZNF714 PE=1 SV=1 |
| 602 | tr\|A0A075B7G6\|A0A075B7G6_HUMAN | 35.73 | 65478 | Zinc finger protein 100 OS=Homo sapiens OX=9606 GN=ZNF100 PE=1 SV=2 |
| 603 | sp\|Q9NV72\|ZN701_HUMAN | 35.73 | 60903 | Zinc finger protein 701 OS=Homo sapiens OX=9606 GN=ZNF701 PE=1 SV=3 |
| 604 | sp\|Q6ZN06\|ZN813_HUMAN | 35.73 | 71721 | Zinc finger protein 813 OS=Homo sapiens OX=9606 GN=ZNF813 PE=2 SV=2 |
| 605 | sp\|P17035\|ZNF28_HUMAN | 35.73 | 83659 | Zinc finger protein 28 OS=Homo sapiens OX=9606 GN=ZNF28 PE=1 SV=5 |
| 606 | sp\|P17040\|ZSC20_HUMAN | 35.73 | 117541 | Zinc finger and SCAN domain-containing protein 20 OS=Homo sapiens OX=9606 GN=ZSCAN20 PE=1 SV=3 |
| 607 | sp\|Q96CX3\|ZN501_HUMAN | 35.73 | 31179 | Zinc finger protein 501 OS=Homo sapiens OX=9606 GN=ZNF501 PE=2 SV=2 |
| 608 | sp\|Q8N8J6\|ZN615_HUMAN | 35.73 | 83739 | Zinc finger protein 615 OS=Homo sapiens OX=9606 GN=ZNF615 PE=2 SV=2 |
| 609 | sp\|P52744\|ZN138_HUMAN | 35.73 | 30591 | Zinc finger protein 138 OS=Homo sapiens OX=9606 GN=ZNF138 PE=1 SV=2 |
| 610 | tr\|E7EWC5\|E7EWC5_HUMAN | 35.73 | 33686 | Zinc finger protein 138 OS=Homo sapiens OX=9606 GN=ZNF138 PE=4 SV=1 |
| 611 | tr\|J3QSS9\|J3QSS9_HUMAN | 35.73 | 37395 | Zinc finger protein 138 OS=Homo sapiens OX=9606 GN=ZNF138 PE=4 SV=1 |
| 612 | sp\|A6NP11\|ZN716_HUMAN | 35.73 | 57006 | Zinc finger protein 716 OS=Homo sapiens OX=9606 GN=ZNF716 PE=2 SV=4 |
| 613 | sp\|Q9Y2Q1\|ZN257_HUMAN | 35.73 | 65780 | Zinc finger protein 257 OS=Homo sapiens OX=9606 GN=ZNF257 PE=2 SV=2 |
| 614 | sp\|P35789\|ZNF93_HUMAN | 35.73 | 70971 | Zinc finger protein 93 OS=Homo sapiens OX=9606 GN=ZNF93 PE=2 SV=4 |
| 615 | sp\|Q5JNZ3\|ZN311_HUMAN | 35.73 | 76322 | Zinc finger protein 311 OS=Homo sapiens OX=9606 GN=ZNF311 PE=2 SV=2 |
| 616 | sp\|Q3SXZ3\|ZN718_HUMAN | 35.73 | 55359 | Zinc finger protein 718 OS=Homo sapiens OX=9606 GN=ZNF718 PE=2 SV=2 |
| 617 | sp\|Q8N988\|ZN557_HUMAN | 35.73 | 48627 | Zinc finger protein 557 OS=Homo sapiens OX=9606 GN=ZNF557 PE=2 SV=2 |
| 618 | sp\|Q9UEG4\|ZN629_HUMAN | 35.73 | 96620 | Zinc finger protein 629 OS=Homo sapiens OX=9606 GN=ZNF629 PE=1 SV=2 |
| 619 | tr\|A0A087WUV0\|A0A087WUV0_HUMAN | 35.73 | 59574 | Uncharacterized protein OS=Homo sapiens OX=9606 PE=1 SV=1 |
| 620 | sp\|Q5HY98\|ZN766_HUMAN | 35.73 | 54507 | Zinc finger protein 766 OS=Homo sapiens OX=9606 GN=ZNF766 PE=1 SV=1 |
| 621 | sp\|Q14590\|ZN235_HUMAN | 35.73 | 83977 | Zinc finger protein 235 OS=Homo sapiens OX=9606 GN=ZNF235 PE=2 SV=3 |
| 622 | sp\|Q9UL36\|ZN236_HUMAN | 35.73 | 203702 | Zinc finger protein 236 OS=Homo sapiens OX=9606 GN=ZNF236 PE=2 SV=2 |
| 623 | tr\|J9JID5\|J9JID5_HUMAN | 35.73 | 203845 | Zinc finger protein 236 OS=Homo sapiens OX=9606 GN=ZNF236 PE=1 SV=1 |
| 624 | sp\|Q8N8C0\|ZN781_HUMAN | 35.73 | 41526 | Zinc finger protein 781 OS=Homo sapiens OX=9606 GN=ZNF781 PE=2 SV=1 |
| 625 | sp\|O95600\|KLF8_HUMAN | 35.73 | 39314 | Krueppel-like factor 8 OS=Homo sapiens OX=9606 GN=KLF8 PE=1 SV=2 |
| 626 | tr\|G3V1M0\|G3V1M0_HUMAN | 35.73 | 49339 | Zinc finger and SCAN domain-containing protein 21 OS=Homo sapiens OX=9606 GN=ZSCAN21 PE=1 SV=2 |
| 627 | sp\|P10073\|ZSC22_HUMAN | 35.73 | 54561 | Zinc finger and SCAN domain-containing protein 22 OS=Homo sapiens OX=9606 GN=ZSCAN22 PE=1 SV=2 |
| 628 | sp\|P51815\|ZN75D_HUMAN | 35.73 | 59298 | Zinc finger protein 75D OS=Homo sapiens OX=9606 GN=ZNF75D PE=2 SV=2 |
| 629 | sp\|Q8NEM1\|ZN680_HUMAN | 35.73 | 61798 | Zinc finger protein 680 OS=Homo sapiens OX=9606 GN=ZNF680 PE=1 SV=2 |
| 630 | tr\|A0A0C4DG42\|A0A0C4DG42_HUMAN | 35.73 | 61708 | Zinc finger protein 34 OS=Homo sapiens OX=9606 GN=ZNF34 PE=1 SV=1 |
| 631 | sp\|Q8TB69\|ZN519_HUMAN | 35.73 | 63020 | Zinc finger protein 519 OS=Homo sapiens OX=9606 GN=ZNF519 PE=2 SV=2 |
| 632 | sp\|Q6ZMS4\|ZN852_HUMAN | 35.73 | 62084 | Zinc finger protein 852 OS=Homo sapiens OX=9606 GN=ZNF852 PE=1 SV=4 |
| 633 | sp\|B4DU55\|ZN879_HUMAN | 35.73 | 64577 | Zinc finger protein 879 OS=Homo sapiens OX=9606 GN=ZNF879 PE=2 SV=2 |
| 634 | sp\|Q6PDB4\|ZN880_HUMAN | 35.73 | 66762 | Zinc finger protein 880 OS=Homo sapiens OX=9606 GN=ZNF880 PE=2 SV=2 |
| 635 | sp\|Q9P2F9\|ZN319_HUMAN | 35.73 | 65547 | Zinc finger protein 319 OS=Homo sapiens OX=9606 GN=ZNF319 PE=1 SV=2 |
| 636 | tr\|A0A087WV98\|A0A087WV98_HUMAN | 35.73 | 67996 | Zinc finger protein 429 OS=Homo sapiens OX=9606 GN=ZNF429 PE=4 SV=1 |
| 637 | tr\|K7EP39\|K7EP39_HUMAN | 35.73 | 68433 | Zinc finger protein 570 OS=Homo sapiens OX=9606 GN=ZNF570 PE=1 SV=1 |
| 638 | sp\|Q08ER8\|ZN543_HUMAN | 35.73 | 68404 | Zinc finger protein 543 OS=Homo sapiens OX=9606 GN=ZNF543 PE=1 SV=3 |
| 639 | sp\|Q7Z3V5\|ZN571_HUMAN | 35.73 | 70793 | Zinc finger protein 571 OS=Homo sapiens OX=9606 GN=ZNF571 PE=2 SV=3 |
| 640 | sp\|Q16587\|ZNF74_HUMAN | 35.73 | 72207 | Zinc finger protein 74 OS=Homo sapiens OX=9606 GN=ZNF74 PE=1 SV=3 |
| 641 | sp\|Q3ZCX4\|ZN568_HUMAN | 35.73 | 74369 | Zinc finger protein 568 OS=Homo sapiens OX=9606 GN=ZNF568 PE=2 SV=2 |
| 642 | sp\|Q8NDQ6\|ZN540_HUMAN | 35.73 | 77094 | Zinc finger protein 540 OS=Homo sapiens OX=9606 GN=ZNF540 PE=1 SV=1 |
| 643 | sp\|O43361\|ZN749_HUMAN | 35.73 | 90364 | Zinc finger protein 749 OS=Homo sapiens OX=9606 GN=ZNF749 PE=1 SV=2 |
| 644 | sp\|Q08AN1\|ZN616_HUMAN | 35.73 | 90273 | Zinc finger protein 616 OS=Homo sapiens OX=9606 GN=ZNF616 PE=2 SV=2 |

| | | | | |
|---|---|---|---|---|
| 645 | sp\|Q8WXB4\|ZN606_HUMAN | 35.73 | 91812 | Zinc finger protein 606 OS=Homo sapiens OX=9606 GN=ZNF606 PE=2 SV=1 |
| 646 | sp\|Q4V348\|Z658B_HUMAN | 35.73 | 94331 | Zinc finger protein 658B OS=Homo sapiens OX=9606 GN=ZNF658B PE=2 SV=1 |
| 647 | sp\|Q9Y6R6\|Z780B_HUMAN | 35.73 | 96807 | Zinc finger protein 780B OS=Homo sapiens OX=9606 GN=ZNF780B PE=2 SV=1 |
| 648 | sp\|Q8NB50\|ZFP62_HUMAN | 35.73 | 102511 | Zinc finger protein 62 homolog OS=Homo sapiens OX=9606 GN=ZFP62 PE=1 SV=3 |
| 649 | sp\|Q96BY6\|DOC10_HUMAN | 35.66 | 249529 | Dedicator of cytokinesis protein 10 OS=Homo sapiens OX=9606 GN=DOCK10 PE=1 SV=3 |
| 650 | tr\|A0A2R8YD85\|A0A2R8YD85_HUMAN | 35.66 | 251313 | Dedicator of cytokinesis protein 10 OS=Homo sapiens OX=9606 GN=DOCK10 PE=1 SV=1 |
| 651 | sp\|O14974\|MYPT1_HUMAN | 35.62 | 115281 | Protein phosphatase 1 regulatory subunit 12A OS=Homo sapiens OX=9606 GN=PPP1R12A PE=1 SV=1 |
| 652 | tr\|F8VZN8\|F8VZN8_HUMAN | 35.62 | 76533 | Protein phosphatase 1 regulatory subunit 12A (Fragment) OS=Homo sapiens OX=9606 GN=PPP1R12A PE=1 SV=1 |
| 653 | tr\|H0YIS4\|H0YIS4_HUMAN | 35.62 | 33152 | Protein phosphatase 1 regulatory subunit 12A (Fragment) OS=Homo sapiens OX=9606 GN=PPP1R12A PE=1 SV=1 |
| 654 | tr\|H0YHI8\|H0YHI8_HUMAN | 35.62 | 40623 | Protein phosphatase 1 regulatory subunit 12A (Fragment) OS=Homo sapiens OX=9606 GN=PPP1R12A PE=1 SV=1 |
| 655 | tr\|A0A3B3ISH4\|A0A3B3ISH4_HUMAN | 35.62 | 40958 | Protein phosphatase 1 regulatory subunit 12A (Fragment) OS=Homo sapiens OX=9606 GN=PPP1R12A PE=1 SV=1 |
| 656 | sp\|P54577\|SYYC_HUMAN | 35.44 | 59144 | Tyrosine--tRNA ligase cytoplasmic OS=Homo sapiens OX=9606 GN=YARS PE=1 SV=4 |
| 657 | tr\|A0A0C4DGZ5\|A0A0C4DGZ5_HUMAN | 35.44 | 43905 | Tyrosine--tRNA ligase OS=Homo sapiens OX=9606 GN=YARS PE=1 SV=1 |
| 658 | sp\|Q5TCS8\|KAD9_HUMAN | 34.92 | 221411 | Adenylate kinase 9 OS=Homo sapiens OX=9606 GN=AK9 PE=1 SV=2 |
| 659 | tr\|G3V213\|G3V213_HUMAN | 34.92 | 14325 | Adenylate kinase 2 isoform CRA_a OS=Homo sapiens OX=9606 GN=AK2 PE=1 SV=1 |
| 660 | tr\|F8VZG5\|F8VZG5_HUMAN | 34.92 | 17499 | Adenylate kinase 2 mitochondrial OS=Homo sapiens OX=9606 GN=AK2 PE=1 SV=1 |
| 661 | tr\|F8VY04\|F8VY04_HUMAN | 34.92 | 21226 | Adenylate kinase 2 mitochondrial OS=Homo sapiens OX=9606 GN=AK2 PE=1 SV=1 |
| 662 | tr\|H7C505\|H7C505_HUMAN | 34.92 | 25370 | Adenylate kinase 9 (Fragment) OS=Homo sapiens OX=9606 GN=AK9 PE=1 SV=1 |
| 663 | tr\|F8W1A4\|F8W1A4_HUMAN | 34.92 | 25631 | Adenylate kinase 2 mitochondrial OS=Homo sapiens OX=9606 GN=AK2 PE=1 SV=1 |
| 664 | sp\|P54819\|KAD2_HUMAN | 34.92 | 26478 | Adenylate kinase 2 mitochondrial OS=Homo sapiens OX=9606 GN=AK2 PE=1 SV=2 |
| 665 | sp\|Q96I59\|SYNM_HUMAN | 34.85 | 54090 | Probable asparagine--tRNA ligase mitochondrial OS=Homo sapiens OX=9606 GN=NARS2 PE=1 SV=3 |
| 666 | tr\|X6RGJ2\|X6RGJ2_HUMAN | 34.66 | 36515 | Heterochromatin protein 1-binding protein 3 (Fragment) OS=Homo sapiens OX=9606 GN=HP1BP3 PE=1 SV=1 |
| 667 | sp\|Q5SSJ5\|HP1B3_HUMAN | 34.66 | 61207 | Heterochromatin protein 1-binding protein 3 OS=Homo sapiens OX=9606 GN=HP1BP3 PE=1 SV=1 |
| 668 | tr\|B0QZK9\|B0QZK9_HUMAN | 34.66 | 8150 | Heterochromatin protein 1-binding protein 3 (Fragment) OS=Homo sapiens OX=9606 GN=HP1BP3 PE=1 SV=8 |
| 669 | tr\|B0QZK8\|B0QZK8_HUMAN | 34.66 | 13684 | Heterochromatin protein 1-binding protein 3 (Fragment) OS=Homo sapiens OX=9606 GN=HP1BP3 PE=1 SV=1 |
| 670 | tr\|A0A3B3ITB4\|A0A3B3ITB4_HUMAN | 34.55 | 18612 | GTP-binding protein Rit2 OS=Homo sapiens OX=9606 GN=RIT2 PE=1 SV=1 |
| 671 | tr\|K7EMR8\|K7EMR8_HUMAN | 34.55 | 19100 | GTP-binding protein Rit2 OS=Homo sapiens OX=9606 GN=RIT2 PE=1 SV=1 |
| 672 | sp\|Q99578\|RIT2_HUMAN | 34.55 | 24668 | GTP-binding protein Rit2 OS=Homo sapiens OX=9606 GN=RIT2 PE=1 SV=1 |
| 673 | sp\|P53618\|COPB_HUMAN | 34.05 | 107142 | Coatomer subunit beta OS=Homo sapiens OX=9606 GN=COPB1 PE=1 SV=3 |
| 674 | tr\|E7EPB3\|E7EPB3_HUMAN | 34.04 | 14558 | 60S ribosomal protein L14 OS=Homo sapiens OX=9606 GN=RPL14 PE=1 SV=1 |
| 675 | sp\|P50914\|RL14_HUMAN | 34.04 | 23432 | 60S ribosomal protein L14 OS=Homo sapiens OX=9606 GN=RPL14 PE=1 SV=4 |
| 676 | sp\|Q2NL82\|TSR1_HUMAN | 34 | 91810 | Pre-rRNA-processing protein TSR1 homolog OS=Homo sapiens OX=9606 GN=TSR1 PE=1 SV=1 |
| 677 | tr\|H0Y6U5\|H0Y6U5_HUMAN | 33.97 | 102922 | Condensin-2 complex subunit G2 (Fragment) OS=Homo sapiens OX=9606 GN=NCAPG2 PE=1 SV=1 |
| 678 | sp\|Q86XI2\|CNDG2_HUMAN | 33.97 | 130960 | Condensin-2 complex subunit G2 OS=Homo sapiens OX=9606 GN=NCAPG2 PE=1 SV=1 |
| 679 | sp\|P22735\|TGM1_HUMAN | 33.93 | 89787 | Protein-glutamine gamma-glutamyltransferase K OS=Homo sapiens OX=9606 GN=TGM1 PE=1 SV=4 |
| 680 | tr\|C9JFE4\|C9JFE4_HUMAN | 33.84 | 53372 | COP9 signalosome complex subunit 1 OS=Homo sapiens OX=9606 GN=GPS1 PE=1 SV=2 |
| 681 | tr\|A0A096LP07\|A0A096LP07_HUMAN | 33.84 | 54965 | COP9 signalosome complex subunit 1 OS=Homo sapiens OX=9606 GN=GPS1 PE=1 SV=1 |
| 682 | tr\|A0A096LPJ3\|A0A096LPJ3_HUMAN | 33.84 | 55408 | COP9 signalosome complex subunit 1 OS=Homo sapiens OX=9606 GN=GPS1 PE=1 SV=1 |
| 683 | sp\|Q13098\|CSN1_HUMAN | 33.84 | 55537 | COP9 signalosome complex subunit 1 OS=Homo sapiens OX=9606 GN=GPS1 PE=1 SV=4 |
| 684 | tr\|A8K070\|A8K070_HUMAN | 33.84 | 58922 | COP9 signalosome complex subunit 1 OS=Homo sapiens OX=9606 GN=GPS1 PE=1 SV=1 |
| 685 | tr\|J3QQX0\|J3QQX0_HUMAN | 33.84 | 8709 | COP9 signalosome complex subunit 1 (Fragment) OS=Homo sapiens OX=9606 GN=GPS1 PE=1 SV=1 |
| 686 | sp\|Q14554\|PDIA5_HUMAN | 33.72 | 59594 | Protein disulfide-isomerase A5 OS=Homo sapiens OX=9606 GN=PDIA5 PE=1 SV=1 |
| 687 | sp\|Q6VY07\|PACS1_HUMAN | 33.63 | 104898 | Phosphofurin acidic cluster sorting protein 1 OS=Homo sapiens OX=9606 GN=PACS1 PE=1 SV=2 |
| 688 | sp\|P46087\|NOP2_HUMAN | 33.29 | 89302 | Probable 28S rRNA (cytosine(4447)-C(5))-methyltransferase OS=Homo sapiens OX=9606 GN=NOP2 PE=1 SV=2 |
| 689 | sp\|P56192\|SYMC_HUMAN | 33.22 | 101116 | Methionine--tRNA ligase cytoplasmic OS=Homo sapiens OX=9606 GN=MARS PE=1 SV=2 |
| 690 | sp\|Q6NSJ5\|LRC8E_HUMAN | 33.16 | 90247 | Volume-regulated anion channel subunit LRRC8E OS=Homo sapiens OX=9606 GN=LRRC8E PE=1 SV=2 |
| 691 | sp\|O43719\|HTSF1_HUMAN | 32.93 | 85853 | HIV Tat-specific factor 1 OS=Homo sapiens OX=9606 GN=HTATSF1 PE=1 SV=1 |
| 692 | tr\|F6VX93\|F6VX93_HUMAN | 32.93 | 62167 | Vacuolar protein sorting-associated protein 53 homolog OS=Homo sapiens OX=9606 GN=VPS53 PE=1 SV=1 |
| 693 | sp\|Q5VIR6\|VPS53_HUMAN | 32.93 | 79653 | Vacuolar protein sorting-associated protein 53 homolog OS=Homo sapiens OX=9606 GN=VPS53 PE=1 SV=1 |
| 694 | tr\|I3L184\|I3L184_HUMAN | 32.93 | 27162 | Vacuolar protein sorting-associated protein 53 homolog (Fragment) OS=Homo sapiens OX=9606 GN=VPS53 PE=1 SV=1 |
| 695 | sp\|Q86Z14\|KLOTB_HUMAN | 32.89 | 119808 | Beta-klotho OS=Homo sapiens OX=9606 GN=KLB PE=1 SV=1 |
| 696 | tr\|A0A087WYK2\|A0A087WYK2_HUMAN | 32.79 | 198280 | Serine/threonine-protein kinase WNK3 OS=Homo sapiens OX=9606 GN=WNK3 PE=1 SV=1 |
| 697 | sp\|Q9BYP7\|WNK3_HUMAN | 32.79 | 198414 | Serine/threonine-protein kinase WNK3 OS=Homo sapiens OX=9606 GN=WNK3 PE=1 SV=3 |
| 698 | tr\|F8W9F9\|F8W9F9_HUMAN | 32.79 | 234378 | Serine/threonine-protein kinase WNK2 OS=Homo sapiens OX=9606 GN=WNK2 PE=1 SV=1 |
| 699 | sp\|Q9Y3S1\|WNK2_HUMAN | 32.79 | 242673 | Serine/threonine-protein kinase WNK2 OS=Homo sapiens OX=9606 GN=WNK2 PE=1 SV=4 |
| 700 | sp\|Q9NVM9\|INT13_HUMAN | 32.78 | 80225 | Integrator complex subunit 13 OS=Homo sapiens OX=9606 GN=INTS13 PE=1 SV=2 |
| 701 | sp\|P10828\|THB_HUMAN | 32.58 | 52788 | Thyroid hormone receptor beta OS=Homo sapiens OX=9606 GN=THRB PE=1 SV=2 |
| 702 | sp\|Q86TJ2\|TAD2B_HUMAN | 32.58 | 48470 | Transcriptional adapter 2-beta OS=Homo sapiens OX=9606 GN=TADA2B PE=1 SV=2 |
| 703 | sp\|P10827\|THA_HUMAN | 32.58 | 54816 | Thyroid hormone receptor alpha OS=Homo sapiens OX=9606 GN=THRA PE=1 SV=1 |
| 704 | tr\|G3XAL0\|G3XAL0_HUMAN | 32.58 | 24595 | Malate dehydrogenase OS=Homo sapiens OX=9606 GN=MDH2 PE=1 SV=1 |
| 705 | sp\|P40926\|MDHM_HUMAN | 32.58 | 35503 | Malate dehydrogenase mitochondrial OS=Homo sapiens OX=9606 GN=MDH2 PE=1 SV=3 |
| 706 | sp\|Q9NQX3\|GEPH_HUMAN | 32.54 | 79748 | Gephyrin OS=Homo sapiens OX=9606 GN=GPHN PE=1 SV=1 |
| 707 | tr\|H0YJ30\|H0YJ30_HUMAN | 32.54 | 30112 | Gephyrin (Fragment) OS=Homo sapiens OX=9606 GN=GPHN PE=1 SV=1 |
| 708 | sp\|Q8N3K9\|CMYA5_HUMAN | 32.52 | 449215 | Cardiomyopathy-associated protein 5 OS=Homo sapiens OX=9606 GN=CMYA5 PE=1 SV=3 |
| 709 | sp\|Q9NXC5\|MIO_HUMAN | 32.36 | 98584 | GATOR complex protein MIOS OS=Homo sapiens OX=9606 GN=MIOS PE=1 SV=2 |
| 710 | sp\|P31327\|CPSM_HUMAN | 32.2 | 164939 | Carbamoyl-phosphate synthase [ammonia] mitochondrial OS=Homo sapiens OX=9606 GN=CPS1 PE=1 SV=2 |
| 711 | sp\|Q00266\|METK1_HUMAN | 31.99 | 43648 | S-adenosylmethionine synthase isoform type-1 OS=Homo sapiens OX=9606 GN=MAT1A PE=1 SV=2 |
| 712 | sp\|P31153\|METK2_HUMAN | 31.99 | 43661 | S-adenosylmethionine synthase isoform type-2 OS=Homo sapiens OX=9606 GN=MAT2A PE=1 SV=1 |
| 713 | tr\|A0A3B3IRQ9\|A0A3B3IRQ9_HUMAN | 31.88 | 128389 | Double-stranded RNA-specific adenosine deaminase (Fragment) OS=Homo sapiens OX=9606 GN=ADAR PE=1 SV=1 |
| 714 | tr\|H0YCK3\|H0YCK3_HUMAN | 31.88 | 132647 | Double-stranded RNA-specific adenosine deaminase (Fragment) OS=Homo sapiens OX=9606 GN=ADAR PE=1 SV=1 |
| 715 | sp\|P55265\|DSRAD_HUMAN | 31.88 | 136066 | Double-stranded RNA-specific adenosine deaminase OS=Homo sapiens OX=9606 GN=ADAR PE=1 SV=4 |
| 716 | tr\|A0A3B3ISX1\|A0A3B3ISX1_HUMAN | 31.88 | 78042 | Double-stranded RNA-specific adenosine deaminase (Fragment) OS=Homo sapiens OX=9606 GN=ADAR PE=1 SV=1 |
| 717 | tr\|A0A087X253\|A0A087X253_HUMAN | 31.82 | 101333 | AP complex subunit beta OS=Homo sapiens OX=9606 GN=AP2B1 PE=1 SV=1 |
| 718 | sp\|P63010\|AP2B1_HUMAN | 31.82 | 104553 | AP-2 complex subunit beta OS=Homo sapiens OX=9606 GN=AP2B1 PE=1 SV=1 |
| 719 | tr\|C9J1E7\|C9J1E7_HUMAN | 31.82 | 65315 | AP-1 complex subunit beta-1 (Fragment) OS=Homo sapiens OX=9606 GN=AP1B1 PE=1 SV=1 |
| 720 | tr\|A0A087WYD1\|A0A087WYD1_HUMAN | 31.82 | 70917 | AP-2 complex subunit beta (Fragment) OS=Homo sapiens OX=9606 GN=AP2B1 PE=1 SV=1 |
| 721 | tr\|A0A087WU93\|A0A087WU93_HUMAN | 31.82 | 74137 | AP-2 complex subunit beta (Fragment) OS=Homo sapiens OX=9606 GN=AP2B1 PE=1 SV=1 |
| 722 | tr\|A0A087WZQ6\|A0A087WZQ6_HUMAN | 31.82 | 75276 | AP-2 complex subunit beta (Fragment) OS=Homo sapiens OX=9606 GN=AP2B1 PE=1 SV=1 |
| 723 | sp\|Q10567\|AP1B1_HUMAN | 31.82 | 104637 | AP-1 complex subunit beta-1 OS=Homo sapiens OX=9606 GN=AP1B1 PE=1 SV=2 |
| 724 | sp\|Q9BX84\|TRPM6_HUMAN | 31.73 | 231706 | Transient receptor potential cation channel subfamily M member 6 OS=Homo sapiens OX=9606 GN=TRPM6 PE=1 SV=2 |
| 725 | tr\|H3BUF6\|H3BUF6_HUMAN | 31.36 | 112714 | Ataxin-2-like protein OS=Homo sapiens OX=9606 GN=ATXN2L PE=1 SV=1 |
| 726 | sp\|Q8WWM7\|ATX2L_HUMAN | 31.36 | 113374 | Ataxin-2-like protein OS=Homo sapiens OX=9606 GN=ATXN2L PE=1 SV=2 |
| 727 | tr\|H3BUE3\|H3BUE3_HUMAN | 31.36 | 23513 | Ataxin-2-like protein (Fragment) OS=Homo sapiens OX=9606 GN=ATXN2L PE=1 SV=1 |
| 728 | tr\|H3BSK9\|H3BSK9_HUMAN | 31.36 | 37138 | Ataxin-2-like protein (Fragment) OS=Homo sapiens OX=9606 GN=ATXN2L PE=1 SV=1 |
| 729 | sp\|P10155\|RO60_HUMAN | 30.89 | 60671 | 60 kDa SS-A/Ro ribonucleoprotein OS=Homo sapiens OX=9606 GN=TROVE2 PE=1 SV=2 |
| 730 | tr\|J3KMX1\|J3KMX1_HUMAN | 30.81 | 78890 | Nuclear pore complex protein Nup88 (Fragment) OS=Homo sapiens OX=9606 GN=NUP88 PE=1 SV=1 |
| 731 | sp\|Q99567\|NUP88_HUMAN | 30.81 | 83542 | Nuclear pore complex protein Nup88 OS=Homo sapiens OX=9606 GN=NUP88 PE=1 SV=2 |
| 732 | sp\|Q99661\|KIF2C_HUMAN | 30.76 | 81313 | Kinesin-like protein KIF2C OS=Homo sapiens OX=9606 GN=KIF2C PE=1 SV=2 |
| 733 | sp\|O60264\|SMCA5_HUMAN | 30.75 | 121905 | SWI/SNF-related matrix-associated actin-dependent regulator of chromatin subfamily A member 5 OS=Homo sapiens OX=9606 GN=SMARCA5 PE=1 S |
| 734 | sp\|P28370\|SMCA1_HUMAN | 30.75 | 122605 | Probable global transcription activator SNF2L1 OS=Homo sapiens OX=9606 GN=SMARCA1 PE=1 SV=2 |
| 735 | tr\|A0A0A0MRP6\|A0A0A0MRP6_HUMAN | 30.75 | 122864 | Probable global transcription activator SNF2L1 OS=Homo sapiens OX=9606 GN=SMARCA1 PE=1 SV=1 |
| 736 | tr\|B7ZLQ5\|B7ZLQ5_HUMAN | 30.75 | 124327 | Probable global transcription activator SNF2L1 OS=Homo sapiens OX=9606 GN=SMARCA1 PE=1 SV=1 |
| 737 | sp\|P04040\|CATA_HUMAN | 30.7 | 59756 | Catalase OS=Homo sapiens OX=9606 GN=CAT PE=1 SV=3 |
| 738 | sp\|P30419\|NMT1_HUMAN | 30.66 | 56806 | Glycylpeptide N-tetradecanoyltransferase 1 OS=Homo sapiens OX=9606 GN=NMT1 PE=1 SV=2 |
| 739 | tr\|M0QZN2\|M0QZN2_HUMAN | 30.24 | 14763 | 40S ribosomal protein S5 OS=Homo sapiens OX=9606 GN=RPS5 PE=1 SV=1 |
| 740 | tr\|M0R0F0\|M0R0F0_HUMAN | 30.24 | 22391 | 40S ribosomal protein S5 (Fragment) OS=Homo sapiens OX=9606 GN=RPS5 PE=1 SV=1 |
| 741 | sp\|P46782\|RS5_HUMAN | 30.24 | 22876 | 40S ribosomal protein S5 OS=Homo sapiens OX=9606 GN=RPS5 PE=1 SV=4 |
| 742 | tr\|M0R0R2\|M0R0R2_HUMAN | 30.24 | 25333 | 40S ribosomal protein S5 OS=Homo sapiens OX=9606 GN=RPS5 PE=1 SV=1 |
| 743 | sp\|O14980\|XPO1_HUMAN | 29.93 | 123386 | Exportin-1 OS=Homo sapiens OX=9606 GN=XPO1 PE=1 SV=1 |
| 744 | sp\|P06280\|AGAL_HUMAN | 29.89 | 48767 | Alpha-galactosidase A OS=Homo sapiens OX=9606 GN=GLA PE=1 SV=1 |
| 745 | tr\|A0A3B3IUC4\|A0A3B3IUC4_HUMAN | 29.89 | 53284 | Alpha-galactosidase OS=Homo sapiens OX=9606 GN=GLA PE=1 SV=1 |
| 746 | tr\|A0A1C7CYX1\|A0A1C7CYX1_HUMAN | 29.77 | 120733 | ELM2 and SANT domain-containing protein 1 OS=Homo sapiens OX=9606 GN=ELMSAN1 PE=1 SV=1 |
| 747 | tr\|F8VPD4\|F8VPD4_HUMAN | 29.63 | 236020 | CAD protein OS=Homo sapiens OX=9606 GN=CAD PE=1 SV=1 |
| 748 | sp\|P27708\|PYR1_HUMAN | 29.63 | 242981 | CAD protein OS=Homo sapiens OX=9606 GN=CAD PE=1 SV=3 |
| 749 | sp\|Q99879\|H2B1M_HUMAN | 29.61 | 13989 | Histone H2B type 1-M OS=Homo sapiens OX=9606 GN=HIST1H2BM PE=1 SV=3 |
| 750 | sp\|O60814\|H2B1K_HUMAN | 29.61 | 13890 | Histone H2B type 1-K OS=Homo sapiens OX=9606 GN=HIST1H2BK PE=1 SV=3 |
| 751 | sp\|Q16778\|H2B2E_HUMAN | 29.61 | 13920 | Histone H2B type 2-E OS=Homo sapiens OX=9606 GN=HIST2H2BE PE=1 SV=3 |
| 752 | sp\|Q93079\|H2B1H_HUMAN | 29.61 | 13892 | Histone H2B type 1-H OS=Homo sapiens OX=9606 GN=HIST1H2BH PE=1 SV=3 |
| 753 | sp\|P33778\|H2B1B_HUMAN | 29.61 | 13950 | Histone H2B type 1-B OS=Homo sapiens OX=9606 GN=HIST1H2BB PE=1 SV=2 |
| 754 | sp\|P62807\|H2B1C_HUMAN | 29.61 | 13906 | Histone H2B type 1-C/E/F/G/I OS=Homo sapiens OX=9606 GN=HIST1H2BC PE=1 SV=4 |
| 755 | sp\|P06899\|H2B1J_HUMAN | 29.61 | 13904 | Histone H2B type 1-J OS=Homo sapiens OX=9606 GN=HIST1H2BJ PE=1 SV=3 |
| 756 | sp\|Q99880\|H2B1L_HUMAN | 29.61 | 13952 | Histone H2B type 1-L OS=Homo sapiens OX=9606 GN=HIST1H2BL PE=1 SV=3 |
| 757 | sp\|Q8N257\|H2B3B_HUMAN | 29.61 | 13908 | Histone H2B type 3-B OS=Homo sapiens OX=9606 GN=HIST3H2BB PE=1 SV=3 |
| 758 | sp\|P58876\|H2B1D_HUMAN | 29.61 | 13936 | Histone H2B type 1-D OS=Homo sapiens OX=9606 GN=HIST1H2BD PE=1 SV=2 |
| 759 | sp\|P23527\|H2B1O_HUMAN | 29.61 | 13906 | Histone H2B type 1-O OS=Homo sapiens OX=9606 GN=HIST1H2BO PE=1 SV=3 |
| 760 | sp\|Q5QNW6\|H2B2F_HUMAN | 29.61 | 13920 | Histone H2B type 2-F OS=Homo sapiens OX=9606 GN=HIST2H2BF PE=1 SV=3 |
| 761 | sp\|Q99877\|H2B1N_HUMAN | 29.61 | 13922 | Histone H2B type 1-N OS=Homo sapiens OX=9606 GN=HIST1H2BN PE=1 SV=3 |
| 762 | sp\|P57053\|H2BFS_HUMAN | 29.61 | 13944 | Histone H2B type F-S OS=Homo sapiens OX=9606 GN=H2BFS PE=1 SV=2 |
| 763 | tr\|U3KQK0\|U3KQK0_HUMAN | 29.61 | 18804 | Histone H2B OS=Homo sapiens OX=9606 GN=HIST1H2BN PE=1 SV=1 |
| 764 | tr\|A0A2R8Y619\|A0A2R8Y619_HUMAN | 29.61 | 13491 | Histone H2B OS=Homo sapiens OX=9606 PE=1 SV=1 |
| 765 | sp\|Q96A08\|H2B1A_HUMAN | 29.61 | 14167 | Histone H2B type 1-A OS=Homo sapiens OX=9606 GN=HIST1H2BA PE=1 SV=3 |
| 766 | sp\|Q8N3Z3\|GTPB8_HUMAN | 29.49 | 32147 | GTP-binding protein 8 OS=Homo sapiens OX=9606 GN=GTPBP8 PE=2 SV=1 |
| 767 | tr\|A0A087WUP3\|A0A087WUP3_HUMAN | 29.49 | 34955 | GTP-binding protein 8 OS=Homo sapiens OX=9606 GN=GTPBP8 PE=1 SV=1 |
| 768 | tr\|Q5SYV2\|Q5SYV2_HUMAN | 29.47 | 40161 | Sarcosine dehydrogenase mitochondrial OS=Homo sapiens OX=9606 GN=SARDH PE=1 SV=1 |
| 769 | tr\|A0A0A0MT55\|A0A0A0MT55_HUMAN | 29.47 | 67216 | Sarcosine dehydrogenase mitochondrial OS=Homo sapiens OX=9606 GN=SARDH PE=1 SV=1 |
| 770 | sp\|Q9UL12\|SARDH_HUMAN | 29.47 | 101037 | Sarcosine dehydrogenase mitochondrial OS=Homo sapiens OX=9606 GN=SARDH PE=1 SV=1 |
| 771 | sp\|Q92922\|SMRC1_HUMAN | 29.47 | 122867 | SWI/SNF complex subunit SMARCC1 OS=Homo sapiens OX=9606 GN=SMARCC1 PE=1 SV=3 |
| 772 | sp\|Q9ULX6\|AKP8L_HUMAN | 29.36 | 71640 | A-kinase anchor protein 8-like OS=Homo sapiens OX=9606 GN=AKAP8L PE=1 SV=4 |
| 773 | tr\|V9GZ50\|V9GZ50_HUMAN | 29.36 | 47338 | A kinase (PRKA) anchor protein 8-like isoform CRA_c OS=Homo sapiens OX=9606 GN=AKAP8L PE=1 SV=1 |

| | | | | |
|---|---|---|---|---|
| 774 | tr\|A0A087WVM4\|A0A087WVM4_HUMAI | 29.27 | 99312 | Monofunctional C1-tetrahydrofolate synthase mitochondrial OS=Homo sapiens OX=9606 GN=MTHFD1L PE=1 SV=1 |
| 775 | sp\|Q6UB35\|C1TM_HUMAN | 29.27 | 105790 | Monofunctional C1-tetrahydrofolate synthase mitochondrial OS=Homo sapiens OX=9606 GN=MTHFD1L PE=1 SV=1 |
| 776 | tr\|B7ZM99\|B7ZM99_HUMAN | 29.27 | 105889 | MTHFD1L protein OS=Homo sapiens OX=9606 GN=MTHFD1L PE=1 SV=1 |
| 777 | tr\|A0A087WTT1\|A0A087WTT1_HUMAN | 29.04 | 58536 | Polyadenylate-binding protein OS=Homo sapiens OX=9606 GN=PABPC1 PE=1 SV=1 |
| 778 | tr\|E7EQV3\|E7EQV3_HUMAN | 29.04 | 65748 | Polyadenylate-binding protein OS=Homo sapiens OX=9606 GN=PABPC1 PE=1 SV=1 |
| 779 | sp\|P11940\|PABP1_HUMAN | 29.04 | 70671 | Polyadenylate-binding protein 1 OS=Homo sapiens OX=9606 GN=PABPC1 PE=1 SV=2 |
| 780 | tr\|H0YAR2\|H0YAR2_HUMAN | 29.04 | 32258 | Polyadenylate-binding protein 1 (Fragment) OS=Homo sapiens OX=9606 GN=PABPC1 PE=1 SV=1 |
| 781 | tr\|E7ERJ7\|E7ERJ7_HUMAN | 29.04 | 67139 | Polyadenylate-binding protein OS=Homo sapiens OX=9606 GN=PABPC1 PE=1 SV=1 |
| 782 | sp\|Q9H361\|PABP3_HUMAN | 29.04 | 70031 | Polyadenylate-binding protein 3 OS=Homo sapiens OX=9606 GN=PABPC3 PE=1 SV=2 |
| 783 | sp\|Q5VTR2\|BRE1A_HUMAN | 28.93 | 113662 | E3 ubiquitin-protein ligase BRE1A OS=Homo sapiens OX=9606 GN=RNF20 PE=1 SV=2 |
| 784 | sp\|Q9Y4C4\|MFHA1_HUMAN | 28.81 | 116950 | Malignant fibrous histiocytoma-amplified sequence 1 OS=Homo sapiens OX=9606 GN=MFHAS1 PE=1 SV=2 |
| 785 | tr\|D6RA20\|D6RA20_HUMAN | 28.81 | 97241 | Protocadherin alpha-4 OS=Homo sapiens OX=9606 GN=PCDHA4 PE=1 SV=2 |
| 786 | sp\|Q9UN74\|PCDA4_HUMAN | 28.81 | 102293 | Protocadherin alpha-4 OS=Homo sapiens OX=9606 GN=PCDHA4 PE=1 SV=1 |
| 787 | tr\|A0A087WSW8\|A0A087WSW8_HUMAI | 28.81 | 83615 | Protocadherin alpha-4 OS=Homo sapiens OX=9606 GN=PCDHA4 PE=1 SV=1 |
| 788 | sp\|P05154\|IPSP_HUMAN | 28.75 | 45675 | Plasma serine protease inhibitor OS=Homo sapiens OX=9606 GN=SERPINA5 PE=1 SV=3 |

**Supplementary Table 2.** The correlations between ARHGAP24 expression and clinicopathological characteristics in HCC.

| | | | ARHGAP24 expression | | |
|---|---|---|---|---|---|
| | | | low (85) | High (46) | P |
| Gender | Male | 27 | 18 (66.7) | 9 (33.3) | 0.828 |
| | Female | 104 | 67 (64.4) | 37 (35.6) | |
| Age | ≤50 | 39 | 28 (71.8) | 11 (28.2) | 0.281 |
| | >50 | 92 | 57 (62.0) | 35 (38.0) | |
| Liver cirrhosis | No | 64 | 45 (70.3) | 19 (29.7) | 0.203 |
| | Yes | 67 | 40 (59.7) | 27 (40.3) | |
| HBsAg | No | 27 | 17 (63.0) | 10 (37.0) | 0.814 |
| | Yes | 104 | 68 (65.4) | 36 (34.6) | |
| ALT,U/L | ≤40 | 101 | 62 (61.4) | 39 (38.6) | 0.124 |
| | >40 | 30 | 23 (76.7) | 7 (23.3) | |
| AST,U/L | ≤40 | 88 | 55 (62.5) | 33 (37.5) | 0.413 |
| | >40 | 43 | 30 (69.8) | 13 (30.2) | |
| AFP, ng/ml | ≤400 | 91 | 57 (62.6) | 34 (37.4) | 0.416 |
| | >400 | 40 | 28 (70.0) | 12 (30.0) | |
| Tumor number | Single | 104 | 66 (63.5) | 38 (36.5) | 0.503 |
| | Multiple | 27 | 19 (70.4) | 8 (29.6) | |
| Tumor size, cm | ≤5 | 73 | 43 (58.9) | 30 (41.1) | 0.108 |
| | >5 | 58 | 42 (72.4) | 16 (27.6) | |
| Satellite lesion | No | 114 | 70 (61.4) | 44 (38.6) | **0.031** |
| | Yes | 17 | 15 (88.2) | 2 (11.8) | |
| MVI | No | 62 | 31 (50.0) | 31 (50.0) | **0.001** |
| | Yes | 69 | 54 (78.3) | 15 (21.7) | |
| Edmondson stage | I-II | 62 | 38 (60.9) | 24 (39.1) | 0.414 |
| | III-IV | 69 | 47 (68.1) | 22 (31.9) | |
| BCLC stage | 0+A | 100 | 61 (61.0) | 39 (39.0) | 0.094 |
| | B+C | 31 | 24 (77.4) | 7 (22.6) | |
| CNLC stage | I-II | 117 | 72 (61.5) | 45 (38.5) | **0.020** |
| | III-IV | 14 | 13 (92.9) | 1 (7.1) | |
| Tumor recurrence | No | 79 | 43 (54.4) | 36 (45.6) | **0.002** |
| | Yes | 52 | 42 (80.8) | 10 (19.2) | |

Abbreviations: ALT, alanine aminotransferase; AST, aspartate transaminase; AFP, α-fetoprotein; MVI, Microvascular invasion; BCLC, Barcelona Clinic Liver Cancer; CNLC, China liver cancer staging.

**Supplementary Table 3.** Univariate cox proportional regression analysis of factors associated with recurrence and overall survival

| Variables | Tumor Recurrence | |
|---|---|---|
| | HR (95% CI) | P |
| Sex<br>(Male versus female) | 0.69<br>(0.37-1.30) | 0.255 |
| Age<br>(>50 versus ≤50) | 1.37<br>(0.73-2.56) | 0.331 |
| HBsAg<br>(Yes versus No) | 1.16<br>(0.57-2.38) | 0.683 |
| Liver cirrhosis<br>(Yes versus no) | 1.01<br>(0.58-1.73) | 0.986 |
| γ-GT, U/L<br>(>45 versus ≤45) | 2.55<br>1.38-4.71 | **0.003** |
| ALT<br>(>40U/L versus ≤40U/L) | 1.48<br>(0.81-2.69) | 0.205 |
| AST<br>(>40U/L versus ≤40U/L) | 2.37<br>(1.37-4.09) | **0.002** |
| AFP<br>(>400ng/ml versus ≤400ng/ml) | 1.86<br>(1.07-3.24) | **0.028** |
| No. of tumors<br>(Multi versus single) | 1.96<br>(1.09-3.54) | **0.025** |
| Tumor size<br>(>5cm versus ≤5cm) | 3.06<br>(1.73-5.39) | **0.000** |
| Micro vascular invasion<br>(Yes versus no) | 1.05<br>(0.61-1.82) | 0.852 |
| Satellite lesion<br>(Yes versus no) | 4.96<br>(2.64-9.31) | **0.000** |
| Edmondson stage<br>(III-IV versus I-II) | 1.29<br>(0.75-2.24) | 0.358 |
| BCLC stage<br>(B+C versus 0+A) | 3.54<br>(2.03-6.16) | **0.000** |
| ARHGAP24<br>(High versus low) | 0.34<br>(0.17-0.68) | **0.002** |

Abbreviations: ALT, alanine aminotransferase; AST, aspartate transaminase; AFP, α-fetoprotein; BCLC, Barcelona Clinic Liver Cancer; HR, hazard ratio.

## Methods and Materials

### Cell culture

Huh7, Li-7, and Hep3B cells were purchased from the Chinese Academy of Sciences Shanghai Branch Cell Bank (Shanghai, China). L02, MHCC97L, MHCC97H, and HCCLM3 cells were gifted by Professor Yang at the Liver Cancer Institute of Zhongshan Hospital, Fudan University. The cell lines were cultured in DMEM medium (Gibco, Grand Island, NY, USA) supplemented with 10% fetal bovine serum (Gibco) and penicillin/streptomycin, and maintained in an incubator at 37°C containing 5% $CO_2$.

### Generation of lentivirus and plasmid construction

Wild-type ARHGAP24 for overexpression of ARHGAP24 (ARH-OE cells) and negative control vector (Vector) were transfected into HCCLM3 and Huh7 cells, based on the GV492 vector (Ubi-MCS-3Flag-CBh-IRES-puromycin) purchased from Shanghai Genechem Corporation (Shanghai, China). Knockdown lentiviral vector (shARH#1 and shARH#2) and negative control vector (NC) were transfected into Li-7 cells, based on the GV493 vector (Hu6-MCS-CBh-gcGFP-IRES-puromycin) purchased from Shanghai Genechem Corporation. SiPKM2 and siWWP1 were constructed by Shanghai GenePharma Corporation (Shanghai, China).

Full-length ARHGAP24, PKM2, and WWP1 plasmids were obtained by cloning cDNA into a phage vector with a corresponding HA, FLAG, or His tag. The GAP-deficient mutant of ARHGAP24 (HA-Q158R) and a series of ARHGAP24 mutations that lack different domains, including HA-ARH-PHD, HA-ARH-GAPD, HA-ARH-CD, and HA-ARH-ΔCD, and deletion mutants of PKM2, including Flag-PKM2-ΔCD, Flag-PKM2-ABD, and Flag-PKM2-CD, and deletion mutants of ARHGAP24 were constructed by Shanghai Genechem Corporation. ARHGAP24 mutants, including M1, M2, M3, and M4, were also designed by

Shanghai Genechem Corporation.

**Western blot and Immunofluorescence**

Western blotting and immunofluorescence assays were performed as described previously [1]. Briefly, for western blot assays, proteins were extracted from the indicated cells and fractionated by SDS-PAGE. Then, proteins were transferred to nitrocellulose membranes. After blocking with 5% milk at room temperature for 1 h, the membranes were incubated with the specific primary antibodies overnight. For immunofluorescence assay, the cells were fixed, permeabilized, and blocked. Then, the indicated primary antibodies were incubated with cells. The next day, the membranes and cells were washed three times with PBS and incubated with a horseradish peroxidase-conjugated secondary antibody and fluorescently labeled secondary antibody, respectively. To observe the cytoskeleton, phalloidin was used for immunofluorescence staining.

**Immunohistochemistry**

Immunohistochemical staining was performed as described previously [1]. Briefly, HCC tissues underwent the following steps: dewaxing treatment, antigen retrieval, and endogenous peroxidase blockade. The primary antibodies and dilutions used to detect the intensities of the indicated markers are listed below. Immunostaining intensities of the indicated markers were evaluated as follows: negative, weak, moderate and strong. When the intensity was negative or weak, the expression status was defined as “low expression”, while the intensity was moderate or strong, the expression status was defined as “high expression”. At least two pathology-related professionals interpreted the slices in a double-blind manner. If the results of the two personnel were disputed, other related pathology professionals examined the slice again. After discussion by all personnel, the final immunostaining group was determined.

**Immunoprecipitation**

The indicated plasmids were co-transfected into Li-7, HCCLM3, and HEK293T cells. After 48 h, cells were lysed with IP lysis buffer (20mM Tris-HCL, pH7.4, 150mM NaCL, 1mM EDTA, and 1% NP-40) containing protease inhibitor cocktail (Roche, USA). Immunoprecipitation assays were performed following the manufacturer's instructions (Pierce, Thermo, USA). The supernatant protein was immunoprecipitated with the indicated tagged antibodies and protein G agarose beads overnight at 4°C. Antibodies used in these experiments are listed below. The complex was washed with NaCl buffer and boiled with 1×SDS loading buffer.

**Antibodies and reagents**

Antibodies used in this study were ARHGAP24 (PA5-104104; Invitrogen), β-tubulin (Beyotime, Shanghai), PCNA (13110; Cell Signaling Technology), cleaved-caspase3 (9664; Cell Signaling Technology), cyclin D1 (55506; Cell Signaling Technology), BCL2 (15071; Cell Signaling Technology), CDK2 (18048; Cell Signaling Technology), E-cadherin (3195; Cell Signaling Technology), N-cadherin (13116; Cell Signaling Technology), vimentin (5741; Cell Signaling Technology), α-SMA (19245; Cell Signaling Technology), MMP-9 (13667; Cell Signaling Technology), Ki67 (9449; Cell Signaling Technology), β-catenin (8480; Cell Signaling Technology), c-MYC (18583; Cell Signaling Technology), PKM2 (4053; Cell Signaling Technology), WWP1 (ab104440; Abcam), anti-FLAG (14793; Cell Signaling Technology), anti-HA (3724; Cell Signaling Technology), and anti-His (12698; Cell Signaling Technology).

**Cell proliferation, migration, and invasion assays**

CCK8 and colony formation assays were used to investigate cell proliferation, which were performed as described previously [1]. Cell migration and invasion were detected using transwell chambers (BD, PharMingen, San Jose, CA), as

previously described [51]. Briefly, $5 \times 10^4$ cells were plated in the top chamber with Matrigel used or not, and the lower chamber was supplemented with 500 μL complete medium and incubated for 24 h. The next day, after fixing with methanol and staining with crystal violet, the cells on the membrane were counted on three inserts. Cell migration was also investigated by cell scratching assay, as previously described [1].

**Cell cycle and cell apoptosis assays**

Flow cytometry (Aria II flow cytometer, BD, USA) was performed to analyze cell cycle and cell apoptosis, as described previously. For cell cycle analysis, cells were harvested and fixed with 70% ethanol overnight at 4°C. The cells were then washed three times, and 50 μg/mL PI, 100 μg/mL RNase A, and 0.2% Triton X-100 were added to each sample. For cell apoptosis analysis, control cells and experimental cells were seeded into 24-well plates and cultured for 48 h. Then, the cells were collected, resuspended, and treated with the Annexin V-FITC Apoptosis Detection Kit (BD, PharMingen, San Jose, CA) following the manufacturer's instructions. Briefly, 5 μL FITC-labeled Annexin V and 5 μL PI solution were added to each sample, and the cells were incubated for 15 min at room temperature.

**In vivo assays**

Six-week-old BALB/C nude mice were purchased from the Shanghai Institute of Material Medicine of the Chinese Academy of Science (Shanghai, China). The committee on the use of live animals in teaching and research at Zhongshan Hospital, Fudan University, approved all animal experiments. Li-7-Ctrl, Li-7-shARH, HCCLM3-vector, and HCCLM3-ARH-OE cells ($5 \times 10^6$) were suspended in 200 μL serum-free DMEM and Matrigel (BD Biosciences; 1:1), and then injected subcutaneously into the flanks of nude mice. After 4 weeks, when the tumor volume had grown to 1 $cm^3$, the mice were killed and the tumor tissue was stripped. Then, the center area of the tumor tissue was selected and

the necrotic tissue was removed. The tumor tissue was then cut to a size of 1–2 $mm^3$ for subsequent orthotopic transplantation tumor model construction. A total of 24 nude mice were selected and randomly assigned to four groups. After completion of the transplantation for 6 weeks, the mice were killed. The volume and weight of the tumor were measured. At the same time, the liver cancer tissues were embedded in paraffin for subsequent IHC analysis.

For tumor metastasis analysis, 6- to 8-week-old nude mice were selected and randomly assigned to four groups. Mice were injected with $2 \times 10^6$ of the indicated cells via the tail vein. After 8 weeks, the lung tissues were separated and stained with HE. The numbers of tumor metastasis were recorded.

**Ubiquitination analysis**

The indicated plasmids were co-transfected into Li-7, HCCLM3, and HEK293T cells. After incubation for 24 h, the cells were treated with 10 μM MG132 for 6 h. Then, the cells were lysed with IP lysis buffer and boiled at 100°C for 10 min. The supernatants were collected and RIPA lysis was then added to PMSF and protease inhibitor cocktail for IP with anti-PKM2, anti-FLAG, anti-HA, or anti-His.

**Bioinformatics**

For identification of DEGs and pathway enrichment, the FPKM and count expression profiles were obtained from the TCGA database (https://portal.gdc.cancer.gov/), UCSC Xena website (http://xena.ucsc.edu/), GEO database(https://www.ncbi.nlm.nih.gov/geo/) and the Clinical Proteomic Tumor Analysis Consortium (https://proteomic.datacommons.cancer.gov/pdc/). “limma” R package and GEO2R online tools were employed to perform differential expression analysis between tumor tissue and tumor adjacent tissues in the GSE164760, GSE76427, GSE101728, GSE101685, TCGA and CPTAC cohorts. TCGA-LIHC samples were divided into ARHGAP24 high and low expression groups based on the upper and lower quartiles of ARHGAP24

expression. Genes with an expression- p value $< 0.05$ and |log2 fold change (FC)| >1 were regarded as significant DEGs using “limma” R package. To explore whether genes in ARHGAP24 high and low expression groups were enriched in meaningful biological processes, gene set enrichment analysis was performed in the two groups (www.gsea-msigdb.org/gsea/index.jsp). The annotated gene set c2.cp.reactome.v7.5.1.symbols.gmt [Curated] was selected as the reference gene set. Visual analysis of data was generated by “ggplot2”, ”pheatmap” and “enrichplot” R package. Follow-up data of patients with various cancers were acquired from the K-M plot database (http://kmplot.com/analysis). The CPTAC cohort follow-up information was acquired from the website https://proteomics.cancer.gov/programs/cptac.

For the analysis of the interaction between PKM2 and ARHGAP24, the three-dimensional protein structures of PKM (Uniport ID: P14618-1) and ARHGAP24 (Uniport ID: Q8N264-1) were established by the comprehensive use of multiple protein three-dimensional structure modeling software, including I-TASSER, FR-t5-M, and FALCON. The binding amino acid positions in the constructed PKM2 and ARHGAP24 three-dimensional protein structures were predicted by tools such as SPR and ISPRED4, respectively. According to the protein interaction sites, the optimal docking model was identified using HDOCK. MEFTop and QIPI were also used to evaluate the docking conformation. ContPro was used to calculate the spatial distance of each atom in the docking model, and 4Å was selected as the threshold of the distance between atoms to select the amino acids that may have interactions in the docking model.

**TOP/FOP FLASH assays**

TOP/FOP flash analysis was performed in accordance with the manufacturer’s protocol (Sigma–Aldrich, USA). TOP flash (TCF reporter plasmid) and FOP flash (mutant TCF binding sites) were used. The indicated HCC cell lines were co-transfected with 100 ng reporter plasmid, 50 ng expression plasmid, and 8

ng *Renilla* luciferase plasmid with Lipo2000 in a 96-well plate. After 24 h, the luciferase reporters TOP/FOP FLASH were combined with the various expression plasmids. Finally, the indicated cells were analyzed using a luciferase reporter assay kit (Promega) and a microplate reader.

**Reference**

[1] Yang W, Zhang C, Li Y, et al. Phosphorylase Kinase β Represents a Novel Prognostic Biomarker and Inhibits Malignant Phenotypes of Liver Cancer Cell. Int J Biol Sci. 2019;15(12):2596-2606.

**The targeting sequences of small hairpin RNAs were used in study.**

| Gene | Target sequences |
| --- | --- |
| shRNA#1 against ARHGAP24 | 5′- ccGAGAGAGGAAACACAATAT -3′ |
| shRNA#2 against ARHGAP24 | 5′- ccAGCAGTTGATGTCAGTGAT-3′ |
| Scrambled shRNA | 5’- TTCTCCGAACGTGTCACGT-3’ |
| SiPKM2#1 | Sense: 5’- GGAUGUUGAUAUGGUGUUUTT-3’<br>Anti-sense: 5’- AAACACCAUAUCAACAUCCTT-3’ |
| SiPKM2#2 | Sense: 5’- CCAUCUACCACUUGCAAUUTT-3’<br>Anti-sense: 5’- AAUUGCAAGUGGUAGAUGGTT-3’ |
| SiWWP1#1 | 5’-GAAGTCATCTGTAACTAAA-3′ |
| SiWWP1#2 | 5’-GCAGAGAAATACTGTTTAT-3′ |

**The primer sequences were used in study.**

| Gene | | Sequences |
| --- | --- | --- |
| ARHGAP24 | Forward | 5′- GAACCGTCTGGCTCCGATG -3′ |
| | Reverse | 5′- TGGCAGTCGAAAGAGACCCT -3′ |
| CTNNB1 | Forward | 5′- AAAGCGGCTGTTAGTCACTGG-3′ |
| | Reverse | 5′- CGAGTCATTGCATACTGTCCAT-3′ |
| PKM2 | Forward | 5’- ATGTCGAAGCCCCATAGTGAA-3’ |
| | Reverse | 5’- TGGGTGGTGAATCAATGTCCA-3’ |
| E-cadherin | Forward | 5’- CGAGAGCTACACGTTCACGG -3’ |
| | Reverse | 5’- GGGTGTCGAGGGAAAAATAGG-3’ |
| N-cadherin | Forward | 5’- TCAGGCGTCTGTAGAGGCTT-3’ |
| | Reverse | 5’- ATGCACATCCTTCGATAAGACTG-3’ |
| vimentin | Forward | 5’- GACGCCATCAACACCGAGTT-3’ |
| | Reverse | 5’- CTTTGTCGTTGGTTAGCTGGT-3’ |
| snail | Forward | 5′- TCGGAAGCCTAACTACAGCGA-3′ |
| | Reverse | 5′- AGATGAGCATTGGCAGCGAG-3′ |
| slug | Forward | 5′- CGAACTGGACACACATACAGTG-3′ |
| | Reverse | 5’- CTGAGGATCTCTGGTTGTGGT-3’ |
| c-myc | Forward | 5’- GGCTCCTGGCAAAAGGTCA-3’ |
| | Reverse | 5’- CTGCGTAGTTGTGCTGATGT-3’ |
| cyclinD1 | Forward | 5’- GCTGCGAAGTGGAAACCATC-3’ |
| | Reverse | 5’- CCTCCTTCTGCACACATTTGAA-3’ |
| β-actin | Forward | 5’- CATGTACGTTGCTATCCAGGC-3’ |
| | Reverse | 5’- CTCCTTAATGTCACGCACGAT-3’ |
